# Pain Coping Skills Training for Patients Receiving Hemodialysis: The HOPE Consortium Randomized Clinical Trial

**DOI:** 10.1001/jamainternmed.2024.7140

**Published:** 2024-12-30

**Authors:** Laura M. Dember, Jesse Y. Hsu, Rajnish Mehrotra, Kerri L. Cavanaugh, Sahir Kalim, David M. Charytan, Michael J. Fischer, Manisha Jhamb, Kirsten L. Johansen, William C. Becker, Bethany Pellegrino, Nwamaka D. Eneanya, Sarah J. Schrauben, Patrick H. Pun, Mark L. Unruh, Benjamin J. Morasco, Mansi Mehta, Nobuyuki Miyawaki, Jeffrey Penfield, Leah Bernardo, Carrie E. Brintz, Martin D. Cheatle, Ardith Z. Doorenbos, Alicia A. Heapy, Francis J. Keefe, Erin E. Krebs, Natalie Kuzla, Sagar U. Nigwekar, Rebecca J. Schmidt, Jennifer L. Steel, James B. Wetmore, David M. White, Paul L. Kimmel, Daniel Cukor, and the HOPE Consortium

**Affiliations:** 1 Renal-Electrolyte and Hypertension Division, Department of Medicine, University of Pennsylvania Perelman School of Medicine, Philadelphia; 2 Department of Biostatistics, Epidemiology and Informatics, Center for Clinical Epidemiology and Biostatistics, University of Pennsylvania Perelman School of Medicine, Philadelphia; 3 Kidney Research Institute, Division of Nephrology, Department of Medicine, University of Washington, Seattle; 4 Division of Nephrology and Hypertension, Department of Medicine, Vanderbilt University Medical Center, Nashville, Tennessee; 5 Division of Nephrology, Department of Medicine, Massachusetts General Hospital and Harvard Medical School, Boston; 6 Division of Nephrology, Department of Medicine, New York University Grossman School of Medicine, New York, New York; 7 Department of Internal Medicine, University of Illinois Hospital and Health Sciences Center, Chicago; 8 Medical Service, Jesse Brown VA Medical Center, Chicago, Illinois; 9 Center of Innovation for Complex Chronic Healthcare, Edward Hines, Jr. VA Hospital, Hines, Illinois; 10 Renal-Electrolyte Division, Department of Medicine, University of Pittsburgh School of Medicine, Pittsburgh, Pennsylvania; 11 Division of Nephrology, Department of Medicine, Hennepin Healthcare, Minneapolis, Minnesota; 12 Department of Medicine, University of Minnesota Medical School, Minneapolis; 13 Section of General Internal Medicine, Department of Medicine, Yale School of Medicine, New Haven, Connecticut; 14 Pain Research, Informatics, Multi-morbidities and Education Center of Innovation, VA Connecticut Healthcare System, West Haven; 15 Division of Nephrology, Department of Medicine, West Virginia University School of Medicine, Morgantown; 16 Now with Division of Renal Medicine, Department of Medicine, Emory University School of Medicine, Atlanta, Georgia; 17 Division of Nephrology, Department of Medicine, Duke University School of Medicine, Durham, North Carolina; 18 Medical Service, Durham VA Medical Center, Durham, North Carolina; 19 Division of Nephrology, Department of Internal Medicine, School of Medicine, University of New Mexico, Albuquerque; 20 Center to Improve Veteran Involvement in Care, VA Portland Health Care System, Portland, Oregon; 21 Department of Psychiatry, Oregon Health & Science University, Portland; 22 VA New York Harbor Healthcare, New York; 23 Division of Nephrology, New York University Grossman Long Island School of Medicine, Mineola, New York; 24 The University of Texas Southwestern Medical Center, Dallas; 25 VA North Texas Health Care System, Dallas; 26 Division of Pain Medicine, Department of Anesthesiology, Vanderbilt Center for Musculoskeletal Research, Osher Center for Integrative Health at Vanderbilt, Vanderbilt University Medical Center, Nashville, Tennessee; 27 Department of Psychiatry, University of Pennsylvania Perelman School of Medicine, Philadelphia; 28 Department of Anesthesiology and Critical Care, University of Pennsylvania Perelman School of Medicine, Philadelphia; 29 Department of Biobehavioral Nursing Science, College of Nursing, University of Illinois Chicago, Chicago; 30 Department of Anesthesiology and Pain Medicine, University of Washington, Seattle; 31 Department of Psychiatry, Yale School of Medicine, New Haven, Connecticut; 32 Pain Prevention and Treatment Research Program, Department of Psychiatry and Behavioral Sciences, Duke University School of Medicine, Durham, North Carolina; 33 Center for Care Delivery and Outcomes Research, Minneapolis VA Health Care System, Minneapolis, Minnesota; 34 Division of General Internal Medicine, University of Minnesota Medical School, Minneapolis; 35 Clinical Research Collaboration Unit, Center for Clinical Epidemiology and Biostatistics, Perelman School of Medicine, University of Pennsylvania, Philadelphia; 36 Division of Hepatobiliary Surgery, Department of Surgery, University of Pittsburgh School of Medicine, Pittsburgh, Pennsylvania; 37 Department of Psychiatry, University of Pittsburgh School of Medicine, Pittsburgh, Pennsylvania; 38 Department of Psychology, University of Pittsburgh, Pittsburgh, Pennsylvania; 39 American Association of Kidney Patients, Tampa, Florida; 40 National Institute of Diabetes and Digestive and Kidney Diseases, National Institutes of Health, Bethesda, Maryland; 41 The Rogosin Institute, New York, New York; 42 Now with Division of Nephrology, Department of Medicine, New York University Grossman School of Medicine, New York

## Abstract

**Importance:**

Chronic pain is common among individuals with dialysis-dependent kidney failure.

**Objective:**

To evaluate the effectiveness of pain coping skills training (PCST), a cognitive behavioral intervention, on pain interference.

**Design, Setting, and Participants:**

This multicenter randomized clinical trial of PCST vs usual care was conducted across 16 academic centers and 103 outpatient dialysis facilities in the US. Adults undergoing maintenance hemodialysis and experiencing chronic pain were randomly assigned to PCST or usual care in a 1:1 ratio. Participants were followed in the trial for 36 weeks. Enrollment began on January 4, 2021, and follow-up ended on December 21, 2023.

**Interventions:**

PCST consisting of 12 weekly coach-led sessions via video or telephone conferencing, followed by 12 weeks of daily interactive voice response sessions. Usual care had no trial-driven pain intervention.

**Main Outcomes:**

The primary outcome was pain interference measured with the Brief Pain Inventory (BPI) Interference subscale (score range of 0-10, with higher scores indicating more pain interference). Secondary outcomes included pain intensity, pain catastrophizing, quality of life, depression, and anxiety.

**Results:**

A total of 643 participants (mean [SD] age, 60.3 [12.6] years; 288 [44.8%] female) were randomized, with 319 assigned to PCST and 324 assigned to usual care. At week 12 (primary end point), the PCST group had a larger reduction in the BPI Interference score than the usual care group (between-group difference, −0.49; 95% CI, −0.85 to −0.12; *P* = .009). The effect persisted at week 24 (between-group difference in BPI Interference score, −0.48; 95% CI, −0.86 to −0.11) but was diminished at week 36 (between-group difference in BPI Interference score, −0.34; 95% CI, −0.72 to 0.04). A decrease in BPI Interference score greater than 1 point (minimal clinically important difference) occurred in 143 of 281 participants (50.9%) in the PCST group vs 108 of 295 participants (36.6%) in the usual care group at 12 weeks (odds ratio, 1.79; 95% CI, 1.28-2.49) and 142 of 258 participants (55.0%) in the PCST group vs 113 of 264 participants (42.8%) in the usual care group at 24 weeks (odds ratio, 1.59; 95% CI, 1.13-2.24). Favorable changes with PCST were also apparent for secondary outcomes of pain intensity, quality of life, depression, and anxiety at weeks 12 and/or 24, as well as for pain catastrophizing at weeks 24 and 36.

**Conclusions and Relevance:**

In this randomized clinical trial of patients undergoing maintenance hemodialysis, PCST had benefits on pain interference and other pain-associated outcomes. While the effect on the overall cohort was of modest magnitude, the intervention resulted in a clinically meaningful improvement in pain interference for a substantial proportion of participants.

**Trial Registration:**

ClinicalTrials.gov Identifier: NCT04571619

## Key Points

**Question:**

Does pain coping skills training (PCST), a cognitive behavioral intervention, reduce pain interference among people receiving maintenance hemodialysis?

**Findings:**

In this randomized clinical trial that included 643 adults receiving maintenance hemodialysis, there was a modest, but statistically significant, beneficial effect of PCST compared with usual clinical care on pain interference as measured by the Brief Pain Inventory Interference subscale at 12 weeks. The effect persisted at 24 weeks but was attenuated at 36 weeks.

**Meaning:**

PCST modestly improved pain interference and other pain-associated outcomes among people with kidney failure receiving maintenance hemodialysis.

## Teaser

This randomized clinical trial evaluates the effectiveness of pain coping skills training, a cognitive behavioral intervention, on pain interference among people receiving maintenance hemodialysis.

## Introduction

Maintenance hemodialysis is a life-extending treatment for people with kidney failure, but many patients have a high burden of symptoms that contribute to a poor quality of life.^1,2,3^ Management of pain, one of the symptoms reported most frequently by this patient population, is challenging in the setting of kidney failure because of heterogeneous etiologies of pain, altered pharmacokinetic properties of medications, and frequent hospitalizations that interrupt treatments.^4,5^

Behavioral approaches for chronic pain such as cognitive behavioral therapy are appealing alternatives or complements to pharmacologic therapies but have not been well studied among people with dialysis-dependent kidney failure.^5,6^ A randomized trial that enrolled 160 patients receiving maintenance hemodialysis who had fatigue, pain, or depression showed improvements in patient-reported outcomes (PROs) with a stepped collaborative care intervention that included cognitive behavioral therapy.^7^ However, only a subset of the participants had chronic pain, and the intervention included components that might not be widely implementable in the typical outpatient dialysis setting. We conducted a multicenter randomized clinical trial of pain coping skills training (PCST) to evaluate the effects of a cognitive behavioral therapy–based approach for patients being treated with maintenance hemodialysis.^8,9,10^

## Methods

### Design

The primary hypothesis of the HOPE Consortium Trial to Reduce Pain and Opioid Use in Hemodialysis was that PCST would improve pain interference compared with usual clinical care. Details of the design and methods were previously published and are provided in the protocol (Supplement 1) and statistical analysis plan (Supplement 2).^11^ The trial included a randomized comparison of PCST for 24 weeks vs usual care, followed by an exploratory, nonrandomized evaluation of buprenorphine as an alternative to full-agonist opioid medications for the subset of participants receiving opioid medication at 20 morphine milligram equivalents per day or higher.^5,12,13^ Follow-up for participants was 36 weeks. In this report, we provide results of the randomly assigned PCST intervention on the primary and major secondary outcomes.

The trial was funded by the National Institute of Diabetes and Digestive and Kidney Diseases (NIDDK) through the National Institutes of Health Helping to End Addiction Long-term Initiative.^14,15^ The institutional review board (IRB) at the University of Pennsylvania served as the IRB of record for the 11 non–US Department of Veterans Affairs (VA) enrolling sites, and the VA Central IRB served as the IRB of record for the 5 VA enrolling sites. An independent Data and Safety Monitoring Board, appointed by the NIDDK, approved the protocol and reviewed safety, progress, and data quality. An advisory panel of individuals with kidney failure provided input throughout the planning and conduct of the trial. The trial was designed by the investigators with input from the NIDDK project scientist (P.L.K.). Trial data were collected by the investigators and research coordinators at the enrolling centers and analyzed by the data coordinating center. Participants provided written informed consent. This study followed the Consolidated Standards of Reporting Trials (CONSORT) reporting guidelines.

### Setting and Eligibility Criteria

The trial was conducted at 16 academic centers (enrolling sites) and 103 outpatient dialysis facilities in the US. The full list of eligibility criteria is provided in eTable 1 in Supplement 3. The major inclusion criteria were age of 18 years and older, treatment with in-center maintenance hemodialysis for 90 days or longer, English or Spanish fluency, and self-report of moderate or severe chronic pain, defined as pain on most days or every day during the past 3 months and a score of 4 or higher on a scale of 0 to 10 on the Pain, Enjoyment of Life, and General Activity scale.^16^ The major exclusion criteria were current substance use disorder, suicidal intent, substantial cognitive impairment, anticipated change in kidney replacement modality within 6 months, and life expectancy shorter than 6 months. Patients self-reported sex and race and ethnicity (including American Indian or Alaska Native, Asian, Black, Hispanic or Latino, Native Hawaiian or Other Pacific Islander, White, and multiracial).

### Randomization and Blinding

Participants were randomly assigned to PCST or usual care in a 1:1 ratio using random permuted blocks of 2, 4, and 6 with stratification by enrolling site and presence or absence of opioid use. Baseline PRO questionnaires were administered before randomization. Participants and site research team members were not masked to the treatment assignment. PROs were ascertained by a centralized team of questionnaire administrators masked to treatment assignment.

### Interventions

Participants randomized to PCST had weekly coach-led sessions lasting 45 to 50 minutes via video or telephone conferencing for 12 weeks, followed by an additional 12 weeks of automated interactive voice response (IVR) sessions as previously described.^11^ The content was adapted from a traditional PCST program to include modules addressing pain-related anxiety, stress, and sleep difficulties but maintained the components of pain education, experiential training, and an overarching goal of enhancing self-efficacy for applying the acquired coping skills.^8,9^ The location (dialysis facility vs home or other location) for the coach-led sessions was based on the participant’s preference. Video conferencing was the recommended delivery method, with telephone conferencing permitted if preferred by the participant. IVR consisted of daily automated telephone calls to monitor progress through participants’ keypad responses to questions and to provide access to skills refresher activities.^17,18^ Asynchronous feedback from the coach was provided weekly through the subsequent IVR calls. Each participant was assigned a single coach from a group of 6 for the full duration of the intervention. Each coach had a master’s degree in either social work or counseling and prior experience providing service to medical or psychiatric populations. The coaches participated in training prior to trial initiation and supervision throughout the duration of intervention administration, as detailed in eTable 2 in Supplement 3. Participants in both the PCST and usual care groups were given an educational brochure about chronic pain at the baseline visit, and both groups received care for pain and other conditions from their clinical care health care professionals, as they would in the absence of trial participation. At week 24, participants in both groups had eligibility assessed for the exploratory buprenorphine intervention. Those who met the eligibility criteria were encouraged to switch from the full-agonist opioid to buprenorphine.^11^

### Follow-Up Visits

Participants had visits with the research team every 4 weeks in person or by telephone. These visits were conducted to ascertain adverse events and clinical outcomes, update medication records, and schedule the PRO ascertainment calls.

### Outcomes

The primary outcome was pain-related interference with function and quality of life domains, as assessed by the Brief Pain Inventory (BPI) Interference subscale (score range of 0-10, with higher scores indicating more pain interference).^19^ Secondary outcomes included additional patient-reported indicators of pain and opioid use, conditions or symptoms that often accompany pain, and clinical events potentially related to pain or pain treatments (eTable 3 in Supplement 3). From the full set of secondary outcomes, the following were prespecified to be included in the report of the primary results: pain intensity,^19^ pain catastrophizing,^20^ quality of life,^21^ depression,^22^ anxiety,^23^ opioid use assessed using the timeline followback approach,^24^ a composite outcome of pain interference and opioid use as defined in eTable 3 in Supplement 3, falls, hospitalizations, and death. Responses to the centrally administered PRO questionnaires were not accessible to site research team members or the PCST interventionists.

### Statistical Analysis

The primary analysis compared the change from baseline to week 12 in the BPI Interference score between the PCST and usual care groups. An intent-to-treat approach was used in which all available data on all randomized participants were included in a linear mixed-effects model that incorporated, as fixed effects, the intervention, study week, interactions between intervention and study week, and baseline opioid use, and, as random effects, enrolling site and participants within each enrolling site.^25^ The same modeling approach was used to compare change in BPI Interference score from baseline to week 24 to evaluate the effect of the full PCST intervention and from baseline to week 36 to evaluate durability of the effect. This modeling approach was also used for the continuous secondary outcomes. The proportion of participants with a reduction from baseline in the BPI Interference score of more than 1 point, considered in multiple nondialysis patient populations to be the minimal clinically important difference,^26^ was compared between PCST and usual care using a generalized estimating equation (GEE) model with a binomial distribution and a logit link.^27^ The model included intervention, time, interactions between intervention and time, and baseline opioid use, and treated participants as clusters with an exchangeable correlation structure. This modeling approach was also used for a post hoc analysis comparing the proportions of participants in the PCST and usual care groups with a reduction from baseline in the BPI Interference score of more than 30%, a threshold based on relative rather than absolute change that is frequently used to evaluate pain interventions.^26^

For the secondary outcome of death and the recurrent secondary outcomes of falls and hospitalizations, GEE models with a Poisson distribution, an offset of follow-up time, and a log link were used to compare event rates. The Poisson GEE models included intervention and baseline opioid use and treated enrolling sites as clusters with an independent correlation structure. The use of Poisson GEE models with robust standard errors was anticipated to mitigate the effect of overdispersion for the outcomes of falls and hospitalizations. A sensitivity analysis was performed using negative binomial GEE models to assess the robustness of the findings and the effect of overdispersion.

Sensitivity analyses were performed to address missing PRO and covariate data using multiple imputation by chained equations.^28^ A total of 100 imputed datasets were created, and the results from the linear mixed-effects models for each imputed dataset were combined using Rubin rules.^29^

Two-sided significance testing was used for all analyses. For analyses of the primary outcome, *P* < .046 (adjusted because of a prespecified interim analysis) was considered statistically significant. Results for secondary outcomes are reported as point estimates and 95% CIs. The confidence intervals for secondary outcomes were not adjusted for multiplicity.

The planned sample size of 640 participants was based on the primary objective of comparing the 12-week change in the BPI Interference score between the PCST and usual care groups. Assuming a standard deviation of change ranging from 2.0 to 3.0, an intrasite correlation coefficient of up to 0.02, a single interim efficacy/futility analysis of the primary outcome at 50% information time, and an attrition rate of 20%, the trial was anticipated to have 90% power at a 2-sided α level of .05 to detect between-group differences ranging from 0.62 to 1.17 points.

## Results

### Participants

**Figure 1.**
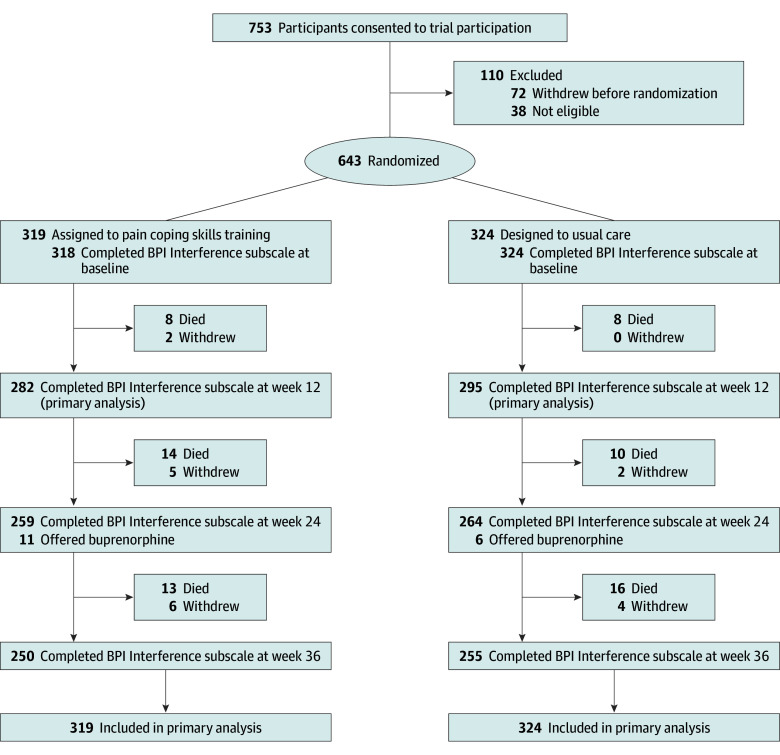
Flow Diagram of Participant Enrollment and Follow-Up Reasons for ineligibility among the 38 patients who consented but were subsequently found to be not eligible include hemodialysis for fewer than 90 days (n = 6); not meeting criteria for chronic pain (n = 5); Pain, Enjoyment of Life, and General Activity scale score lower than 4 (n = 3); current opioid or other nontobacco substance use disorder (n = 8); substantial cognitive impairment (n = 3); life expectancy shorter than 6 months (n = 2); unstable psychiatric disorder (n = 2); expected kidney transplant, dialysis unit transfer, or transfer to home dialysis (n = 3); not allowing access to opioid prescription data (n = 1), and other condition precluding participation (n = 5). BPI indicates Brief Pain Inventory.

Between January 4, 2021, and March 16, 2023, 753 patients receiving care at 103 dialysis facilities provided informed consent, and between January 22, 2021, and April 7, 2023, 643 of these individuals were randomized: 319 to PCST and 324 to usual care (Figure 1). The mean (SD) age of the participants was 60.3 (12.6) years; 288 (44.8%) were female; 22 (3.4%) self-identified as American Indian or Alaska Native, 6 (0.9%) as Asian, 308 (47.9%) as Black, 119 (18.5%) as Hispanic or Latino, 6 (0.9%) as Native Hawaiian or Other Pacific Islander, 210 (32.7%) as White, and 11 (1.7%) as multiracial; and 380 (59.2%) had diabetes (Table 1). The median (IQR) number of randomized participants per facility was 5 (3-8.25). Follow-up was completed on December 21, 2023.

**Table 1.** Baseline Characteristics

Characteristic	No. (%)		
	Overall (N = 643)	Pain coping skills training group (n = 319)	Usual care group (n = 324)
**Demographic characteristics**			
Age, mean (SD), y	60.3 (12.6)	60.3 (12.6)	60.4 (12.5)
Sex^a^			
Female	288 (44.8)	152 (47.6)	136 (42.0)
Male	355 (55.2)	167 (52.4)	188 (58.0)
Race and ethnicity^a^			
American Indian or Alaska Native	22 (3.4)	10 (3.1)	12 (3.7)
Asian	6 (0.9)	4 (1.3)	2 (0.6)
Black	308 (47.9)	160 (50.2)	148 (45.7)
Hispanic or Latino	119 (18.5)	62 (19.4)	57 (17.6)
Native Hawaiian or Other Pacific Islander	6 (0.9)	2 (0.6)	4 (1.2)
White	210 (32.7)	99 (31.0)	111 (34.3)
Multiracial	11 (1.7)	5 (1.6)	6 (1.9)
Preferred language Spanish	59 (9.2)	30 (9.4)	29 (9.0)
**Clinical characteristics**			
Postdialysis BMI, mean (SD)	31.3 (12.6)	30.9 (9.2)	31.7 (15.2)
Predialysis systolic BP, mean (SD), mm Hg	144.1 (25.4)	144.0 (26.8)	144.3 (23.9)
Predialysis diastolic BP, mean (SD), mm Hg	76.8 (15.7)	77.0 (16.3)	76.6 (15.2)
Duration of dialysis treatment, mean (SD), y	4.5 (4.7)	4.9 (5.2)	4.1 (4.1)
Cause of kidney failure			
Diabetic nephropathy	239 (37.2)	120 (37.6)	119 (36.7)
Hypertensive nephrosclerosis	118 (18.4)	62 (19.4)	56 (17.3)
Glomerular disease	24 (3.7)	13 (4.1)	11 (3.4)
Polycystic kidney disease	14 (2.2)	7 (2.2)	7 (2.2)
Acute kidney injury	17 (2.6)	10 (3.1)	7 (2.2)
Hereditary nephritis	8 (1.2)	4 (1.3)	4 (1.2)
Urinary tract disease or single kidney	7 (2.2)	3 (1.0)	4 (1.2)
Other	129 (20.0)	65 (20.4)	64 (19.8)
Unknown	87 (13.5)	35 (11.0)	52 (16.0)
Comorbidities			
Diabetes	380 (59.2)	187 (58.8)	193 (59.6)
Coronary artery disease	175 (27.8)	87 (27.6)	88 (27.9)
Heart failure	169 (26.6)	78 (24.7)	91 (28.5)
Atrial fibrillation or flutter	113 (18.5)	57 (18.6)	56 (18.4)
Ventricular arrhythmia	33 (5.5)	21 (7.0)	12 (4.0)
Stroke or transient ischemic attack	128 (20.0)	61 (19.3)	67 (20.7)
Peripheral vascular disease	102 (16.4)	59 (19.1)	43 (13.7)
Heart valve replacement or repair	35 (5.5)	20 (6.3)	15 (4.7)
Cancer	106 (16.5)	58 (18.2)	48 (14.9)
Current substance use			
Tobacco	99 (15.4)	46 (14.4)	53 (16.4)
Alcohol	64 (10.0)	30 (9.4)	34 (10.5)
Cannabis	102 (15.9)	53 (16.6)	49 (15.1)
Medications			
Opioid use during 3 of past 6 mo	138 (21.5)	71 (22.3)	67 (20.7)
Opioid use during past 14 d	162 (25.2)	81 (25.4)	81 (25.0)
Average MME/d, median (IQR)^b^	15.0 (5.0-33.8)	22.5 (7.0-30.0)	14.6 (5.0-33.9)
Type of pain^c^			
Musculoskeletal pain	567 (88.9)	280 (88.3)	287 (89.4)
Neuropathic pain	423 (66.3)	218 (68.8)	205 (63.9)
Hemodialysis vascular access pain	173 (27.1)	87 (27.4)	86 (26.8)
Other vascular pain	94 (14.7)	48 (15.1)	46 (14.3)
Visceral pain	158 (24.8)	83 (26.2)	75 (23.4)
Chronic headache	151 (23.7)	81 (25.6)	70 (21.8)
Cancer pain	8 (1.3)	5 (1.6)	3 (0.9)
**Laboratory values**			
Blood urea nitrogen, mean (SD), mg/dL	54.0 (22.5)	53.3 (22.5)	54.7 (22.5)
Creatinine, mean (SD), mg/dL	9.3 (3.9)	9.4 (4.7)	9.1 (3.0)
Albumin, mean (SD), g/dL	3.9 (0.4)	3.9 (0.4)	3.9 (0.4)
Hemoglobin, mean (SD), g/dL	10.9 (1.7)	10.8 (1.9)	10.9 (1.4)
Bicarbonate, mean (SD), mEq/L	24.3 (4.1)	24.5 (4.1)	24.1 (4.1)
Single-pool urea, Kt/V^d^	1.6 (0.3)	1.6 (0.3)	1.5 (0.3)
**Baseline PROs, mean (SD)**			
BPI Interference	6.4 (2.1)	6.3 (2.0)	6.5 (2.1)
BPI Severity	5.9 (2.1)	5.8 (2.1)	6.0 (2.1)
PCS SF-6	15.5 (5.6)	15.5 (5.6)	15.5 (5.7)
Single-item QOL	5.9 (2.6)	6.1 (2.6)	5.8 (2.7)
PHQ-9	9.2 (5.9)	8.8 (5.8)	9.5 (6.1)
GAD-7	7.1 (6.0)	6.8 (5.8)	7.4 (6.1)

Abbreviations: BMI, body mass index (calculated as weight in kilograms divided by height in meters squared); BP, blood pressure; BPI, Brief Pain Inventory; GAD-7, 7-item generalized anxiety disorder; MME, morphine milligram equivalents; PCS SF-6, Pain Catastrophizing Scale 6-item short form; PHQ-9, 9-item Patient Health Questionnaire for depression; PRO, patient-reported outcome; QOL, quality of life.

SI conversion factors: To convert urea nitrogen to millimoles per liter, multiply by 0.357; creatinine to micromoles per liter, multiply by 88.4; albumin and hemoglobin to grams per liter, multiply by 10; bicarbonate to millimoles per liter, multiply by 1.

^a^ Sex and race and ethnicity were based on self-report by the participant.

^b^ Determined for those with opioid use during the past 14 days (117 overall; 53 in the pain coping skills training group; 64 in the usual care group).

^c^ A participant could report multiple types of pain.

^d^ The single-pool urea Kt/V is a dimensionless indicator of removal of small molecules during a single dialysis treatment. K represents the urea clearance by the dialyzer, t represents the treatment time, and V represents the volume of distribution of urea.

### Intervention Adherence and Fidelity, Visit Completeness, and Trial Retention

The median (IQR) number of coach-led PCST sessions attended by the 319 participants randomized to PCST was 12.0 (10.0-12.0) out of a maximum of 12 (eTable 4 in Supplement 3); 2063 of 3333 sessions (61.9%) were conducted by video conferencing, and 2106 of 3333 (63.2%) took place during dialysis (eTable 5 in Supplement 3). Ten participants (3.1%) withdrew from the coach-led portion of PCST but continued participation in the trial. Of the 21 034 daily automated telephone contacts initiated during the 12-week IVR intervention period, 11 206 (53.3%) were accepted by the participants, and the per-participant median (IQR) completion was 59.8% (19.1%-86.9%). An independent review of 334 recordings (10.0%) of the coach-led PCST sessions found that 310 (92.8%) sessions met the prespecified criteria for successful completion of required components. Withdrawal from the trial for reasons other than death occurred among 13 participants (4.1%) in the PCST group and 6 (1.9%) in the usual care group.

### Primary Outcome

**Figure 2.**
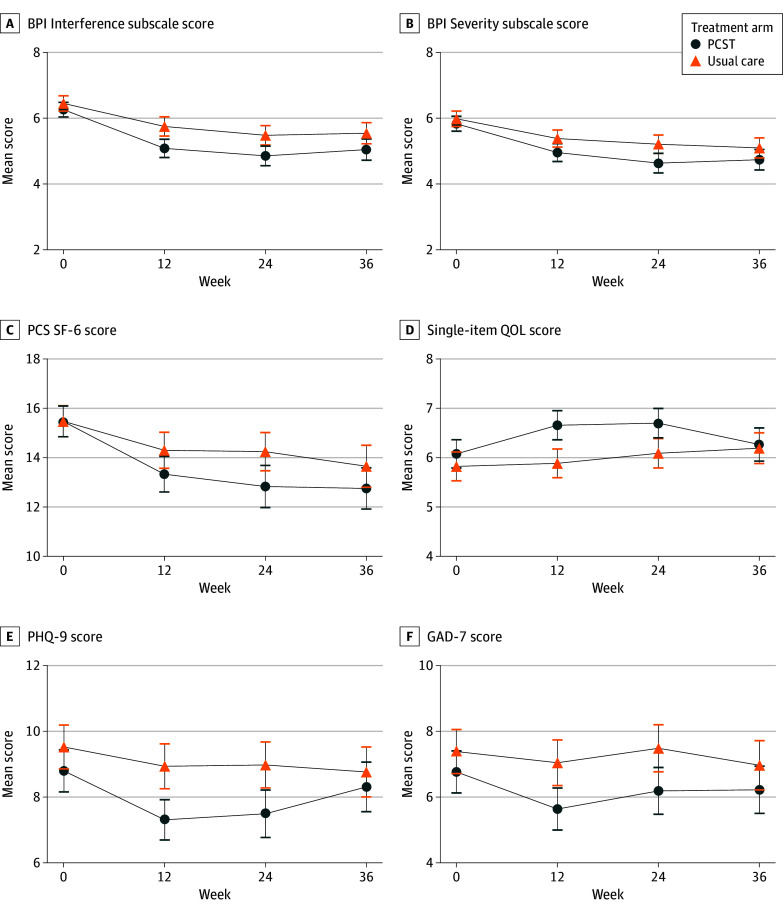
Effects of Pain Coping Skills Training (PCST) on Primary and Secondary Patient-Reported Outcomes For quality of life (QOL), a higher score indicates better outcome; for all other measures, a higher score indicates worse outcome. Error bars represent 95% CIs. BPI indicates Brief Pain Inventory; GAD-7, 7-item generalized anxiety disorder; PCS SF-6, Pain Catastrophizing Scale 6-item short form; PHQ-9, 9-item Patient Health Questionnaire for depression.

As shown in Table 2 and Figure 2, there was a greater reduction in pain interference in the PCST group compared with the usual care group, with a between-group difference in the BPI Interference score of −0.49 (95% CI, −0.85 to −0.12; *P* = .009) at week 12 (primary analysis). The effect was preserved at week 24 (between-group difference in BPI Interference score, −0.48; 95% CI, −0.86 to −0.11) but was diminished at week 36 (between-group difference in BPI Interference score, −0.34; 95% CI, −0.72 to 0.04). Results were similar in sensitivity analyses using multiple imputation for missing outcomes (eTable 6 in Supplement 3) and when incorporating the dialysis facility rather than the enrolling site as a random effect in the linear models (eTable 7 in Supplement 3).

**Table 2.** Effect of Pain Coping Skills Training on Brief Pain Inventory (BPI) Interference Score^a^

Follow-up time point	Mean (SD) [No. of participants]				Between-group difference, mean (95% CI)^c^
	Pain coping skills training group		Usual care group		
	Follow-up	Change from baseline^b^	Follow-up	Change from baseline^b^	
Week 12^d^	5.09 (2.37) [282]	−1.19 (2.38) [281]	5.75 (2.55) [295]	−0.71 (2.23) [295]	−0.49 (−0.85 to −0.12)^e^
Week 24	4.86 (2.44) [259]	−1.43 (2.22) [258]	5.48 (2.43) [264]	−0.90 (2.18) [264]	−0.48 (−0.86 to −0.11)
Week 36	5.05 (2.60) [250]	−1.20 (2.39) [249]	5.55 (2.61) [255]	−0.81 (2.36) [255]	−0.34 (−0.72 to 0.04)
**Decrease in BPI interference score >1 point**					
**Follow-up time point**	**No./total No. of participants (%)**				**Odds ratio (95% CI)**
Week 12	143/281 (50.9)		108/295 (36.6)		1.79 (1.28 to 2.49)
Week 24	142/258 (55.0)		113/264 (42.8)		1.59 (1.13 to 2.24)
Week 36	120/249 (48.2)		108/255 (42.4)		1.26 (0.89 to 1.79)

^a^ Range of 0 to 10, with higher score indicating more pain interference. Usual care is the reference group.

^b^ See Table 1 for baseline scores.

^c^ Between-group differences were calculated using a linear mixed-effects model with random effects of enrolling site (stratification factor) and participants within each enrolling site, and fixed effects of intervention, time, interactions between intervention and time, and baseline opioid use (stratification factor). All other summary statistics are crude values.

^d^ Time point for the primary analysis.

^e^ *P* = .009.

A larger proportion of participants in the PCST group, compared with the usual care group, had a decrease in BPI Interference score greater than the 1-point minimal clinically important difference at week 12 (143 of 281 [50.9%] vs 108 of 295 [36.6%]; odds ratio [OR], 1.79; 95% CI, 1.28-2.49) and at week 24 (142 of 258 [55.0%] vs 113 of 264 [42.8%]; OR, 1.59; 95% CI, 1.13-2.24) but not at week 36 (120 of 249 [48.2%] vs 108 of 255 [42.4%]; OR, 1.26; 95% CI, 0.89-1.79) (Table 2). Using an alternative threshold of a more than 30% reduction from baseline in the BPI Interference score (a threshold considered moderate in magnitude for the BPI Intensity subscale^26^) in a post hoc sensitivity analysis, between-group differences favoring PCST were evident at weeks 12, 24, and 36 (eTable 8 in Supplement 3). There was not evidence for effect modification by demographic characteristics or opioid use at baseline (eFigure in Supplement 3).

### Secondary Outcomes

Changes in the prespecified subset of secondary PROs are shown in Table 3 and Figure 2. At week 12, changes from baseline in the single-item quality of life and 7-item generalized anxiety disorder scores favored PCST, with between-group differences of 0.52 (95% CI, 0.04-1.00) and −0.88 (95% CI, −1.70 to −0.05), respectively. At week 24, changes from baseline in the BPI Severity, Pain Catastrophizing Scale 6-item short form, 7-item generalized anxiety disorder, and 9-item Patient Health Questionnaire for depression scores also favored PCST. At week 36, the effect appeared to persist for pain catastrophizing but not for the other outcomes. For the composite outcome of pain interference and opioid use in the PCST and usual care groups, 127 of 252 participants (50.4%) and 112 of 283 participants (39.6%), respectively, attained the success outcome at 12 weeks (OR, 1.50; 95% CI, 1.07-2.11). An effect was not evident at weeks 24 or 36 (eTable 9 in Supplement 3). The clinical outcomes of falls, hospitalizations, and deaths occurred at high rates, as expected for this patient population, and were similar between the groups (eTable 10 in Supplement 3).

**Table 3.** Effect of Pain Coping Skills Training on Secondary Patient-Reported Outcomes

Patient-reported outcome^a^	Mean (SD) [No. of participants]				Between-group difference, mean (95% CI)^c^
	Pain coping skills training group		Usual care group		
	Follow-up	Change from baseline^b^	Follow-up	Change from baseline^b^	
**Week 12**					
Pain intensity: BPI Severity (range, 0-10)	4.95 (2.32) [282]	−0.86 (2.09) [281]	5.38 (2.24) [290]	−0.57 (1.87) [289]	−0.27 (−0.61 to 0.06)
Catastrophizing: PCS SF-6 (range, 0-24)	13.34 (6.14) [282]	−1.90 (6.26) [279]	14.30 (6.32) [291]	−1.10 (5.70) [286]	−0.93 (−1.86 to 0.01)
QOL: Single-item QOL (range, 0-10)	6.66 (2.53) [282]	0.55 (2.88) [282]	5.88 (2.54) [291]	0.04 (2.97) [291]	0.52 (0.04 to 1.00)
Depression: PHQ-9 (range, 0-27)	7.29 (5.22) [277]	−1.41 (5.13) [269]	8.92 (5.83) [278]	−0.52 (5.08) [273]	−0.86 (−1.72 to −0.01)
Anxiety: GAD-7 (range, 0-21)	5.63 (5.48) [281]	−1.23 (4.94) [281]	7.04 (6.03) [288]	−0.34 (5.08) [286]	−0.88 (−1.70 to −0.05)
MME/d^d^	47 (163) [43]	1 (18) [32]	32 (53) [55]	5 (49) [38]	−4 (−18 to 10)
**Week 24**					
Pain intensity: BPI Severity (range, 0-10)	4.64 (2.44) [258]	−1.24 (2.17) [257]	5.21 (2.31) [263]	−0.65 (2.09) [262]	−0.50 (−0.85 to −0.16)
Catastrophizing: PCS SF-6 (range, 0-24)	12.84 (6.93) [257]	−2.50 (6.24) [254]	14.24 (6.37) [263]	−0.96 (5.52) [259]	−1.49 (−2.46 to −0.52)
QOL: Single-item QOL (range, 0-10)	6.70 (2.43) [256]	0.61 (2.80) [256]	6.09 (2.46) [263]	0.21 (3.22) [263]	0.40 (−0.09 to 0.89)
Depression: PHQ-9 (range, 0-27)	7.47 (5.90) [255]	−1.44 (5.54) [246]	8.96 (5.72) [255]	−0.23 (5.37) [253]	−0.94 (−1.82 to −0.06)
Anxiety: GAD-7 (range, 0-21)	6.18 (5.83) [257]	−0.79 (5.26) [257]	7.48 (5.89) [259]	0.41 (5.08) [257]	−0.98 (−1.84 to −0.13)
MME/d^d^	48 (165) [43]	6 (36) [27]	29 (44) [41]	3 (17) [28]	2 (−13 to 17)
**Week 36**					
Pain intensity: BPI Severity (range, 0-10)	4.74 (2.49) [248]	−1.07 (2.33) [247]	5.10 (2.46) [254]	−0.72 (2.26) [253]	−0.28 (−0.63 to 0.06)
Catastrophizing: PCS SF-6 (range, 0-24)	12.76 (6.68) [249]	−2.69 (6.13) [246]	13.66 (6.85) [253]	−1.44 (6.27) [248]	−1.12 (−2.10 to −0.14)
QOL: Single-item QOL (range, 0-10)	6.27 (2.70) [249]	0.18 (3.28) [249]	6.19 (2.52) [252]	0.16 (3.09) [252]	−0.10 (−0.60 to 0.40)
Depression: PHQ-9 (range, 0-27)	8.29 (6.04) [245]	−0.58 (5.48) [236]	8.75 (6.09) [245]	−0.45 (6.06) [242]	−0.01 (−0.90 to 0.88)
Anxiety: GAD-7 (range, 0-21)	6.22 (5.75) [245]	−0.80 (5.47) [245]	6.96 (6.09) [253]	−0.10 (5.95) [252]	−0.55 (−1.42 to 0.31)
MME/d^d^	49 (173) [39]	−0 (10) [21]	27 (43) [45]	1 (28) [26]	−2 (−18 to 13)

Abbreviations: BPI, Brief Pain Inventory; GAD-7, 7-item generalized anxiety disorder; MME, morphine milligram equivalents; PCS SF-6, Pain Catastrophizing Scale 6-item short form; PHQ-9, 9-item Patient Health Questionnaire for depression; QOL, quality of life.

^a^ For QOL, a higher score indicates better outcome; for all other measures, a higher score indicates worse outcome.

^b^ See Table 1 for baseline scores.

^c^ Between-group differences were calculated using a linear mixed-effects model with random effects of enrolling site (stratification factor) and participants within each enrolling site, and fixed effects of intervention, time, interactions between intervention and time, and baseline opioid use (stratification factor). All other summary statistics are crude values.

^d^ Includes participants reporting opioid use during the previous 14 days. Mean (SD) MME/d at baseline was 45.0 (147.6) for the pain coping skills training group and 24.2 (36.3) for the usual care group. The skewed nature of the distribution reduces the validity of the mixed-effects model.

### Adverse Events

For serious adverse events and adverse events of interest, neither the proportion of participants with an event nor the event rates differed between the PCST and usual care groups (eTable 11 in Supplement 3).

## Discussion

In this multicenter randomized clinical trial that enrolled patients undergoing maintenance hemodialysis who had moderate or severe chronic pain, PCST produced a statistically significant decrease in pain interference compared with usual care. The effect on the overall cohort was modest, but the intervention produced a clinically important change for a substantial proportion of participants. The benefit was evident at the end of the initial 12-week period of coach-led sessions (primary analysis) and at the end of the subsequent 12-week period of IVR sessions (week 24). At week 36, 12 weeks after the full PCST intervention ended, the magnitude of the effect on pain interference was diminished. Benefits of the intervention were also apparent for the secondary PROs of pain severity, quality of life, depression, and anxiety at week 12 and/or week 24, and, for pain catastrophizing, at weeks 24 and 36.

While symptoms, including pain, are highly prioritized as targets for interventions by patients with kidney failure, improvements in symptoms or quality of life have only rarely been demonstrated among this patient population.^2,4,30,31,32^ An apparent benefit of PCST on pain, anxiety, and depression—conditions that often coexist—suggests that the intervention may have an impact across multiple domains.^3,33^ Nonpharmacologic approaches are appealing given the high burden of medications required for this patient population and the limited options for analgesic medications in the setting of kidney failure.^34,35,36^ The absence of adverse effects attributable to PCST contrasts favorably with pharmacologic pain interventions.^5,37^

The PCST intervention was adapted for this trial to address the high comorbidity and treatment burden that characterizes dialysis-dependent kidney disease; however, the overall principles and structure of the coach-led component was based on well-established programs and, therefore, the primary analysis of efficacy was based on the outcome measures assessed at the conclusion of the coach-led component rather than at the end of the less well-studied IVR component.^17,18,38^ The finding that the benefit of PCST persisted until week 24 supports further evaluation of IVR for maintaining, and potentially supplementing, the effects of coach-led PCST. Centralized delivery of PCST, as was used successfully for the trial across a large number of dialysis facilities, provides an approach for efficiently implementing the intervention by dialysis provider organizations outside of the trial setting.

The observed effect of the PCST intervention may be interpreted as modest because the between-group difference of approximately 0.5 points in the BPI Interference score is smaller than the minimal clinically important difference of 1 point previously established at the level of the individual person. However, as stated in expert consensus recommendations by IMMPACT,^26,39^ and by others, thresholds for clinically important changes in individuals should not be directly applied to differences between groups. For between-group comparisons, smaller differences in changes from baseline can be interpreted as meaningful and need to be considered in the broader context of the underlying condition, available treatments, risks vs benefits, and the proportion of individuals meeting criteria for a clinically important change.^39^ The finding in this trial that a greater proportion of participants in the intervention group compared with the usual care group had a higher than 1-point reduction in the BPI Interference score (absolute difference of 14%, relative difference of 39%, and number needed to treat of 7) suggests that PCST had a meaningful effect. The results are particularly compelling when considering the limited treatment options for pain in the setting of kidney failure; the high acceptability, tolerability, and safety of the intervention; and the observed effects on pain, anxiety, depression, and quality of life—all outcomes that have only rarely been shown to improve in this patient population.^3^ Additionally, despite the high comorbidity burden associated with kidney failure, which likely diminishes a response to any intervention, the effect of PCST on pain interference observed in this trial was similar in magnitude to effects reported in systematic reviews of widely used nonpharmacologic pain therapies among populations with substantially lower disease severity.^39,40^

We observed nominal improvements in the usual care group for the primary outcome and several of the secondary outcomes. These improvements may have been due to the regular engagement with the research teams that was built into the protocol for both randomized groups to ensure standardized collection of follow-up data. This finding illustrates the importance, for distinguishing effects of an intervention from effects of trial participation, of including a comparator group that does not receive an active treatment.

### Strengths and Limitations

This trial has several strengths. The cohort was demographically diverse, with participants enrolled from 16 centers and 103 dialysis facilities in multiple geographic regions and in urban and rural settings. The proportions of female and Black and Hispanic or Latino participants matched or exceeded those of the overall US dialysis patient population.^40^ The intervention was administered using a standardized approach with a high degree of fidelity and was available to both Spanish- and English-speaking participants. Adherence to the intervention was high, suggesting acceptability of the intervention despite the burden of thrice-weekly hemodialysis treatments and frequent hospitalizations. The trial was designed and implemented with input at all stages from a highly engaged group of patients with kidney failure.^11^

The trial also has limitations. Participants and members of the site research teams were not masked to treatment assignments; however, the PROs were ascertained centrally by individuals who were not aware of group assignments and who were otherwise not involved in trial activities. Ascertainment of the PROs was not performed for approximately 10% of participants at week 12 and approximately 20% of participants at later time points, in large part because of participant death. This level of attrition was anticipated and incorporated into the sample size determination. Importantly, sensitivity analyses with multiple imputation to address missing outcomes yielded results similar to those of the primary analyses. We did not incorporate a cost-effectiveness analysis into the trial and, therefore, cannot assess the economic implications of adopting the intervention. Evaluating both costs of the intervention and potential cost savings associated with improved outcomes is an important area for future work.

## Conclusions

This randomized clinical trial of PCST showed benefits on pain interference, quality of life, and several other PROs among individuals undergoing maintenance hemodialysis and experiencing chronic pain. While the effect on the overall cohort was of modest magnitude, the intervention resulted in a clinically meaningful improvement in pain interference for a substantial proportion of participants. Centrally administered PCST may provide a low-risk, scalable approach for people with dialysis-dependent kidney failure, a population with limited options for managing pain.

## Notes

Supplement 1. Trial Protocol

Supplement 2. Statistical Analysis Plan

Supplement 3. **eAppendix.** HOPE Consortium, Cores, and Data and Safety Monitoring Board Members **eTable 1.** Eligibility Criteria **eTable 2.** Training, Certification, Supervision, and Fidelity Monitoring of PCST Coaches **eTable 3.** Complete List of Outcome Measures **eTable 4.** Adherence to Coach-Led PCST Sessions Among Participants Randomized to PCST Group **eTable 5.** Delivery Method and Location for Coach-Led PCST Sessions **eTable 6.** Sensitivity Analysis: Effect of Pain Coping Skills Training on Patient-Reported Outcomes Using Multiple Imputation to Address Missing Outcomes **eTable 7.** Sensitivity Analysis: Comparison of Models with Enrolling Site versus Dialysis Facility as Random Effect (enrolling site was used in primary analysis) **eTable 8.** Sensitivity Analysis: Decrease of >30% in BPI Interference Score **eTable 9. **Composite Outcome of Pain Interference and Opioid Use **eTable 10.** Clinical Event Outcomes **eTable 11.** Adverse Events **eFigure.** Subgroup Analysis of the Primary Endpoint: Change in BPI Interference between Baseline and Week 12

Supplement 4. **Nonauthor Collaborators.** Members of the HOPE Consortium

Supplement 5. Data Sharing Statement

## References

[1] Abdel-Kader K, Unruh ML, Weisbord SD. Symptom burden, depression, and quality of life in chronic and end-stage kidney disease. Clin J Am Soc Nephrol. 2009;4(6):1057-1064. doi:10.2215/CJN.00430109 19423570 PMC2689883

[2] Fletcher BR, Damery S, Aiyegbusi OL, . Symptom burden and health-related quality of life in chronic kidney disease: a global systematic review and meta-analysis. PLoS Med. 2022;19(4):e1003954. doi:10.1371/journal.pmed.1003954 35385471 PMC8985967

[3] Mehrotra R, Davison SN, Farrington K, ; Conference Participants. Managing the symptom burden associated with maintenance dialysis: conclusions from a Kidney Disease: Improving Global Outcomes (KDIGO) Controversies Conference. Kidney Int. 2023;104(3):441-454. doi:10.1016/j.kint.2023.05.019 37290600

[4] Urquhart-Secord R, Craig JC, Hemmelgarn B, . Patient and caregiver priorities for outcomes in hemodialysis: an international nominal group technique study. Am J Kidney Dis. 2016;68(3):444-454. doi:10.1053/j.ajkd.2016.02.037 26968042

[5] Roy PJ, Weltman M, Dember LM, Liebschutz J, Jhamb M; HOPE Consortium. Pain management in patients with chronic kidney disease and end-stage kidney disease. Curr Opin Nephrol Hypertens. 2020;29(6):671-680. doi:10.1097/MNH.0000000000000646 32941189 PMC7753951

[6] Brintz CE, Cheatle MD, Dember LM, ; HOPE Consortium. Nonpharmacologic treatments for opioid reduction in patients with advanced chronic kidney disease. Semin Nephrol. 2021;41(1):68-81. doi:10.1016/j.semnephrol.2021.02.007 33896475 PMC9366727

[7] Jhamb M, Steel JL, Yabes JG, . Effects of technology assisted stepped collaborative care intervention to improve symptoms in patients undergoing hemodialysis: the TĀCcare randomized clinical trial. JAMA Intern Med. 2023;183(8):795-805. doi:10.1001/jamainternmed.2023.2215 37338898 PMC10282960

[8] Keefe FJ, Caldwell DS, Williams DA, . Pain coping skills training in the management of osteoarthritic knee pain: a comparative study. Behav Ther. 1990;21(1):49-62. doi:10.1016/S0005-7894(05)80188-1

[9] Keefe FJ, Caldwell DS, Williams DA, . Pain coping skills training in the management of osteoarthritic knee pain-II: follow-up results. Behav Ther. 1990;21(4):435-447. doi:10.1016/S0005-7894(05)80357-0

[10] Keefe FJ. Cognitive behavioral therapy for managing pain. Clin Psychol. 1996;49:4-5.

[11] Dember LM, Hsu JY, Bernardo L, ; HOPE Consortium. The design and baseline characteristics for the HOPE Consortium Trial to reduce pain and opioid use in hemodialysis. Contemp Clin Trials. 2024;136:107409. doi:10.1016/j.cct.2023.107409 38086444 PMC10922728

[12] Malinoff HL, Barkin RL, Wilson G. Sublingual buprenorphine is effective in the treatment of chronic pain syndrome. Am J Ther. 2005;12(5):379-384. doi:10.1097/01.mjt.0000160935.62883.ff 16148422

[13] Daitch D, Daitch J, Novinson D, Frey M, Mitnick C, Pergolizzi J Jr. Conversion from high-dose full-opioid agonists to sublingual buprenorphine reduces pain scores and improves quality of life for chronic pain patients. Pain Med. 2014;15(12):2087-2094. doi:10.1111/pme.12520 25220043

[14] Helping to End Addiction Long-term Initiative. National Institutes of Health. Accessed February 22, 2024. https://heal.nih.gov/

[15] Baker RG, Koroshetz WJ, Volkow ND. The Helping to End Addiction Long-term (HEAL) Initiative of the National Institutes of Health. JAMA. 2021;326(11):1005-1006. doi:10.1001/jama.2021.13300 34436519

[16] Krebs EE, Lorenz KA, Bair MJ, . Development and initial validation of the PEG, a three-item scale assessing pain intensity and interference. J Gen Intern Med. 2009;24(6):733-738. doi:10.1007/s11606-009-0981-1 19418100 PMC2686775

[17] Heapy AA, Higgins DM, Goulet JL, . Interactive voice response-based self-management for chronic back pain: the COPES noninferiority randomized trial. JAMA Intern Med. 2017;177(6):765-773. doi:10.1001/jamainternmed.2017.0223 28384682 PMC5818820

[18] Piette JD, Newman S, Krein SL, . Patient-centered pain care using artificial intelligence and mobile health tools: a randomized comparative effectiveness trial. JAMA Intern Med. 2022;182(9):975-983. doi:10.1001/jamainternmed.2022.3178 35939288 PMC9361183

[19] Cleeland CS, Ryan KM. Pain assessment: global use of the Brief Pain Inventory. Ann Acad Med Singap. 1994;23(2):129-138. 8080219

[20] Sullivan MJLBS, Pivik J. The Pain Catastrophizing Scale: development and validation. Psychol Assess. 1995;7:524-532. doi:10.1037/1040-3590.7.4.524

[21] Cohen SR, Sawatzky R, Russell LB, Shahidi J, Heyland DK, Gadermann AM. Measuring the quality of life of people at the end of life: the McGill Quality of Life Questionnaire-Revised. Palliat Med. 2017;31(2):120-129. doi:10.1177/0269216316659603 27412257

[22] Spitzer RL, Kroenke K, Williams JB; Patient Health Questionnaire Primary Care Study Group. Validation and utility of a self-report version of PRIME-MD: the PHQ primary care study. JAMA. 1999;282(18):1737-1744. doi:10.1001/jama.282.18.1737 10568646

[23] Spitzer RL, Kroenke K, Williams JB, Löwe B. A brief measure for assessing generalized anxiety disorder: the GAD-7. Arch Intern Med. 2006;166(10):1092-1097. doi:10.1001/archinte.166.10.1092 16717171

[24] Sobell LC, Brown J, Leo GI, Sobell MB. The reliability of the Alcohol Timeline Followback when administered by telephone and by computer. Drug Alcohol Depend. 1996;42(1):49-54. doi:10.1016/0376-8716(96)01263-X 8889403

[25] Laird NM, Ware JH. Random-effects models for longitudinal data. Biometrics. 1982;38(4):963-974. doi:10.2307/2529876 7168798

[26] Dworkin RH, Turk DC, Wyrwich KW, . Interpreting the clinical importance of treatment outcomes in chronic pain clinical trials: IMMPACT recommendations. J Pain. 2008;9(2):105-121. doi:10.1016/j.jpain.2007.09.005 18055266

[27] Fitzmaurice G, Laird N, Ware J. Applied Longitudinal Analysis. 2nd ed. John Wiley & Sons, Inc; 2011. doi:10.1002/9781119513469

[28] Van Buuren S, Brand JPL, Groothuis-Oudshoorn CGM, Rubin DB. Fully conditional specification in multivariate imputation. J Stat Comput Simul. 2006;76(12):1049-1064. doi:10.1080/10629360600810434

[29] Rubin DB. Multiple Imputation for Nonresponse in Surveys. John Wiley and Sons; 1987. doi:10.1002/9780470316696

[30] Flythe JE, Hilliard T, Castillo G, . Symptom prioritization among adults receiving in-center hemodialysis: a mixed methods study. Clin J Am Soc Nephrol. 2018;13(5):735-745. doi:10.2215/CJN.10850917 29559445 PMC5969481

[31] Weisbord SD, Fried LF, Arnold RM, . Prevalence, severity, and importance of physical and emotional symptoms in chronic hemodialysis patients. J Am Soc Nephrol. 2005;16(8):2487-2494. doi:10.1681/ASN.2005020157 15975996

[32] Claxton RN, Blackhall L, Weisbord SD, Holley JL. Undertreatment of symptoms in patients on maintenance hemodialysis. J Pain Symptom Manage. 2010;39(2):211-218. doi:10.1016/j.jpainsymman.2009.07.003 19963337

[33] Jhamb M, Abdel-Kader K, Yabes J, . Comparison of fatigue, pain, and depression in patients with advanced kidney disease and cancer-symptom burden and clusters. J Pain Symptom Manage. 2019;57(3):566-575. doi:10.1016/j.jpainsymman.2018.12.006 30552961 PMC6382584

[34] Manley HJ, Garvin CG, Drayer DK, . Medication prescribing patterns in ambulatory haemodialysis patients: comparisons of USRDS to a large not-for-profit dialysis provider. Nephrol Dial Transplant. 2004;19(7):1842-1848. doi:10.1093/ndt/gfh280 15128886

[35] Chiu YW, Teitelbaum I, Misra M, de Leon EM, Adzize T, Mehrotra R. Pill burden, adherence, hyperphosphatemia, and quality of life in maintenance dialysis patients. Clin J Am Soc Nephrol. 2009;4(6):1089-1096. doi:10.2215/CJN.00290109 19423571 PMC2689877

[36] Schmid H, Hartmann B, Schiffl H. Adherence to prescribed oral medication in adult patients undergoing chronic hemodialysis: a critical review of the literature. Eur J Med Res. 2009;14(5):185-190. doi:10.1186/2047-783X-14-5-185 19541573 PMC3351975

[37] Busse JW, Wang L, Kamaleldin M, . Opioids for chronic noncancer pain: a systematic review and meta-analysis. JAMA. 2018;320(23):2448-2460. doi:10.1001/jama.2018.18472 30561481 PMC6583638

[38] Naylor MR, Naud S, Keefe FJ, Helzer JE. Therapeutic Interactive Voice Response (TIVR) to reduce analgesic medication use for chronic pain management. J Pain. 2010;11(12):1410-1419. doi:10.1016/j.jpain.2010.03.019 20620119 PMC3045626

[39] Smith SM, Dworkin RH, Turk DC, . Interpretation of chronic pain clinical trial outcomes: IMMPACT recommended considerations. Pain. 2020;161(11):2446-2461. doi:10.1097/j.pain.0000000000001952 32520773 PMC7572524

[40] 2021 USRDS annual data report. United States Renal Data System. Accessed November 20, 2024. https://usrds-adr.niddk.nih.gov/2021

