# Supplementary material for: Pain Coping Skills Training for Patients Receiving Hemodialysis: The HOPE Consortium Randomized Clinical Trial
Source: JAMA Intern Med. 2024 Dec 30;185(2):197–207. doi: 10.1001/jamainternmed.2024.7140 (PMC11791705; doi:10.1001/jamainternmed.2024.7140)
Supplement: Supplement 1. — Trial Protocol [file jamainternmed-e247140-s001.pdf]

FINAL PROTOCOL

Changes to Original Protocol are listed on pages 75-79

HOPE Consortium Trial to Reduce Pain and Opioid Use in Hemodialysis

A Randomized Clinical Trial to Evaluate Non-Pharmacologic and Pharmacologic Approaches for Reducing Pain and Opioid Use Among Patients Treated with Maintenance Hemodialysis

A National Institutes of Health HEAL Initiative Trial

**Funding Sponsor:**	*National Institutes of Diabetes and Digestive and Kidney Diseases*<br>*National Institutes of Health*<br>*2 Democracy Plaza*<br>*6707 Democracy Boulevard*<br>*Bethesda, MD 20892-5458*
**Study Product:**	Buprenorphine
**Protocol Number:**	843471
**IND Number:**	151345
**Protocol Version:**	V1.7; July 7, 2023

Table of Contents

PRINCIPAL INVESTIGATOR SIGNATURE **6**
1. Study Summary **8**
1.1. Synopsis 8
1.2. Key Roles and Study Governance 10
1.3. Study Schema 11
2. Introduction and Rationale **12**
2.1. Study Rationale 12
2.2. Background 12
2.2.1. Prevalence of Pain among Patients Treated with Hemodialysis 12
2.2.2. Complexity of Pain Management among Patients Treated with Hemodialysis 13
2.2.3. Potential Harms from Long Term Opioid Therapy 14
2.2.4. The Need for Non-Pharmacologic Approaches to Pain Management and Opioid Reduction in the Setting of Maintenance Hemodialysis 14
2.2.5. Rationale for a Pain Coping Skills Training Intervention to Address Pain and Opioid Use 14
2.2.6. Rationale for a Buprenorphine Intervention to Address Opioid Physical Dependence in Hemodialysis Patients 16
2.3. Risk / Benefit Assessment for the HOPE Trial Interventions 17
2.3.1. Known Potential Risks of Trial Interventions 17
2.3.2. Known Potential Benefits of Trial Interventions 18
2.3.3. Assessment of the Balance between Risks and Benefits 18
3. Study Objectives **19**
4. Study Design and Outcomes **20**
4.1. Trial Design and Rationale 20
4.2. Outcomes 20
4.2.1. Primary Outcome: Pain Interference 21
4.2.2. Secondary Outcomes 21
4.2.3. Patient-Reported Outcome (PRO) Ascertainment Approach 23
4.2.4. Timing of Ascertainment of Primary Outcome 24
5. Study Population **25**
5.1. Inclusion Criteria 25
5.2. Exclusion Criteria 25
5.3. Subgroup with Current or Recent Opioid Use 25
5.4. Participant Recruitment and Eligibility Screening 26
5.5. Re-Screening for Eligibility 26
6. Study Interventions **27**
6.1. Pain Coping Skills Training Intervention (PCST) 27
6.1.1. Rationale for Using Telehealth and IVR as Delivery Modes for PCST 28
6.1.2. Telehealth Implementation 28
6.1.3. IVR Implementation 29
6.1.4. Structure and Content of PCST Telehealth Intervention (Weeks 1 - 12) 29
6.1.5. Structure and Content of IVR Booster (Weeks 13-24) 30
6.1.6. Qualifications and Training of the PCST Coaches 32
6.2. Buprenorphine Intervention 32

6.2.1. Overview 32
6.2.2. Eligibility for Buprenorphine Intervention: Inclusion Criteria 32
6.2.3. Eligibility for Buprenorphine Intervention: Exclusion Criteria 33
6.2.4. Buprenorphine Formulations 33
6.2.5. Buprenorphine Regulatory Issues 34
6.2.6. Buprenorphine Prescribing and Dispensing 35
6.2.7. Buprenorphine Initiation 35
6.2.8. Buprenorphine Dosing Regimens 37
6.2.9. Buprenorphine Maintenance During and After the Trial 38
6.2.10. Monitoring for Changes in Concomitant Medications During Treatment with Buprenorphine with Particular Attention to Medications that Affect Cyp3A4 Enzyme Activity 38
6.3. Usual Care 38
6.4. Intervention Fidelity 39
6.5. Clinical Management of Trial Participants 39
6.5.1. Concomitant Therapy 39
6.5.2. Opioid Adherence Problems 40
6.5.3. Clinical Management of OUD or other Substance Use Disorders 40
6.5.4. Clinical Management of Suicidality 40
7. Study Procedures and Assessments 42
7.1. Pre-screening and Screening 42
7.2. Baseline Data Collection and PRO Ascertainment – (within 4 weeks of Screening) 42
7.3. Randomization for Phase 1 (within 1 week of Baseline) 43
7.4. Phase 1 Intervention Administration, Data Collection, and Research Team Contacts (Weeks 1 - 24) 44
7.4.1. Intervention Administration (Weeks 1 - 24) 44
7.4.2. Contacts with the Research Team (Weeks 4, 8, 12, 16, and 20) 44
7.4.3. Follow-Up PROs (Week 12) 44
7.4.4. End of Phase 1 Intervention, Week 24 PROs, and Determination of Eligibility for Buprenorphine (Week 24) 45
7.5. Phase 2 Intervention Administration, Data Collection, and Research Team Contacts (Weeks 24 - 36) 46
7.5.1. Intervention Administration (Weeks 24 - 36) 46
7.5.2. Contacts with the Research Team (Weeks 28, 32, and 36) 46
7.5.3. Planning for End-of-Trial (Weeks 32 - 36) 46
7.5.4. Follow-Up PROs (Week 36) 46
7.5.5. End of Phase 2 / End of Study (Week 36) 47
7.5.6. Buprenorphine Qualitative Interviews (After Study Participation has been Completed) 47
8. Discontinuation of Study Interventions and Study Participation 48
8.1. Discontinuation of Study Interventions 48
8.2. Participant Withdrawal / Discontinuation from the Study 48
9. Adverse Event Ascertainment and Reporting 49
9.1. Definitions 49
9.1.1. Adverse Event 49
9.1.2. Serious Adverse Event 49
9.1.3. Unanticipated Problem Involving Risk to Participants or Others 49
9.1.4. Pre-Existing Condition 50

9.1.5. Adverse Event Reporting Period 50
9.2. Adverse Events of Interest 50
9.3. Anticipated SAEs 51
9.4. Reporting of AEs of Interest, SAEs, and Unanticipated Problems 51
9.4.1. Reporting AEs of Interest 51
9.4.2. Reporting SAEs 52
9.4.3. Reporting Unanticipated Problems 52
9.5. Reporting to the IRB 52
9.5.1. Reporting Process 53
9.5.2. Other Reportable Events 53
9.5.3. SDRC Notifications to Participating Investigators 54
10. Statistical Considerations 55
10.1. Endpoints, Objectives, and Trial Questions 55
10.2. Statistical Considerations for the Phase 1 Randomization 57
10.2.1. Sample Size and Power Analysis for the Primary Objective 57
10.2.2. Statistical Analyses 58
10.2.3. Planned Interim Efficacy and Futility Analyses for the Phase 1 Randomization 61
10.3. Statistical Considerations for the Phase 2 Intervention 63
10.3.1. Acceptability 64
10.3.2. Tolerability 64
10.3.3. Efficacy 65
10.4. Safety Analyses 66
10.5. Tabulation of Individual Participant Data 66
10.6. Populations for Analyses 66
11. Regulatory, Ethical, and Oversight Considerations 68
11.1. Informed Consent Process and Participant Compensation 68
11.2. Study Discontinuation and Closure 68
11.3. Confidentiality and Privacy 69
11.3.1. Certificate of Confidentiality 69
11.4. Future Use of Data 69
11.5. Safety Oversight 70
11.5.1. Clinical Center Medical Monitoring 70
11.5.2. Internal Safety Committee 70
11.5.3. Independent Data and Safety Monitoring Board (DSMB) 70
11.6. Quality Assurance and Quality Control 71
11.7. Data Handling and Record Keeping 72
11.7.1. Study Records Retention 72
11.8. Protocol Deviations 72
11.9. Publications and Data Sharing 74
11.10. Conflict of Interest Policy 74
12. Protocol Amendment History 75
13. References 80
14. Appendix 85
14.1. Schedule of Procedures 86
14.2. PRO Questionnaire Schedule and Activities During Research Team Contacts 87
14.3. Data Safety and Monitoring Board (DSMB) Charter 89

Table 2.1. Systemic Pharmacologic Options for Pain in Hemodialysis: Benefits and Llimitations[20, 21] 13
Table 3.1. Trial Objectives and Outcomes..19
Table 4.1. Secondary Outcomes: Pain and Opioid Use 21
Table 4.2. Composite Outcome of Pain Interference and Opioid Use: Success (+) or No-Success (–) 21
Table 4.3. Secondary Outcomes Reflecting Conditions or Symptoms Associated with Pain and/or Opioid Use .22
Table 6.1. PCST Telehealth Sessions 29
Table 6.2. Buprenorphine Dosing...37
Table 9.1. Reporting to the Single IRB of Record 53
Table 10.1. Scientific Questions that can be Addressed with the Trial Design 56
Table 10.2. Minimum Detectable Differences between the PCST and Usual Care Groups in the Change from Baseline to Week 12 in the BPI Interference Score 57
Table 10.3. Stopping Boundaries of Efficacy and Futility for a Power of 90% and at a Two-Sided Type 1 Error of 5% for an Interim Analysis with One Look at 50% Information Time..62
Table 10.4. Stopping Boundaries of Efficacy and Futility for a Power of 90% and at a Two-Sided Type 1 Error of 5% for an Interim Analysis with Two Looks at 50% and 75% Information Time 63
Table 10.5. Minimum Detectable Differences between the PCST and Usual Care Groups in the Change from Baseline to Week 12 in the BPI Interference..63
Table 10.6. 95% Confidence Intervals for % of Participants Willing to Switch from a Full Agonist Opioid to Buprenorphine...64
Table 10.7. 95% Confidence Intervals for % of Participants who Continue Buprenorphine Until the End of the Study (Week 36) 65
Table 10.8. 95% Confidence Intervals for the Change in BPI Interference Score between Weeks 24 and 36 66
Figure 1.1. Trial Design 11

PRINCIPAL INVESTIGATOR SIGNATURE

STUDY SPONSOR:	National Institute of Diabetes and Digestive and Kidney Diseases
STUDY TITLE:	HOPE Consortium Trial to Reduce Pain and Opioid Use in Hemodialysis
STUDY ID	843471
PROTOCOL VERSION	V1.7; July 7, 2023

I have read the referenced protocol. I agree to conduct the study in accordance to this protocol, in compliance with the Declaration of Helsinki, Good Clinical Practices (GCP), and all applicable regulatory requirements and guidelines.

Principal Investigator Name	______________________	Signature	______________________
Affiliation:	______________________	Date	______________________

Abbreviations

ACP	American College of Physicians
AE	adverse event
BPI	Brief Pain Interference Scale
BUP	Buprenorphine
CATI	computer-assisted telephone interviewing
CDC	The Centers for Disease Control and Prevention
COPES	Cooperative Pain Education and Self-Management
CSQ	Coping Strategies Questionnaire
CRF	case report form
DSMB	Data and Safety Monitoring Board
ESRD	end-stage renal disease
GAD-7	Generalized Anxiety Disorder – 7
GCP	Good Clinical Practices
HIPAA	Health Insurance Portability and Accounting Act
HOPE	Hemodialysis Pain Reduction Effort
IRB	Institutional Review Board
ICC	intra-cluster correlation coefficient
IVR	interactive voice response
MME	morphine milligram equivalents
MOP	Manual of Procedures
MSPSS	Multidimensional Scale of Perceived Social Support
NIDDK	National Institute of Diabetes and Digestive and Kidney Diseases
NIH	National Institutes of Health
NSAIDS	non-steroidal anti-inflammatory drugs
OHRP	Office of Human Research Protections
OUD	opioid use disorder
PA	physician assistant
PCS	Pain Catastrophizing Scale
PCST	pain coping skills training
PGIC	Patient Global Impression of Change
PHI	protected health information
PHQ-9	Patient Health Questionnaire – 9
PRO	patient reported outcome
PROMIS	Patient-Reported Outcomes Measurement Information System
QOL	Quality of Life
MQOL	McGill Quality of Life
SAE	serious adverse event
SDRC	Scientific and Data Research Center
SIS	Single-item scale
SSRIs	selective serotonin reuptake inhibitors
SNRIs	serotonin-nor-epinephrine reuptake inhibitors
SUD	substance use disorder
TAPS	Tobacco, Alcohol, Prescription medication, and other Substance use Tool
TLFB	Timeline Followback
UPenn	University of Pennsylvania
U.S.	United States

1. STUDY SUMMARY

1.1. Synopsis

**Title**	HOPE Consortium Trial to Reduce Pain and Opioid Use in Hemodialysis
**Short Title**	HOPE Trial
**Study Description**	HOPE is a randomized clinical trial that will evaluate approaches to reducing pain and opioid use among patients with chronic pain who are receiving maintenance hemodialysis for end-stage renal disease. The hypotheses are: 1) pain coping skills training is effective at reducing pain and opioid use, and 2) buprenorphine is acceptable and tolerable as an approach for managing opioid physical dependence.
**Objectives**	Primary Objective<br>• To evaluate the effectiveness of pain coping skills training compared with usual care for reducing pain interference (primary outcome), and for improving other pain outcomes, opioid use, and associated conditions (all secondary outcomes) among patients with end-stage renal disease receiving treatment with maintenance hemodialysis<br>Secondary Objective<br>• To explore acceptability, tolerability, and efficacy of buprenorphine in a subgroup of trial participants prescribed moderate to high-dose long-term opioid therapy for chronic pain
**Primary Outcome**	Pain interference as measured by the Brief Pain Inventory Interference Scale
**Secondary Outcomes**	• Pain intensity<br>• Pain catastrophizing<br>• Opioid use<br>• Composite of pain interference and opioid use<br>• Quality of life<br>• Physical functioning<br>• Depression<br>• Anxiety<br>• Coping<br>• Self-efficacy<br>• Sleep quality<br>• Fatigue<br>• Other symptoms<br>• Satisfaction with treatment<br>• Social support<br>• Family intrusion

	• Discrimination<br>• Falls<br>• Hospitalizations<br>• Death<br>• Buprenorphine acceptability<br>• Buprenorphine tolerability
**Study Population**	640 patients being treated with maintenance hemodialysis for end-stage renal disease who have chronic pain, at least 300 of whom have prescription opioid use.
**Phase or Trial Type**	Effectiveness (PCST intervention); Exploratory (buprenorphine intervention)
**Description of Sites/Facilities Enrolling Participants**	Participants are enrolled from multiple dialysis units affiliated with the following 8 Clinical Centers, some of which involve collaborations across multiple medical centers:<br>• Hennepin Health Care<br>• Massachusetts General Hospital<br>• New York University<br>• University of Illinois-Chicago<br>• University of Pittsburgh; University of Pennsylvania<br>• University of Washington; University of New Mexico; Rogosin Institute<br>• Vanderbilt University Medical Center; West Virginia University<br>• Yale University; Multiple Veterans Affairs Healthcare Systems<br><br>The Scientific and Data Research Center is based at the University of Pennsylvania.
**Description of Study Intervention**	1. Pain Coping Skills Training (PCST): Participants will complete a structured, standardized, interactive program designed to reduce pain interference, opioid use, and comorbid symptoms of depression, anxiety, and sleep disturbance. The program is adapted for the hemodialysis patient population, and is delivered using live coaches via video telehealth and with interactive voice response via telephone.<br>2. Usual Care: Participants will be given educational materials and resources about alternatives to opioid pain medications. These materials will also be provided to participants in the PCST group.<br>3. Buprenorphine: In a second phase of the trial, participants in both the PCST and Usual Care groups who meet eligibility criteria, including use of moderate to high-dose prescribed opioid therapy, will be offered buprenorphine as an alternative to a full-agonist opioid pain medication.
**Study Duration**	44 months
**Participant Duration**	36 weeks

1.2. Key Roles and Study Governance

Sponsor	Scientific and Data Research Center
National Institute of Diabetes and Digestive and Kidney Diseases / National Institutes of Health	Perelman School of Medicine;<br>University of Pennsylvania
Project Scientist: Paul L. Kimmel, MD	Principal Investigator: Laura M. Dember, MD
Address:<br>2 Democracy Plaza<br>6707 Democracy Boulevard<br>Bethesda, MD 20892-5458	Address:<br>920 Blockley Hall<br>423 Guardian Drive<br>Philadelphia, PA 19104
Phone Number: 301-594-7717	Phone Number 215-573-5264
Email: kimmelp@extra.niddk.nih.gov	Email: ldember@pennmedicine.upenn.edu

The HOPE Trial Steering Committee includes the Contact Principal Investigator of each of the Clinical Centers and the Scientific and Data Coordinating Center, the NIDDK Project Scientist, the Steering Committee Chair (TBD), and patients. Additional HOPE Consortium committees include: 1) Protocol Committee and Working Groups, 2) Safety Committee, 3) Quality Control Committee, 4) Recruitment and Retention Committee, 5) Publications Committee, and 6) Stakeholder Advisory Committee composed of patients and representatives from dialysis provider organizations.

The external Data and Safety Monitoring Board (DSMB), serves as a protocol review committee and a trial monitoring committee. The members and chair of the DSMB are appointed by the NIDDK.

1.3. Study Schema

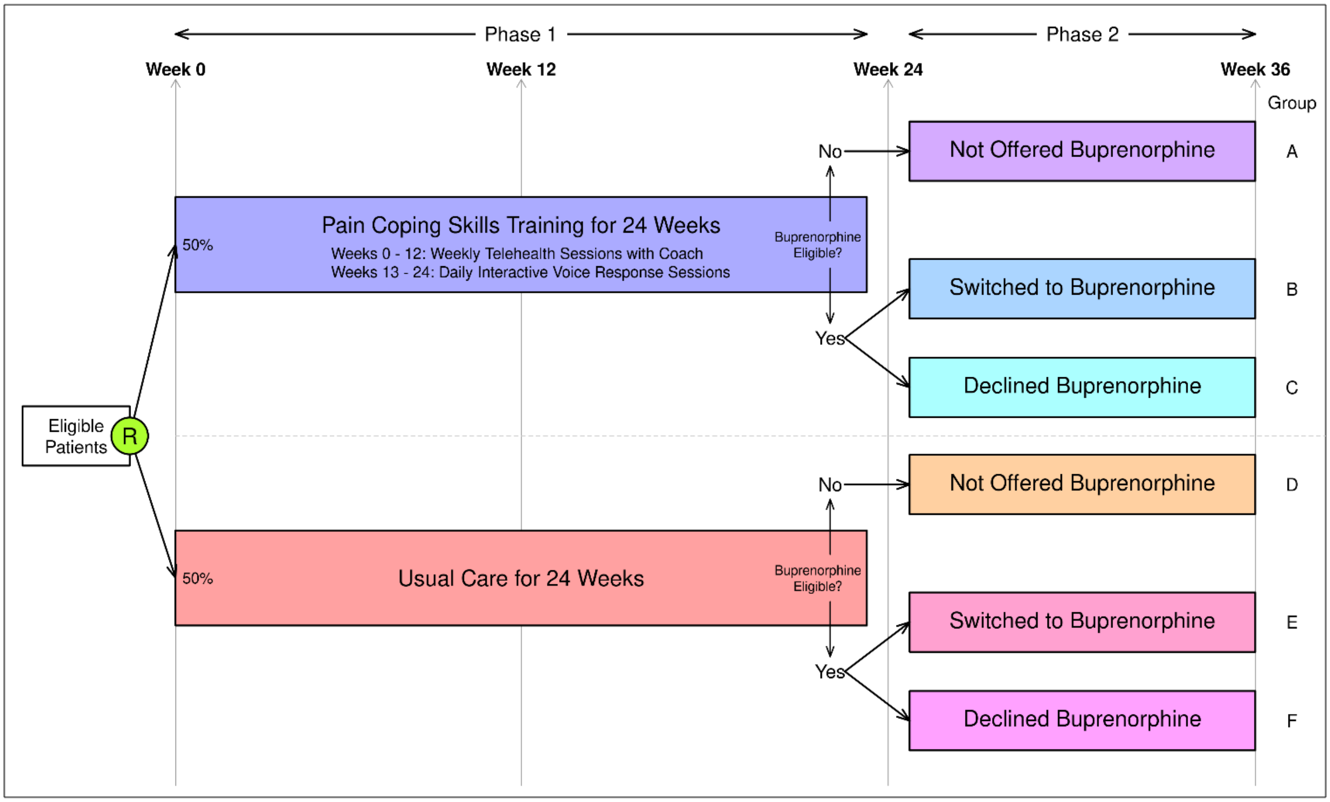

Figure 1.1. Trial Design

The trial uses a sequential multiple assignment design with a randomized component (Phase 1) and a non-randomized component (Phase 2). Patients undergoing treatment with maintenance hemodialysis who have chronic pain, a subset of whom are using prescribed opioid medications, are randomized in equal proportions to the Phase 1 intervention: Pain Coping Skills Training (PCST) or Usual Care for 24 weeks. The PCST intervention has two components each taking place over 12 weeks: 1) weekly telehealth sessions with a PCST coach, and 2) daily interactive voice response (IVR) sessions via telephone. The primary outcome of pain interference will be ascertained at Week 12 coinciding with the end of the PCST weekly coaching sessions. At Week 24, participants in either of the randomized groups who are taking opioids at a dose of ≥ 20 morphine milligram equivalents (MME)/day will be assessed for eligibility for the Phase 2 intervention. Participants who meet eligibility criteria for the Phase 2 intervention will be encouraged to switch from their current full agonist opioid medication to the partial opioid agonist, buprenorphine, as an approach to addressing physical dependence on prescribed opioids. Participants who do not meet the Phase 2 eligibility criteria will not be offered buprenorphine. All participants will continue to be followed from Week 24 until Week 36 for ascertainment of pain, opioid use, and other outcomes to address durability of the effects of PCST, and, for those who switch to buprenorphine, to assess buprenorphine acceptability, tolerability, and efficacy as exploratory outcomes.

2. INTRODUCTION AND RATIONALE

2.1. Study Rationale

With the recognition that many patients with end-stage renal disease (ESRD) prioritize how they feel and function over how long they live, there has been increased effort to address the tremendous symptom burden that accompanies ESRD.[1] Pain is among the most common of these symptoms with approximately 60% of dialysis patients reporting pain that is usually described as moderate or severe.[2] The causes and types of pain in this patient population are numerous and medical management is complicated by altered pharmacokinetic and pharmacodynamic properties of analgesic agents in the setting of kidney failure. Due to both the high prevalence of pain and the limited options for its management, use of opioid medications is common in this population. In 2010, as many as 20% of hemodialysis patients in the United States (U.S.) received prescribed opioids for 90 days or longer.[3] Long term opioid therapy is of questionable benefit for chronic pain,[4, 5] and, among the general population, is associated, in a dose-dependent manner, with an increased likelihood of serious harms including declining functional status, infection, hypogonadism, overdose, and opioid use disorder (OUD).[6, 7] Among hemodialysis patients, long term opioid use is associated with increased rates of falls, hip fractures, hospitalizations, dialysis withdrawal, and death.[3, 8] Patients treated with hemodialysis generally have low levels of physical activity and high rates of insomnia and depression,[9] all of which can exacerbate chronic pain, complicate its management, and be potentiated by opioid use.[10] Non-pharmacologic approaches such as cognitive behavioral therapy, mindfulness, and acceptance and commitment therapy, have demonstrated efficacy for chronic pain in the general population, but have not been studied in ESRD. Buprenorphine, a partial opioid agonist with an improved safety profile compared to other opioids, may be an effective option for reducing opioid use among patients treated with hemodialysis, but there is limited experience with this drug in the ESRD population. Thus, the primary objective of this 36-week trial is to evaluate the effectiveness of a cognitive behavioral therapy-based intervention called pain coping skills training (PCST) for improving pain and reducing opioid use among patients on hemodialysis with chronic pain. A secondary objective is to explore acceptability, tolerability, and efficacy of buprenorphine among patients who are using prescribed moderate to high-dose opioid therapy.

2.2. Background

2.2.1. Prevalence of Pain among Patients Treated with Hemodialysis

In the U.S. chronic pain has a prevalence of 11% among adults and was estimated in 2016 to have an annual cost of $635 billion.[11] Due to a high comorbidity burden, chronic pain is even more common among patients undergoing treatment with maintenance hemodialysis.[12] [13] More than half of hemodialysis patients have diabetes mellitus,[14] often with accompanying painful neuropathy, and most patients have ESRD-associated mineral metabolism disorders that predispose to chronic musculoskeletal pain,[15] and to a fracture risk that is 2- to 4-fold higher than that for the general population.[16] Falls are also common among hemodialysis patients; recent reports indicate that 25-30% of hemodialysis patients experience at least one fall per year and 57% of these individuals reported multiple falls.[17]

Pain in the setting of ESRD and hemodialysis has some key differences from that experienced by the general population. In addition to chronic pain, patients often experience acute pain associated with recurring hemodialysis sessions (typically 3 per week). Sources of hemodialysis-associated pain include severe muscle cramping resulting from fluid removal, needle infiltrations, vascular steal syndrome and other complications of hemodialysis vascular access, and a "washed out" feeling with substantial fatigue and headaches that may persist for up to 12 hours after dialysis sessions. One recent systematic review estimated that 33-82% and 21-92% of dialysis patients have acute and chronic pain, respectively.[18] An earlier systematic review quantified this as a mean prevalence of pain of 47% across 60 studies, with pain during the hemodialysis treatments, including vascular access pain, headache, neuropathic pain, and musculoskeletal pain, occurring most frequently.[12]

2.2.2. Complexity of Pain Management among Patients Treated with Hemodialysis

Pharmacologic options to treat pain are limited for patients receiving maintenance dialysis because of a difficult balance between efficacy and safety for many types of medications (**see Table 2.1**). The high risk-to-benefit ratio results from reduced clearance of agents that are metabolized by the kidneys and from side effects that are of particular significance in the presence of kidney failure. Despite limited evidence for efficacy,[19] long-term opioid therapy, defined as ≥ 90 days of opioid analgesics, has emerged as a dominant treatment paradigm for chronic pain, particularly among maintenance hemodialysis patients.[3] The challenges associated with long-term use of opioid medications, discussed in **Section 2.2.3**, have resulted in a critical need in the dialysis population for better pharmacologic strategies as well as approaches that combine pharmacologic and non-pharmacologic interventions to reduce pain and its deleterious effects.

Table 2.1. Systemic Pharmacologic Options for Pain in Hemodialysis: Benefits and Llimitations[20, 21]

Class	Benefits	Limitations
Non-steroidal anti-inflammatory drugs (NSAIDS)	Effective pain control; inexpensive; no central nervous system effects	May accelerate loss of residual kidney function; gastrointestinal bleeding
COX-2 Inhibitors	Effective pain control; no CNS effects	May accelerate loss of residual kidney function; pro-thrombotic
Acetaminophen	Safe; inexpensive	Often insufficient analgesia; liver toxicity
Tricyclic antidepressants	Likely effective for treating neuropathic pain	Anticholinergic adverse effects, particularly in the elderly
Selective serotonin reuptake inhibitors / serotonin-nor-epinephrine reuptake inhibitors	May be effective for treating neuropathic and headache pain	Dosing considerations for some agents; may worsen restless leg syndrome; QT prolongation with some agents
Gabapentin / Pregabalin	Likely effective for neuropathic pain	High toxicity risk including mental status changes, somnolence, and hypotension
Opioids	May be effective for treating pain in some patients (mixed evidence);	Addiction potential; constipation, mental status changes, somnolence, respiratory depression; mixed evidence for efficacy for chronic pain

2.2.3. Potential Harms from Long Term Opioid Therapy

In the non-dialysis population, observational data and limited clinical trial data suggest that long-term opioid therapy is associated with potential harms without significant improvements in chronic pain. In addition to a higher risk of overdose death among long-term opioid recipients,[22] accumulating data show dose-dependent associations with hypogonadism, infection,[6] osteoporosis, falls, fractures, motor vehicle accidents, opioid use disorder (OUD), and intermittent withdrawal symptoms,[7] and, additionally, among hemodialysis patients, with increased rates of hospitalizations, dialysis withdrawal, and death.[3] For the general population, the Centers for Disease Control and Prevention (CDC) recommends using non-opioid medications instead of opioids for chronic pain. For patients already taking opioids, the CDC recommends that providers use caution if increasing opioid doses above 50 MME/day and avoid dose escalations to beyond 90 MME/day.[23] For the dialysis patient population, data to guide opioid dosing and alternative pain management strategies are lacking.[21] For example, because of its hepatic metabolism and elimination, methadone is a favored opioid in the setting of kidney failure;[24] however, methadone has multiple drug interactions and prolongs the QT interval which may have important implications given the high rates of sudden cardiac death among hemodialysis patients.

2.2.4. The Need for Non-Pharmacologic Approaches to Pain Management and Opioid Reduction in the Setting of Maintenance Hemodialysis

Individuals receiving maintenance hemodialysis are an exceedingly vulnerable population. Mortality rates are similar to those for metastatic cancer,[25] the burden of pain, prevalence of frailty, and rates of physical and cognitive impairment are high, and there are few well-studied strategies for managing symptoms. As is evident in **Table 2.1**, pain management is challenging in the setting of kidney failure. Non-steroidal anti-inflammatory drugs are often contraindicated because of the need to maintain residual kidney function and the risk of gastrointestinal bleeding, and the use of other agents, such as gabapentin and pregabalin,[26] is limited by toxicities including orthostasis, encephalopathy, somnolence, and falls. Many opioids are metabolized and cleared differently when kidney function is reduced, increasing the risk of adverse effects of some agents. Patients with dialysis-dependent ESRD may be more vulnerable to the neurocognitive, endocrine and infectious complications of opioids, but they may also be more vulnerable to the complications associated with non-opioid based pain regimens and, in the absence of pain control, may become increasingly debilitated. Accordingly, safely minimizing the toxicities of pain management strategies while maximizing overall levels of functioning is a critical goal for this patient population. These complexities underscore the need for targeted strategies to incorporate non-pharmacological approaches to pain management.

2.2.5. Rationale for a Pain Coping Skills Training Intervention to Address Pain and Opioid Use

Cognitive behavioral therapy directed at pain coping skills has a strong evidence base and is among the most widely studied non-pharmacological treatments for chronic pain.[27] Providing cognitive behavioral coping skills training is an attractive adjunct or alternative to pharmacological treatments because its effects can persist after treatment is discontinued without the negative side effects and potential harms of pharmacological treatments such as opioids. The goal of a cognitive behavioral pain coping skills intervention is to help patients develop skills to manage pain, and its associated disability and emotional distress, as a means to improve functioning and quality of life.[28] Skills include reframing maladaptive cognitive processes (e.g., catastrophizing)

and promoting adaptive behaviors (e.g., relaxation). Cognitive behavioral interventions have been recommended by the CDC[23] and American College of Physicians (ACP)[29] as a first-line treatment to reduce pain and improve function.

Newer treatments such as mindfulness and acceptance and commitment therapy share commonalities in their underlying rationale and treatment strategies with standard cognitive behavioral therapy. Because of these commonalities and lack of single universally accepted protocol for these treatments for pain, many interventions for chronic pain combine techniques from all of these treatments.

2.2.5.1. Efficacy of Interactive Voice Response for Chronic Pain Cognitive Behavioral Interventions

In addition to providing pain coping skills training through telehealth-delivered real-time sessions between a coach and a patient, the cognitive behavioral intervention for this trial includes an interactive voice response (IVR) component that is based on the recently developed and tested COPES (Cooperative Pain Education and Self-Management) program.[30] In a non-inferiority trial that compared an in-person cognitive behavioral intervention to COPES in a non-ESRD patient population, post-treatment statistically significant improvements in physical functioning, sleep quality, and physical quality of life relative to baseline occurred with both treatments, with no significant advantage for either treatment.[30] Additionally, patients were equally satisfied with COPES and the in-person intervention. Importantly, work by Naylor and colleagues demonstrated that IVR-based booster sessions, similar to those that will be used in this trial, following a traditional cognitive behavioral intervention produced maintenance of treatment gains and continued reduction in opioid medication use.[31]

The IVR treatment infrastructure that has been developed and tested in prior trials will provide foundational resources for this trial. In prior studies, participants completed 85-90% of scheduled daily calls. The COPES manual includes an extensive library of feedback scripts that have been refined based on post-treatment participant interviews and feedback with participants from the original COPES trial. These feedback scripts will serve as the basis for the personalized feedback in the planned IVR booster intervention.

2.2.5.2. Adaptation of Pain Coping Skills Training Interventions to the Hemodialysis Population

Patients undergoing hemodialysis have high rates of depression, and the presence of depressive symptoms is known to be associated with dialysis withdrawal, hospitalization, mortality, and non-adherence with dialysis treatments.[32-34] Additionally, low rates of physical activity, poor sleep, and quality of life are well-documented in this population.[35-37] Cognitive behavioral skills may be effective for improving depression, quality of life, and sleep, and increasing physical activity and adherence behaviors among patients treated with hemodialysis.[38] For the HOPE Trial, the Pain Coping Skills Training (PCST) intervention will retain the critical components of traditional behavioral pain management skills but will be expanded to include skills for mood regulation, sleep improvement, and anxiety management, all core issues for this patient population. A focus will be on highlighting how these conditions and their treatment overlap and interact, e.g., walking improves pain and physical function, or cognitive restructuring can address pain catastrophizing as well as beliefs that impede hemodialysis adherence or maintain depressive symptoms. Additionally, the intervention will promote use of

skills such as guided mindfulness and progressive muscle relaxation during hemodialysis sessions, a setting well-suited for these types of interventions.

2.2.6. Rationale for a Buprenorphine Intervention to Address Opioid Physical Dependence in Hemodialysis Patients

Buprenorphine is a partial opioid agonist with analgesic properties similar to those of full-agonist opioids. Various formulations of buprenorphine are available for the treatment of opioid use disorder and for pain. Because buprenorphine causes less respiratory depression than full agonist opioids, it is considered to be safer and is designated as a schedule III controlled substance, in contrast to schedule II full agonists such as oxycodone, hydrocodone and others.[39, 40] Among patients on long-term opioid therapy for chronic pain, higher prescribed opioid daily doses (compared with lower daily doses) are associated with worse pain-related function and more frequent adverse outcomes. Limited evidence suggests that opioid dose reduction can improve pain, function, and quality of life; however, physical dependence makes it difficult for many patients prescribed long-term opioids to reduce their opioid use. Buprenorphine addresses physical dependence by preventing withdrawal symptoms and reducing cravings. Patients who have transitioned from high-dose opioids to buprenorphine often have improved pain/function and decreased opioid-related adverse effects.[41, 42] In a large controlled case series, buprenorphine was effective for successful discontinuation of full-agonist opioids by patients on long-term opioid therapy for pain; due to marked physical opioid dependence, patients in this study used higher dose buprenorphine formulations approved for treating opioid use disorder,[43] as opposed to the lower dose formulations approved for pain. Benefits of switching from full-agonist opioids to buprenorphine are hypothesized to be due to effective management of physical dependence, which allows patients to more easily reduce their overall opioid use. Buprenorphine may also have fewer adverse cognitive and emotional effects than other opioids. Patients receiving maintenance hemodialysis for kidney failure may be especially vulnerable to adverse effects associated with use of long-term opioids and may experience these adverse effects at lower daily dosages than other patients. Thus, strategies are needed to help this patient population reduce opioid use and, ultimately, reduce pain interference and opioid adverse effects. The HOPE Consortium trial will evaluate, in an exploratory manner, the acceptability, tolerability, and potential efficacy of buprenorphine for physical dependence on opioids in hemodialysis patients on moderate to high-dose long-term opioid therapy.

2.2.6.1. Buprenorphine Pharmacokinetics, Pharmacodynamics, and Toxicology

Buprenorphine taken orally undergoes extensive first-pass metabolism but its bioavailability with sublingual administration makes this a feasible route of administration. The mean time to maximum plasma concentration of buprenorphine following sublingual administration is variable, ranging from 40 minutes to 3.5 hours. Buprenorphine has a large volume of distribution and is highly protein bound (96%). The drug is extensively metabolized by N-dealkylation to norbuprenorphine, primarily through cytochrome P450 (CYP) 3A4 (i.e., liver, not renal, metabolism). The terminal elimination half-life of sublingually administered buprenorphine is 22 to 44 hours. Approximately 10-30% of buprenorphine is excreted in the urine. Some formulations of buprenorphine include naloxone as a deterrent to misuse via injection, but naloxone is inert when buprenorphine/naloxone is used as directed. Naloxone does not appear to influence the

pharmacokinetics of buprenorphine when the combination buprenorphine/naloxone product is taken as prescribed (sublingually). Buprenorphine crosses the placenta during pregnancy and also crosses into breast milk. It is not necessary to modify dosing of buprenorphine in kidney failure, and the safety of buprenorphine in ESRD and compatibility with hemodialysis are established.[44,45] The half-life of buprenorphine may be prolonged in individuals with severe chronic liver disease because of reduced CYP3A4 activity. Buprenorphine is not removed by dialysis.[45]

2.3. Risk / Benefit Assessment for the HOPE Trial Interventions

2.3.1. Known Potential Risks of Trial Interventions

The PCST intervention may increase distress for participants if sensitive topics are raised during the telehealth or IVR sessions. All prescribing decisions for participants will be made by the treating physicians rather than by the research team. Participants in both the PCST and Usual Care groups may have adverse effects from the non-opioid pain medications that are prescribed by treating (non-study) clinicians during the course of the study. Patients may experience withdrawal symptoms and other temporary discomfort due to reducing opioid doses, switching from one opioid medication to another, or transitioning to non-opioid medications. Because opioids may reduce the symptoms of restless leg syndrome in hemodialysis patients, reducing or discontinuing opioids may worsen this condition. Intervention clinicians will educate participants about withdrawal symptoms, take precautions to prevent withdrawal, and manage symptoms if they occur.

Common adverse effects of buprenorphine, the Phase 2 intervention, are similar to those of other opioids and include dizziness, headache, sedation, dry mouth, mouth numbness, constipation, insomnia, abdominal pain, drowsiness, fatigue, and numbness or tingling. An FDA report issued on January 12, 2022 identified dental issues such as tooth decay, cavities, oral infections, and loss of teeth, as newly recognized possible adverse effects of buprenorphine delivered via buccal or sublingual approaches. The FDA advises that these dental problems have been reported even in patients with no history of dental issues. The risk of these dental problems can be reduced by rinsing the mouth with water after using the drug, waiting at least an hour before teeth brushing, visiting a dentist shortly after starting buprenorphine, and having regular dental checkups. The FDA maintains that despite these risks, the benefits of buprenorphine medicines clearly outweigh the risks. As for all opioids, additive or synergistic interactions can occur between buprenorphine and central nervous system depressants including benzodiazepines and alcohol. Active, untreated substance use disorder is an exclusion criterion for the trial. Study investigators will educate participants eligible for the buprenorphine intervention about withdrawal symptoms, take precautions to prevent withdrawal, and manage symptoms if they occur.

The trial will not exclude people who are pregnant. Pregnancy is rare in patients undergoing maintenance hemodialysis. The PCST intervention does not require any specific medications or activities with pregnancy-associated risks, and for participants who are pregnant, lactating, or could possibly be pregnant, contraindicated medications will not be recommended by the research teams. People who are pregnant or people with child-bearing potential who are not willing to use acceptable forms of contraception will not be eligible for the buprenorphine intervention (**see Study Schema in Section 1.2**) since opioid withdrawal during pregnancy may increase the risk of miscarriage and preterm labor.

2.3.2. Known Potential Benefits of Trial Interventions

As described above, demonstrated benefits of CBT-based interventions in the non-ESRD setting include reduced pain interference and pain intensity, reduced opioid use, and improved quality of life. In the ESRD setting, CBT has been shown to be an effective treatment for depression.[46] Buprenorphine improves function and quality of life in individuals with opioid dependence and has demonstrated analgesic efficacy. Buprenorphine has a markedly lower risk of overdose compared to full agonist opioids so its use could decrease long-term overdose risk.

2.3.3. Assessment of the Balance between Risks and Benefits

Risks of the trial interventions, described above, are similar to those in routine clinical care. Benefits and risks of recommended therapies will be discussed with patients as in clinical practice. To address the potential for increased distress from discussing sensitive topics in the PCST arm, the PCST coaches will be trained to manage such distress in accordance with accepted standard practices, and there will be a protocol in place for protective transfer should greater intensity of services be indicated. Buprenorphine is an FDA-approved medication for which there is more than 15 years of post-marketing data, and a risk profile that is similar among patients with and without impaired kidney function. Buprenorphine is a schedule III controlled substance, meaning its risk for abuse is considered by the DEA to be lower than the risk with the opioid medications likely to be used by patients enrolled in the trial (mostly schedule II opioids). Eligibility for the buprenorphine component of the trial intervention will be based on specific criteria assessed at Week 24 and use of buprenorphine will require additional informed consent.

Overall, the benefits of trial participation outweigh the risks based on the following considerations: 1) there are substantial potential benefits including improved pain management, decreased opioid-related adverse effects, and improved safety, and 2) the known risks will be minimized through individualized care decisions consistent with standards of care.

3. STUDY OBJECTIVES

The overall objective of the trial is to evaluate the effectiveness of approaches to reducing pain and opioid use among individuals receiving treatment with maintenance hemodialysis for ESRD. The specific objectives and outcomes are summarized in **Table 3.1**. Additional information about the outcomes and methods for their ascertainment is provided in **Section 4.3**.

Table 3.1. Trial Objectives and Outcomes

Objectives	Outcomes	Justification for Outcomes
**Primary**		
To evaluate the effectiveness of pain coping skills training compared with usual care for reducing pain interference, opioid use, and related outcomes among patients with end-stage renal disease receiving treatment with maintenance hemodialysis	The primary outcome is reduction in pain interference, as measured by the Brief Pain Inventory Interference scale.<br>Secondary outcomes include:<br>• Pain intensity<br>• Composite of pain interference and opioid use<br>• Opioid use<br>• Pain catastrophizing<br>• Quality of life<br>• Depression<br>• Anxiety<br>• Coping<br>• Self-efficacy<br>• Sleep quality<br>• Fatigue<br>• Other symptoms<br>• Satisfaction with treatment<br>• Social support<br>• Family intrusion<br>• Discrimination<br>• Falls<br>• Hospitalization rate<br>• Death	The primary outcome, pain interference, is important both patients and providers, and has been recommended by expert consensus groups as one of the most important measures for chronic pain trials.<br><br>The secondary outcomes include conditions or symptoms often associated with chronic pain and/or opioid use, or clinical events that can result from medications used to manage pain.
**Exploratory**		
To explore acceptability, tolerability, and efficacy of buprenorphine among hemodialysis patients receiving moderate to high-dose long-term opioid therapy for chronic pain	• Acceptability to patients, as measured by the proportion of participants who initiate buprenorphine from among those participants who are offered buprenorphine.<br>• Tolerability, as measured by the proportion of participants who do not discontinue buprenorphine due to adverse effects or intolerance.<br>• Efficacy, as assessed by the Brief Pain Inventory Interference scale	Assessment of acceptability and tolerability of buprenorphine is needed before conducting definitive large scale trials of buprenorphine in the hemodialysis patient population.

4. STUDY DESIGN AND OUTCOMES

4.1. Trial Design and Rationale

The primary trial hypothesis is that pain coping skills training will improve pain and opioid use outcomes more than usual clinical care. The design is a multicenter, sequential, multiple assignment trial with a randomized component (Phase 1) and a non-randomized component (Phase 2). In Phase 1 (Weeks 1 - 24), participants will be randomized into one of two treatment groups: 1. Pain Coping Skills Training (PCST) for 24 weeks, or 2. Usual Care. The PCST intervention has two sequential components each taking place over 12 weeks: 1) weekly telehealth sessions with a PCST coach, and 2) daily interactive voice response (IVR) sessions via telephone. The primary outcome of pain interference will be ascertained at Week 12 which coincides with the end of the PCST weekly coaching sessions. In Phase 2, beginning at Week 24, participants in both randomized groups will be assessed for eligibility for a second intervention, buprenorphine, as an alternative to full agonist opioid pain medication. The main eligibility criterion for the buprenorphine intervention is current use of prescribed opioids at an average dose of ≥ 20 morphine milligram equivalents (MME)/day. Participants who meet the buprenorphine eligibility criteria will be encouraged, but not required, to switch from their current opioid medication to buprenorphine to explore buprenorphine as an approach to addressing physical opioid dependence in patients receiving maintenance hemodialysis. Participants who do not meet the buprenorphine eligibility criteria will not be offered buprenorphine. All participants will continue to be followed from Week 24 until Week 36 (Phase 2) for ascertainment of pain, opioid use, and other outcomes to address durability of the effects of PCST, and, for those who switched to buprenorphine, to assess buprenorphine acceptability, tolerability, and efficacy as exploratory outcomes.

Randomization will be performed at the level of the individual participant using permuted blocks with stratification by enrolling site and by the presence or absence of opioid use using the criteria provided in **Section 5.3** for the Phase 1 randomization. Treatment assignments will not be masked to participants or research team members because of 1) impracticability, and 2) an interest in implementing the interventions as they would be outside of a trial setting. The trial includes a control group that does not receive an active intervention beyond usual clinical care so it is possible to determine whether changes observed in the active treatment groups are the result of the interventions or the result of either increased attention that accompanies trial participation or the natural history of the condition. Bias will be minimized by performing randomization after collection of baseline data, concealing the allocation order, and ascertaining the patient-reported outcomes, including the primary outcome, using standardized, centrally administered questionnaires by research personnel blinded to treatment assignment. Safety and tolerability will be evaluated through ascertainment of specific adverse events of interest and serious adverse events throughout the duration of the trial.

4.2. Outcomes

Citations and references for the instruments or questionnaires used for the outcome ascertainment are provided in **Appendix 14.2** and **Section 13**, respectively.

4.2.1. Primary Outcome: Pain Interference

Pain interference will be measured by the Brief Pain Inventory Interference Scale (BPI Interference)[47] which has a 7-day look-back period. The BPI is a multidimensional measure developed for cancer pain, but subsequently validated in diverse populations, including patients with chronic pain.[47, 48] BPI is composed of the severity subscale (four pain intensity items) and the interference subscale (seven pain interference items). Pain interference has been recommended by expert consensus groups as a key outcome measure in chronic pain trials.[49] Interference, or the degree to which pain interferes with various domains of function, is thought to have greater salience with patients and greater generalizability across populations than pain intensity.[47, 50-53] The BPI Interference scale, specifically, has demonstrated responsiveness to change in numerous clinical trials.[54-56]

4.2.2. Secondary Outcomes

The secondary outcomes will include: 1) patient-reported indicators of pain and opioid use, 2) patient-reported indicators of conditions or symptoms that often accompany pain and/or opioid use, 3) clinical outcomes, and 4) acceptability and tolerability outcomes.

4.2.2.1. Secondary Pain and Opioid Use Outcomes

As secondary outcomes, pain and opioid use will be assessed using the instruments or methods shown in **Table 4.1** and analyzed as continuous measures.

Table 4.1. Secondary Outcomes: Pain and Opioid Use

Domain	Instrument or Data Source	Look-Back Period	Time-Points for Assessment
Pain intensity	Brief Pain Inventory (BPI) Severity	7 days	Weeks 0, 12, 24, 36
Pain catastrophizing	Pain Catastrophizing Scale – SF 6	None	Weeks 0, 12, 24, 36
Opioid use	Timeline Followback	14 days	Weeks 0, 12, 24, 36
Composite of pain and opioid use	BPI Interference / Timeline Followback	7 days/14 days	Weeks 0, 12, 24, 36

4.2.2.1.1. <u>Composite Outcome of Pain and Opioid Use</u>

One of the secondary outcomes is a composite outcome of pain and opioid use as shown in **Table 4.2**. Both a reduction in pain interference with stable or decreased opioid use, and a reduction in opioid use with stable or decreased pain interference will be categorized as success.

Table 4.2. Composite Outcome of Pain Interference and Opioid Use: Success (+) or No-Success (–)

	Pain Less	Pain Stable	Pain More
**Opioid Use Less**	+	+	–
**Opioid Use Stable**	+	–	–
**Opioid Use More**	–	–	–

Criteria for Less, Stable, and More Pain

For the composite outcome of pain and opioid use, the criteria for less, stable, and more pain are based on the change from the baseline BPI Interference score using the following thresholds:[57]

Pain Less: Decrease in BPI Interference score of > 1 point

Pain Stable: Change in BPI Interference by 0 – 1 point in either direction

Pain More: Increase in BPI Interference by > 1 point

Criteria for Less, Stable, and More Opioid Use

For the composite outcome of pain and opioid use, the criteria for less, stable, and more opioid use are based on the difference from baseline in average MME/day using the following thresholds:

Opioid Use Less: ≥ 25% reduction in average MME/day

Opioid Use Stable: < 25% reduction and < 10% increase in average MME/day

Opioid Use More: ≥ 10% increase in average MME/day

The rationale for considering an increase in MME/day of up to < 10% as stable opioid use is to account for sporadic, unpredictable acute pain events or clinically unimportant increases in chronic opioid use.

4.2.2.2. Secondary Outcomes Reflecting Conditions or Symptoms Associated with Pain and/or Opioid Use

The effects of the interventions on several conditions or symptoms often associated with pain and/or opioid use will be evaluated through ascertainment of a set of patient-reported outcomes using validated instruments (see **Table 4.3**).

Table 4.3. Secondary Outcomes Reflecting Conditions or Symptoms Associated with Pain and/or Opioid Use

Domain	Instrument or Data Source	Time-Points for Assessment
Depression	Patient Health Questionnaire (PHQ)-9	Weeks 0, 12, 24, 36
Anxiety	Generalized Anxiety Disorder (GAD)-7	Weeks 0, 12, 24, 36
Sleep quality	PROMIS Sleep Disturbance 6a + Sleep Duration Question	Weeks 0, 12, 24, 36
Fatigue	PROMIS Fatigue SF 6a	Weeks 0, 12, 24, 36
Other symptoms	Dialysis Symptom Index	Weeks 0, 12, 24, 36
Physical functioning	PROMIS Physical Functioning SF 6b	Weeks 0, 12, 24, 36
Quality of life	Single-Item QOL Scale [SIS] from McGill Quality of Life (MQOL)	Weeks 0, 12, 24, 36
Coping	Coping Strategies Questionnaire: 1-item version	Weeks 0, 12, 24, 36
Self-efficacy	PROMIS Self-Efficacy for Managing Chronic Conditions – Managing Symptoms – Short Form 8A + single item targeting self-efficacy for pain	Weeks 0, 12, 24, 36
Discrimination	Everyday Discrimination Scale	Weeks 0, 36
Satisfaction with treatment	Patient Global Impression of Change	Weeks 0, 12, 24, 36
Family intrusion	PROMIS Satisfaction with Social Roles and Activities	Weeks 0, 12, 24, 36
Social support	Multidimensional Scale of Perceived Social Support (MSPSS)	Weeks 0, 12, 24, 36

4.2.2.3. Secondary Outcomes: Clinical Events

The trial interventions are hypothesized to reduce the frequency of several clinical events that will be treated as secondary outcomes.

- Falls
- Hospitalizations
- Deaths

4.2.2.4. Exploratory Outcomes: Acceptability and Tolerability of Buprenorphine

Participants who meet the eligibility criteria for buprenorphine will be encouraged, but not required, to initiate buprenorphine. The buprenorphine acceptability and tolerability outcomes are:

<u>Acceptability</u>

1. The proportion of participants who initiate buprenorphine among those who are offered the buprenorphine intervention.
2. Reasons for not initiating buprenorphine will be ascertained via questionnaire.

<u>Tolerability</u>

1. The proportion of participants who <u>do not</u> discontinue buprenorphine due to adverse effects among those who initiate buprenorphine
2. Medication-related side effects assessed via questionnaire.

4.2.3. Patient-Reported Outcome (PRO) Ascertainment Approach

The primary and secondary patient-reported outcomes for participants at each of the clinical sites will be captured using computer-assisted telephone interviewing (CATI), administered by a centralized team who will be masked to patient treatment assignments.

CATI is a highly reproducible approach for patient-reported outcomes with successful implementation in several multi-center clinical trials in hemodialysis including the Frequent Hemodialysis Network studies and ASCEND,[46] and is currently being used for SLEEP-HD (NCT03534284) and the Hemodialysis Novel Therapies ACTION trial (NCT03141983). The approach allows for participation of patients with a wide range of health literacy and limitations in vision and manual dexterity, and reduces bias in assessing patient-reported outcomes. An interviewer blinded to the treatment assignment will administer the English or Spanish versions of the patient-reported outcome measures made available to the interviewer through the web-based study portal in sequential screens in a fixed sequence starting with the Brief Pain Inventory Interference scale. The study coordinator at each site will schedule the date and time for the participant to receive a phone call for outcome ascertainment. The participant will choose whether to receive the phone call at home on a non-dialysis day (preferred) or at the dialysis unit. Efforts will be made to ensure that all subsequent calls occur at the same site as the baseline assessment. The research team at each site will be equipped with mobile phones that can be made available to participants for the calls, if needed. Each call is expected to last approximately 45 minutes (see **Appendix 14.1** for the instruments included in the full and partial sets of PROs).

The CATI team will be represented on the regular teleconferences of the Steering Committee, Recruitment and Retention Committee, and Quality Control Committee, and will review data capture bi-weekly with the SDRC. A data tracking and scheduling report will be established by the SDRC. The report will prompt the clinical sites to update contact information and vital status prior to data collection calls. To the extent possible, the calls will be performed within a 10-day window around the target date.

4.2.4. Timing of Ascertainment of Primary Outcome

The primary outcome for the trial will be ascertained at Week 12 which coincides with the end of the PCST weekly coaching sessions. The rationale for ascertaining the primary outcome at the end of the weekly coaching sessions, rather than after the IVR component of PCST, is as follows:

1. The initial component of the PCST intervention that is administered by a live coach using a cognitive behaviorally oriented approach is the component of the PCST intervention that has established efficacy in other patient populations.
2. The initial component of the PCST intervention is more readily implemented in clinical practice than is the IVR component due to shorter duration and lack of need for additional technical resources.
3. Assessment of the primary outcome at Week 12 rather than Week 24 protects against loss to follow-up because of kidney transplantation, medical events, or death.

5. STUDY POPULATION

The eligibility criteria for trial participation are provided in **Sections 5.1 and 5.2**. The criteria for the buprenorphine intervention are provided in **Section 6.3.2**.

5.1. Inclusion Criteria

1. Age ≥ 18 years
2. Undergoing in-center maintenance hemodialysis for ≥90 days
3. English- or Spanish-speaking
4. Chronic pain defined as a response of "Most days" or "Every day" to the following question: "In the past 3 months, how often have you had pain?" Answer options: Never, Some days, Most days, Every day
5. Current PEG[58] score ≥ 4
6. Willing to provide informed consent
7. Willing to allow research team to obtain opioid pharmacy refill data
8. Willing to allow research team to contact and work with their opioid prescriber

5.2. Exclusion Criteria

1. Current opioid use disorder
2. Current use of heroin
3. Current non-opioid substance use disorder with the exception of tobacco use disorder
4. Current use of methadone, buprenorphine, or naltrexone for opioid use disorder
5. Current receipt of hospice care
6. Cognitive impairment that, in the judgement of the research team, precludes trial participation
7. Active suicidal intent based on an initial screening with PHQ-9 question #9 followed by further assessment when indicated (see **Section 6.5.4.**)
8. Unstable bipolar disorder, schizophrenia, post-traumatic stress disorder, or other psychotic disorder
9. Life expectancy < 6 months
10. Expected to receive a kidney transplant, transfer to another dialysis facility, or transition to home dialysis within 6 months
11. Current incarceration
12. Any other condition that the investigator considers precludes participation in the clinical trial

5.3. Subgroup with Current or Recent Opioid Use

During eligibility screening all potential participants will have opioid use ascertained using the timeline follow back approach. The trial will enroll at least 300 participants (among the 640 total study participants) with current or recent opioid use defined as patient-reported prescription opioid use during at least 3 of the past 6 months. The number of participants in the opioid use subgroup will be monitored throughout the trial enrollment period. If the rate of enrollment into the opioid use subgroup is lower than targeted, trial enrollment will be restricted to individuals meeting the opioid use criteria as long as this restriction will not compromise the ability of the trial to meet its overall enrollment goal by the target completion date.

5.4. Participant Recruitment and Eligibility Screening

Patients being treated in participating dialysis facilities will be invited to complete a brief pre-screening survey. They will be informed that the purpose of the survey is to identify people who might be appropriate for subsequent screening for trial participation. The pre-screening survey will include one item about chronicity of pain, the PEG 3-item scale and a request for basic contact information (name and phone number). Patients will have the option to opt-out of the pre-screening. The survey also provides an opportunity to indicate a preference to not be contacted about the trial in the future. Completion of the pre-screening survey is not required for study participation. Patients may be approached for consent and subsequent screening activities without having completed the pre-screening survey.

All patients meeting the pain chronicity criterion will be approached for their willingness to undergo screening activities for participation in the clinical trial. Patients who agree to undergo screening will be asked to sign the trial consent form for the 36-week trial (Phases 1 and 2) prior to any further research-related activities. Dialysis unit laboratory studies, medical records at Clinical Centers, and treatment or history records at local dialysis units may be reviewed as part of the screening process. Informational materials may be disseminated at participating dialysis units in order to allow potential participants to learn about the study and to contact investigators if interested. All study material must be approved by local IRBs before dissemination to potential study participants.

5.5. Re-Screening for Eligibility

Individuals who do not meet the eligibility criteria for one or more reasons can be screened again after 30 days. There is no limit to the number of times an individual can be screened for participation in the trial.

6. STUDY INTERVENTIONS

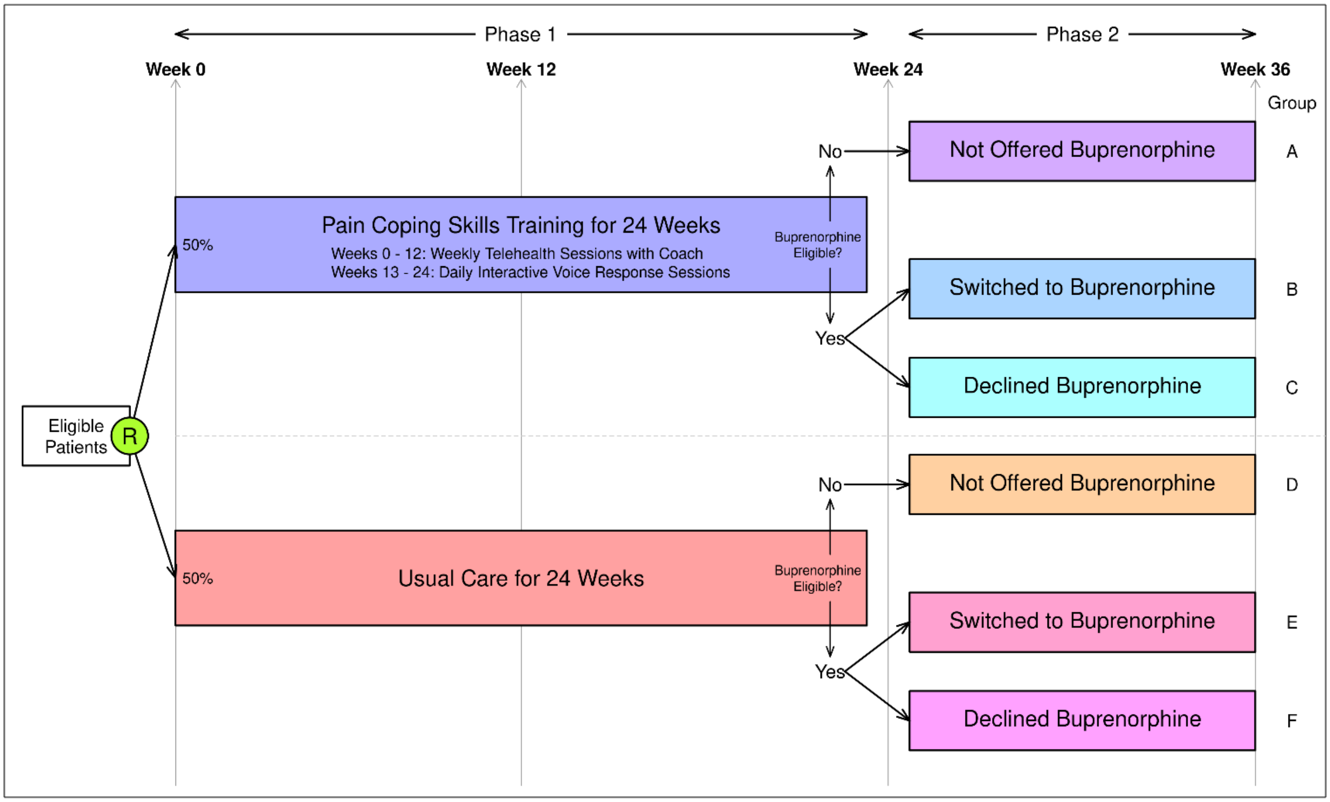

Figure 1.1 and described in **Section 4.1**, participants will be initially randomized to Pain Coping Skills Training for 24 weeks, or Usual Care. At Week 24, all participants will be assessed for eligibility for the buprenorphine intervention. Buprenorphine-eligible participants – determined by current opioid use, will be encouraged, but not required, to switch from their full agonist opioid medication to buprenorphine.

6.1. Pain Coping Skills Training Intervention (PCST)

The PCST intervention will focus primarily on reducing pain interference in daily activities as well as other factors associated with pain, such as negative emotions and sleep disturbance, and will incorporate a strong emphasis on improving pain self-management skills. For participants who have recent or current opioid use, the PCST intervention will include motivational interviewing aimed at reducing opioid use. For participants who do not have recent or current opioid use, motivational interviewing skills will be focused on any identified adherence goals or a value-based skill that the participant is interested in acquiring. Although many components of PCST will be delivered in a structured manner, the approach will allow flexibility to adjust to a participant's personal circumstances.

The PCST training will be delivered by coaches using telehealth during Weeks 1 - 12 and Interactive Voice Response (IVR) during Weeks 13 - 24. The telehealth component will consist of weekly sessions, each lasting 45-50 minutes. The IVR content, intended to serve as booster sessions, will be delivered with daily telephone

interactions, each lasting 5 minutes. Both components of the intervention will be available in English and Spanish.

6.1.1. Rationale for Using Telehealth and IVR as Delivery Modes for PCST

The rationale for using telehealth and IVR rather than in-person visits or a fully computerized approach to deliver the PCST intervention is based on the following considerations:

1. Access to traditional in-office behavioral therapy is challenging for patients treated with hemodialysis because of the substantial time commitment required for dialysis treatments, the high symptom burden associated with ESRD, the high frequency of complications during dialysis treatments, and transportation challenges.
2. Using “chair-side” in-person sessions at the dialysis unit during dialysis treatments is resource intensive, inefficient for the coaches due to time involved in driving to dialysis units, and poses challenges for maintaining privacy.
3. Based on prior experience, it is anticipated that fully computerized cognitive behavioral skills training programs will be challenging for a substantial proportion of trial participants due to limited comfort with technology and/or low vision or manual dexterity.
4. Telehealth using live video interaction via tablets maximizes treatment efficiency and fidelity, while preserving the engagement and alliance-formation that occurs with in-person interactions.
5. IVR provides reinforcement of skills learned during the telehealth sessions with minimal time or technology burden for participants.

6.1.2. Telehealth Implementation

The following tiered approach to telehealth implementation will be used:

1. Skill sessions will be conducted individually through face-to-face interactions with patients via a fully interactive Health Information Portability and Accountability Act (HIPAA) compliant video telehealth platform on the patient’s own device. For a subset of participants, the audio portions of the sessions will be recorded. Participants will receive the intervention at the location of their choice – at a dialysis facility while undergoing hemodialysis, at their home, or at any location of their choice.
2. If the participant does not have a video-enabled device, a tablet computer will be provided by the study team for use in the hemodialysis facility.
3. If the participant cannot or will not engage in the intervention, or a particular session, through a video telehealth platform, the live interaction will be done via the participant’s telephone.
4. If the participant does not have a telephone for use for the study, one will be provided by the study.

Video and audio sessions will be conducted using secure, HIPAA compliant telehealth portals routinely used for clinical care and research. For sessions conducted during hemodialysis treatments, the tablets will be pre-programmed to connect to the secure Wi-Fi, and configured to enable a simple one-touch connection. To ensure privacy, highly sensitive microphones attached to a headphone set are used, so that patients can talk softly and privacy is increased. Sensitive questions will be framed in a way that allows participants to respond using few words. Safety is ensured by reminding patients during sessions not to move the access arm with the vascular access and providing optional stands clipped to the dialysis chair arm rest. Devices provided to

participants by the study will be placed in a fresh, transparent plastic cover for each session to prevent nosocomial transmission of infections.

6.1.3. IVR Implementation

The IVR system will facilitate collection of patient-reported data, provision of treatment skill information, facilitation of skill practice, and provision of coach feedback. Participants will interact with the IVR system using a touch-tone telephone (smart or other cell phone or land line). Participants will use their telephone keypad to enter data or listen to pre-recorded messages. Coaches will log into a web-based portal where the participant's IVR data will be summarized and displayed to facilitate provision of feedback. Coaches will use the IVR system to deliver the feedback in the form of a voice message that participants can retrieve during a scheduled IVR assessment call. Alternatively, participants may initiate a call into the system using the system toll free telephone number.

6.1.4. Structure and Content of PCST Telehealth Intervention (Weeks 1 - 12)

The intervention has been designed to reduce pain interference, and address opioid use, symptoms of depression, anxiety and sleep disturbance, as appropriate. Skills are from the cognitive behavioral therapy family of interventions, including acceptance and commitment therapy, motivational interviewing and mindfulness (**see Table 6.1**).

General Structure of Sessions (45 minutes):

1. Check in (5 minutes)
2. Agenda/Homework review (5 minutes)
3. Key content (10 minutes)
4. Experiential practice (15 minutes)
5. Motivational interviewing (5 minutes)
6. Homework assignment (5 minutes)

Table 6.1. PCST Telehealth Sessions

Session	Key skills	Experiential Activity	Assigned Homework
1	Socialization to skills training;<br>Thinking about pain	Patient narrative	Pain questionnaire
2	Strategies to manage pain;<br>Mindfulness of opioid cues	Guided imagery	Pain tracking
3	Relaxation training	Progressive muscle relaxation & diaphragmatic breathing	Relaxation practice log
4	Exercise and pacing;<br>MI - Introduction	Activity pacing	Relaxation practice log<br>Activity pacing
5	Pleasant activities 1: Identification of activities;<br>MI - Opioid reduction	ACT Values/Goal setting<br>Activity scheduling	Activity scheduling
6	Pleasant activities 2: Implementation of activities;<br>MI - Opioid reduction/Goal acquisition	Activity scheduling	Activity scheduling

Session	Key skills	Experiential Activity	Assigned Homework
**7**	Cognitive coping 1;<br>MI – Opioid reduction/Goal acquisition	Thought/feeling/behavior connectome	Thought log 1
**8**	Cognitive Coping 2;<br>MI – Opioid reduction/Goal acquisition	Skills to challenge one's thinking style	Thought log 2
**9**	Problem solving to minimize pain interference;<br>IVR introduction;<br>MI – Opioid reduction/Goal acquisition	Problem solving exercise	Sleep diary
**10**	Sleep health, IVR feedback;<br>MI - Opioid reduction/Goal acquisition	Sleep Hygiene	Sleep diary
**11**	Anxiety management skills;<br>MI - Opioid reduction/Goal acquisition	Worry control	Anxiety tracking
**12**	Relapse prevention;<br>IVR hand-off;<br>MI - Opioid reduction/Goal acquisition	Highlights	

Abbreviations: MI, motivational interviewing; IVR, interactive voice response; ACT, acceptance and commitment therapy

6.1.5. Structure and Content of IVR Booster (Weeks 13-24)

Following 12 weeks of coach-led pain coping skills training, participants will receive an IVR booster intervention for an additional 12 weeks. The booster intervention will promote continued practice of the skills taught in the pain coping skills training with the goal of maintaining or improving gains made during the initial 12 weeks of the intervention.

6.1.5.1. Daily IVR Calls

Participants will receive daily IVR assessments of self-efficacy, pain intensity, pain interference, sleep quality, sleep duration, stress, and anxiety and weekly assessment of opioid use, values-based goal attainment, and pain coping skill practice for the duration of the 12-week booster treatment to provide ongoing feedback to the coach. Patient-reported data collected on the IVR assessment calls will form the basis for the coach's weekly feedback, but in distinction to the trial-wide CATI assessments, the IVR data will not be used as outcomes. Participants will receive automated calls daily for the duration of the booster treatment. After listening to their weekly coach feedback, participants may leave a message that their coach will access during regular business hours.

6.1.5.2. Didactic Skill Review

Participants will be able to access a verbal review of the pain management skills they learned during the telehealth component of the pain coping skills training. Skill review recordings will be brief (1-4 minutes) and can be accessed at any time. The skill review recordings will include:

- Explanation of pain model
- Deep breathing
- Progressive muscle relaxation
- Pacing

- Values-based goals
- Pleasant activities
- Relationship between thoughts, feeling, and behavior
- Challenging unhelpful thoughts
- Healthy sleep
- Anxiety
- Relapse prevention and problem solving

6.1.5.3. Guided Practice Sessions of Pain Coping Skills

Participants will be able to access the pre-recorded voice of a coach guiding them through behavioral rehearsals of the coping skills taught during the telehealth portion of the pain coping skills training. Coping skills will include:

- Deep breathing
- Progressive muscle relaxation
- Pacing
- Setting values-based goals
- Scheduling pleasant activities
- Identifying and countering unhelpful thoughts
- Problem solving
- Healthy sleep
- Managing anxiety

Additional skills not presented in telehealth CBT

- Mindfulness meditation

6.1.5.4. Values-based Goal

Participants will learn the basics of values-based goal setting in the telehealth portion of the pain coping skills training. Using the "value-based goal setting" guided practice module participants can set a goal for themselves. If they choose this option, the participant will report their goal using the audio recording function during the automated IVR daily call. One week later the participant will be asked to report on the degree to which they made progress toward this goal via the IVR system. This information will be made available to their coach and incorporated into their weekly feedback message.

6.1.5.5. Coach-Provided Feedback Message

Coaches will use data collected from daily IVR assessment calls to inform a 2-4 minute personalized feedback message for each participant. The message will be pre-recorded and left on the final day of each treatment week. Feedback will be guided by scripts that provide step-by-step instructions for feedback. Procedures for developing feedback scripts, feedback script examples, and suggestions for common patient circumstances have been developed in prior trials that have used IVR. Feedback is personalized based on participant's IVR

reports with insight into possible relationships between use of coping skills, anxiety, stress, and pain levels, suggestions for new pain management coping skills other than medication, and reminders about underutilized coping skills learned in the telehealth component of PCST.

6.1.6. Qualifications and Training of the PCST Coaches

PCST coaches will have masters-level training in a healthcare service delivery field with prior coursework and clinical experience in coaching patients with regard to health behaviors. The coaches will have comfort and familiarity in working remotely with people with chronic medical conditions. For both telehealth and IVR, the coaches will be trained in administration of the components of the intervention before the trial begins, and will be supervised during the trial as detailed in the **Manual of Procedures.**

For the telehealth component of the intervention, following the completion of training, each coach will be required to complete mock complete telehealth sessions prior to implementing trial sessions. These mock sessions will be video/audio recorded and will be reviewed using a structured fidelity adherence form.

Training of coaches for the IVR component of the intervention will include a review of the intervention manual, review of IVR data and feedback creation for practice patients followed by feedback and discussion with a seasoned IVR practitioner, and supervision on their active cases. IVR coaches will work with a trainer with experience providing pain coping skills training and delivering IVR-based treatments. Coaches will review example IVR feedback scripts and listen to audio recordings of examples of IVR feedback. The coaches will be given practice data and asked to generate six monthly feedback scripts using the script templates provided in the coach manual. Feedback scripts will be reviewed by the trainer one at a time with written or verbal feedback before the coach proceeds to generating the next feedback script. Additional practice may be assigned based on the judgment of the trainer until the coach is cleared to deliver the intervention to study participants. After clearance, the trainer will review feedback scripts. The training sessions for telehealth and IVR will be video recorded and used to supplement the training of coaches who join the study team after the trial is underway.

6.2. Buprenorphine Intervention

6.2.1. Overview

At Week 24, participants who meet the eligibility criteria for the buprenorphine intervention will be provided with individualized buprenorphine treatment recommendations and encouraged to initiate treatment; however, they will not be required to initiate buprenorphine. Participants who agree to switch to buprenorphine will sign an informed consent document. Individualized buprenorphine treatment recommendations will be made by the study buprenorphine physician based on current opioid use and other relevant factors.

6.2.2. Eligibility for Buprenorphine Intervention: Inclusion Criteria

Participants must meet the following inclusion criteria at Week 24 to be eligible for the Phase 2 buprenorphine intervention:

1. Current use of prescribed opioids at an average dosage of ≥ 20 MME/day
2. For participants of childbearing potential, a negative serum pregnancy test and willingness to use an effective form of contraception or remain abstinent from sexual intercourse with partners of the opposite sex during the buprenorphine administration period. See **Section 6.2.3.1** for the definition of childbearing potential and effective forms of contraception.

6.2.3. Eligibility for Buprenorphine Intervention: Exclusion Criteria

1. Current active addiction to substances other than tobacco, defined as probable moderate-severe substance use disorder for which evidence-based addiction treatment is indicated. This criterion may be identified by any of the following:
    - Patient self-report on eligibility interview
    - Documentation of probable substance use disorder during initial 24 weeks of study
    - Probable substance use disorder based on available clinical information and standardized assessment using the TAPS tool[59]
2. ALT, AST, or total bilirubin >2X the upper limit of normal based on the most recent available laboratory studies obtained within the preceding 12 weeks.
3. History of long QT syndrome, family history of long QT syndrome, or use of Class IA or Class III anti-arrhythmic medications. Class 1A anti-arrhythmic medications used in the outpatient setting include quinidine, procainamide, and disopyramide. Class III anti-arrhythmic medications include amiodarone, sotalol, dofetilide, and dronedarone.
4. Known allergy or prior intolerance to buprenorphine
5. Current use of buprenorphine

6.2.3.1. Childbearing Potential

A person of childbearing potential has experienced menarche and has not undergone successful sterilization or is not postmenopausal. Postmenopausal is defined as amenorrhea for ≥ 12 consecutive months without another cause.

Acceptable forms of contraception include:
- Intrauterine device or hormonal contraceptive implant
- Consistent use of hormonal contraception (depot medroxyprogesterone, combined oral contraceptive; contraceptive patch or ring)
- Consistent use of a barrier method with spermicide (diaphragm, condom, cervical cap)
- Partner who has undergone surgical sterilization
- Abstinence from sexual intercourse

6.2.4. Buprenorphine Formulations

Multiple buprenorphine formulations are FDA-approved for either pain or OUD. Formulations approved for pain (buccal and transdermal buprenorphine) are generally lower strength than those approved for OUD.

Because patients enrolled in this study are expected to be using opioids at a wide range of dosages, more than one buprenorphine formulation will be included as treatment options: sublingual buprenorphine/naloxone and buccal buprenorphine.

6.2.4.1. Sublingual Buprenorphine/Naloxone (Suboxone or Generic Equivalent)

Sublingual buprenorphine/naloxone (Suboxone and generic equivalents) is approved for opioid dependence/OUD. Naloxone is included to deter abuse of the medication by injection, but is inactive when the medication is taken sublingually as directed. Advantages of sublingual buprenorphine/naloxone over buccal (or transdermal buprenorphine) include wide availability with multiple generic products, relatively low cost, ability to efficiently titrate to higher dosages as needed for patients with a high degree of opioid tolerance/dependence, and widespread prescriber experience with this formulation.

6.2.4.2. Buccal Buprenorphine (Belbuca)

Buccal buprenorphine (Belbuca) is approved for moderate to severe chronic pain requiring around-the-clock opioid therapy. Advantages include low starting dosages. Disadvantages include relatively low upper dosages, higher cost, limited insurance coverage, and the time-consuming recommended approach to initiation for patients on high baseline opioid daily doses.

For the trial, buccal buprenorphine will be used for participants on full agonist opioids of <60 MME/day at the time of buprenorphine initiation, those who are anticipated to have low daily buprenorphine dosage requirements, or those who have other reasons for buccal buprenorphine to be preferred. Sublingual buprenorphine/naloxone will be the preferred formulation for participants on ≥60 MME/day at the time of buprenorphine initiation, those who are thought to have higher daily dosage requirements, or those with other reasons for sublingual buprenorphine to be preferred, such as insurance coverage anticipated to restrict access to alternatives after trial participation ends. In determining the initial medication selection and subsequent medication adjustments, individual clinical factors will be considered together with drug cost and factors that would affect availability to the participant after trial participation is completed.

6.2.5. Buprenorphine Regulatory Issues

With few exceptions, federal regulations prohibit clinicians from prescribing opioids for the purpose of treating OUD. One exception is for sublingual buprenorphine, which is FDA-approved for the treatment of OUD. Physicians and advanced practice providers may complete specific training and apply for a waiver through the DEA that authorizes them to prescribe sublingual buprenorphine for OUD. Importantly for this trial, the DEA clarified in 2004 that clinicians may prescribe sublingual buprenorphine “off-label” without a waiver. Use of sublingual buprenorphine in this protocol is for physical dependence due to prescribed long-term opioid therapy for pain, not for OUD; therefore, study prescribers are not required to have a waiver regardless of which buprenorphine formulation is prescribed.

6.2.6. Buprenorphine Prescribing and Dispensing

Each enrolling site will designate at least two study physicians as buprenorphine prescribers. Study physicians will prescribe buprenorphine for participants who are eligible for and elect to switch to buprenorphine after obtaining informed consent specifically for buprenorphine. Buprenorphine prescriptions written by authorized study prescribers will be transmitted to the central investigational pharmacy and the medication will be mailed by the investigational pharmacy to the participant in specified intervals throughout the study period. Participants will use study-provided mailers to return medication containers to the investigational pharmacy for dose counting and return of unused drug. For participants enrolled from the Veterans Affairs (VA) Healthcare System sites, all of the activities described above will be completed by the local VA pharmacy for each site.

6.2.7. Buprenorphine Initiation

Decisions about how to initiate buprenorphine will be individualized based on the participant's needs and preferences as well as the prescriber's experience and local resources. Participants may initiate buprenorphine at home or in the study physician's office. There are two methods for switching from full-agonist opioids to either buccal or sublingual buprenorphine: a traditional method and a newer crossover titration method.[60] The approach used for individual participants will be at the discretion of the study physician prescribing the buprenorphine; however, in general, the crossover titration method is preferred, and required for participants using long-acting opioid medications.

6.2.7.1. Traditional Method for Initiating Buprenorphine

For the traditional method, full-agonist opioids are stopped entirely prior to starting buprenorphine. The interval between last dose of the full-agonist opioid and the first dose of buprenorphine depends on the duration of action of the full-agonist opioid. In most cases, 12-18 hours is appropriate for short-acting opioids. For long-acting opioids, a longer interval is typically needed (approximately 18-24 hours for morphine SA). Patients using the traditional method for switching to buprenorphine will be advised that they will experience opioid withdrawal symptoms during the interval after stopping full-agonist opioids and before starting buprenorphine. For participants using long-acting opioids (e.g., oxycodone controlled-release, morphine controlled-release tablet or capsule, oxycodone controlled-release, oxymorphone extended-release hydromorphone extended-release, fentanyl transdermal), the crossover method will be used rather than the traditional method in order to avoid the need for prolonged periods without use of either the full agonist opioid or buprenorphine.

6.2.7.2. Crossover Titration Method for Initiating Buprenorphine

For the crossover titration method, buprenorphine is started at very low doses several days before the full-agonist opioid is stopped. Buprenorphine is titrated up over several (typically 3-5) days and the full-agonist opioid is discontinued once the buprenorphine is at a therapeutic dose. Case series demonstrate that the crossover titration method is well-tolerated,[60] and clinical experience indicates that some patients on long-term opioid therapy for pain prefer this approach because they do not have to experience opioid withdrawal

symptoms before initiating buprenorphine. Belbuca will be used as the buprenorphine formulation for the crossover titration method. Once the participant has up-titrated the Belbuca and discontinued the full agonist opioid, either Belbuca or buprenorphine/naloxone will be used as the maintenance buprenorphine formulation based on the considerations described in **Section 6.2.4**.

6.2.7.3. Information for Participants About Initiation of Buprenorphine

Precipitated opioid withdrawal is the main potential adverse effect of switching to buprenorphine; however, this can be avoided by adhering to the initiation protocol. When using the traditional method for switching to buprenorphine, the most critical point patients need to understand is that the full agonist opioids must be sufficiently cleared from the body before buprenorphine initiation. This means that participants must wait for the onset of at least moderate withdrawal symptoms. When using the crossover titration method, the critical point is to start at a very low dose and increase according to instructions over several days. All patients will be provided detailed written and verbal information about opioid withdrawal symptoms and about precipitated withdrawal. Understanding will be confirmed by teach-back.

6.2.7.4. Initial Visit with Buprenorphine Prescriber

Participants who meet eligibility criteria for the Phase 2 buprenorphine intervention will be scheduled as soon as convenient for a visit (in-person or by video) with a study physician who will prescribe the buprenorphine. Substance use testing using saliva will be performed prior to the initial visit with the buprenorphine prescriber.

The initial buprenorphine visit will be an in-person or video-based visit with the study buprenorphine prescriber, as described above. First visit tasks include the following:

- Obtain history of prescription and non-prescription opioid use and substance (alcohol, drug) use and assess for substance use disorder; assess buprenorphine risk factors (including risk for pregnancy if applicable)
- Provide standardized buprenorphine educational materials and resources
- Discuss benefits and risks of buprenorphine use and provide instructions to reduce risks such as dental problems
- Recommend buprenorphine and use shared decision-making to establish a plan for buprenorphine initiation and follow up; provide information about who should be contacted if problems or concerns occur between visits
- Complete informed consent process for initiating buprenorphine

Buprenorphine administration will not occur at the initial buprenorphine visit. After the initial visit has been completed and written informed consent is obtained, the study physician will prescribe buprenorphine, which will be mailed to the patient’s home. Patients will self-administer buprenorphine, typically at home, as described above.

Following buprenorphine initiation, structured follow-up will occur at multiple time points within the first 3 weeks after buprenorphine initiation. These follow-up visits will occur at 2-3 days, 1 week, 2 weeks, and 3 weeks after initiation, but the timing can be modified based on the judgement of the buprenorphine

prescriber. These visits will be conducted by the buprenorphine-prescribing study physician. Most visits will be conducted by telephone, but they may be conducted in-person or by video if preferred. After these four structured visits, follow up will occur at a frequency determined by the buprenorphine prescriber but will include follow-up at Weeks 32 and 36 during which plans for post-trial pain management will be discussed (see Section 6.2.9). Follow-up visit tasks include the following:

- Assess medication response and adverse effects, including withdrawal symptoms as appropriate
- Administer standard satisfaction questions
- Provide additional education or counseling as needed
- Perform substance use testing using saliva at the discretion of the buprenorphine prescriber in a manner consistent with standard clinical care
- Plan for post-trial pain management (Week 32 and Week 36 visits)

6.2.8. Buprenorphine Dosing Regimens

Dosages and dosing regimens for Belbuca and sublingual buprenorphine/naloxone are shown in **Table 6.2.** For participants using the crossover titration approach to transitioning to buprenorphine, Belbuca will be used to initiate the crossover titration. After the transition, Belbuca will be used for all participants who had been using a full agonist opioid at a dose of <30 MME/day. Belbuca will also be used for participants who had been using a full agonist opioid at a dose of 30-35 MME/day, unless the maximal dose of Belbuca (450 mcg twice daily) is insufficient to manage their symptoms. As detailed in the Buprenorphine Manual of Procedures, the dose of buprenorphine will be adjusted (increased or decreased) based on adequacy of pain control, withdrawal symptoms, and tolerability.

For participants using the traditional approach to transitioning to buprenorphine, either Belbuca or buprenorphine/naloxone can be used as the initial buprenorphine formulation depending on the full agonist opioid MME/day, in accordance with the guidelines in the Buprenorphine Manual of Procedures.

Table 6.2. Buprenorphine Dosing

Formulation	Formulation-Specific Exclusions[1]	Dosage Forms	Typical Dosing Regimens
Buprenorphine buccal film (Belbuca)	1. Use of full agonist opioid at dose >160 MME/day	• 75 mcg buccal film<br>• 150 mcg buccal film	75 mcg – 450 mcg two times per day
Buprenorphine/naloxone sublingual film (Suboxone or generic equivalent)	1. Use of full agonist opioid at dose <30 MME/day<br>2. Known hypersensitivity to naloxone	• 2 mg/0.5 mg sublingual film<br>• 8 mg/2 mg sublingual film	2 mg/0.5 mg – 8 mg/2 mg two to three times per day, not to exceed a total dose of 24 mg/6 mg per day

[1]These exclusions are in addition to the overall exclusion criteria for the buprenorphine intervention listed in **Section 6.2.3**. Note that these exclusions apply to the anticipated buprenorphine formulation that will be used immediately following the transition from the full agonist opioid. The exclusion does not apply to dose modifications required after a participant has been transitioned to buprenorphine.

6.2.9. Buprenorphine Maintenance During and After the Trial

Most participants should achieve a stable dosage of buprenorphine within a month of starting the medication. For either sublingual or buccal buprenorphine, dosing may be 2 to 3 times per day. Decisions about buprenorphine maintenance during and after the study will be made on an individual basis. Factors that will be considered include outcomes of therapy and participant preferences. Options for participants during and after completion of study participation include the following:

- Continue buprenorphine indefinitely;
- Taper off buprenorphine and completely discontinue opioids; or
- Rotate back to a full-agonist opioid at a lower dose. If participants rotate back to a full-agonist opioid after achieving stability on buprenorphine, the full-agonist opioid will be restarted at a lower dose to account for incomplete cross-tolerance.

6.2.10. Monitoring for Changes in Concomitant Medications During Treatment with Buprenorphine with Particular Attention to Medications that Affect Cyp3A4 Enzyme Activity

For patients receiving treatment with buprenorphine as a study intervention, in addition to the medication review that takes place every 4 weeks as a component of the regular interactions with the research team (see **Sections 7.4.2 and 7.5.2**), participants will be asked to contact the research team at any time that there is a change in their concomitant medications including initiations, discontinuations, or dose changes. The research team will review all medication changes that are identified either through scheduled interactions or through notifications by participants, to determine whether there have been changes in medications that affect (either increase or decrease) the activity of the Cyp3A4 enzyme. The research teams will be provided with lists of medications that affect Cyp3A4 activity to facilitate the review.

If there are changes to medications that affect Cyp3A4 activity, the research team will inquire about the following symptoms that might occur as a result of Cyp3A4-associated changes in in buprenorphine blood concentrations: increased pain, withdrawal symptoms, sedation, or respiratory depression. The initial 24 – 72 hours following the medication change will be of particular interest. Participants will be instructed to report symptoms to the research team. If symptoms occur, the dose of buprenorphine can be increased or decreased by the study buprenorphine prescriber as needed.

6.3. Usual Care

Participants in the Usual Care arm will be provided with written educational material about chronic pain and opioid medications, and available resources for treatment. Information will also be provided to the participant's treating clinicians that describes the trial and indicates the participant's randomized assignment. The same participant and prescriber informational materials will be used for the PCST intervention. Participants randomized to Usual Care will be assessed for eligibility for the Buprenorphine intervention at Week 24 using the same approach as for participants randomized to the PCST intervention.

6.4. Intervention Fidelity

Throughout the duration of the trial, the fidelity of the interventions will be monitored through ongoing review of both participant adherence and research team implementation. Efforts to maximize adherence will be implemented by the Clinical Center research teams with guidance and assistance from the trial's Quality Control Committee.

For the coach-led telehealth component of the PCST intervention, adherence will be based on the number of telehealth sessions completed (out of 12 prescribed sessions) and categorized as minimal (< 3 session), some (3-9 sessions), and most (10-12). Additional adherence data will include the duration of sessions, completion of PCST homework, utilization of symptom management skills, and utilization of opioid reduction strategies. For the IVR component of the PCST intervention, the proportion of calls completed and duration of IVR will be indicators of adherence.

To ensure that the PCST intervention is delivered as intended there will be regular interactions between coaches and the relevant supervisory team, during which implementation and clinical issues will be reviewed. For each coach, the audio recordings of all PCST sessions for a minimum of one participant will be reviewed to provide ratings for treatment fidelity using forms that capture the core skills and intervention components. In addition to the review of all sessions for a minimum of one participant per coach, a random sample of subsequent telehealth sessions will be reviewed and scored for fidelity to the intervention. This will allow coaches to receive support and real-time feedback to ensure the highest standards for delivering the intervention. Ongoing supervision for the IVR component will also be provided during weekly meetings. Enactment fidelity, i.e., the degree to which participants implement and use the intervention skills, will be assessed post-treatment by the coach using a checklist.

For the buprenorphine intervention, participants who meet eligibility criteria will be encouraged, but not required, to initiate buprenorphine. The proportion of participants offered the drug who initiate buprenorphine is one of the exploratory outcomes for the intervention. Rates of completion of the structured follow-up contacts and subsequent monthly contacts will serve as indicators of implementation fidelity.

6.5. Clinical Management of Trial Participants

6.5.1. Concomitant Therapy

The following approach will be taken regarding concomitant therapies:

1. Doses and frequency of use of non-opioid analgesics, antidepressants, anxiolytic and sedatives/hypnotics will be recorded at baseline and Weeks 4, 8, 12, 16, 20, 24, and 36.
2. Use of non-study behavioral therapy or any other non-pharmacological therapy for pain (such as acupuncture, physical therapy, etc.) will be recorded at baseline and Weeks 4, 8, 12, 16, 20, 24, and 36.
3. With the exception of the buprenorphine intervention, opioid medications will not be prescribed or managed by the study.

6.5.2. Opioid Adherence Problems

For participants who are not receiving the buprenorphine intervention, opioid adherence problems will be managed by the participant's clinical care providers. During Weeks 24 – 36, for participants receiving the buprenorphine intervention, the study physician prescribing the buprenorphine will be responsible for addressing buprenorphine or opioid adherence problems. If issues are identified, they will be addressed with the goals of improving safety. An individualized monitoring plan will be developed to aid in diagnosing and addressing the underlying cause of the behavior. This plan may include: a) education; b) medication adjustment; c) additional study visits; d) review of state Prescription Drug Monitoring Program database; e) blood or saliva drug testing; f) shorter prescription intervals; or g) referral to a mental health or addiction clinician.

6.5.3. Clinical Management of OUD or other Substance Use Disorders

Previously undiagnosed moderate-severe OUD or other substance use disorders may become clinically apparent during the course of the trial. Participants with symptoms or signs of OUD or other substance use disorders will be evaluated by a study clinician with appropriate expertise or referred to appropriate local non-study clinicians to establish a diagnosis. Study clinicians and coordinators will facilitate access to evidence-based addiction treatment for participants who are diagnosed with OUD or other substance use disorder during the study.

Participants who are diagnosed with OUD or other substance use disorders after randomization should not be withdrawn from the study and may continue to receive treatment in the PCST or Usual Care group if the interventions are compatible with the care they receive for the substance use disorder. The buprenorphine intervention is not intended as medication treatment for OUD. If a participant receiving the Phase 2 buprenorphine intervention is diagnosed with moderate-severe OUD, study clinicians should facilitate transfer of buprenorphine prescribing to a DEA-certified prescriber of medications for OUD and the patient's participation in the buprenorphine intervention should be discontinued. However, the participant will continue to be followed for data collection and outcome ascertainment.

6.5.4. Clinical Management of Suicidality

It is possible that suicidal ideation will be evident from responses to question #9 of the PHQ-9 survey used during eligibility screening or during ascertainment of the patient reported outcomes throughout the course of trial participation. Participants will be considered to have possible elevated risk of suicide based on a response of "1", "2", or "3" to the PHQ-9 Question 9: Over the last 2 weeks how often have you been bothered by thoughts that you would be better off dead or of hurting yourself in some way: 0 = not at all, 1 = several days, 2 = more than half the days, or 3 = nearly every day.

If during eligibility screening, based on the response to the PHQ-9 Question 9, a participant is considered to a possible elevated risk of suicide, the research coordinator will administer two specific follow-up questions about suicidal ideation. If the responses to these questions support possible suicidal intent the research coordinator will stop the screening procedures and contact a designated member of the enrolling site's

research team to perform an immediate further assessment of suicidal intent. The patient will be provided with appropriate support and resources, including arrangement for emergency care and/or referral to mental health specialists, as needed, based on the assessment by the designated research team member. The research team will inform the participant's treating nephrologist and primary care provider about the events that transpired, and will document the follow-up procedures. If the research team deems that the participant is safe to proceed with screening procedures and possible randomization into the trial, the screening process will be resumed.

Management of elevated risk of suicide identified during the PRO ascertainments will be addressed using a similar approach. The personnel at the centralized PRO assessment center (CATI Center) will have emergency telephone numbers for 3 members of the research team at each enrolling site. If a participant indicates increased risk of suicide based on a response of "1", "2", or "3" to question 9 of the PHQ-9 survey, the CATI team member will ask two specific follow-up questions (the same questions used during screening). If the responses to the two follow-up questions support possible suicidal intent the CATI team member will inform the participant that the research team will contact the participant to provide follow-up and any necessary resources and will end the call. The CATI team will immediately contact the research team at the participant's enrolling site to inform them of the participant's elevated suicide risk. A designated person on the research team will immediately contact the participant, assess the participant's level of possible suicidal ideation, and, based on the results, provide appropriate support and resources, including arrangement for emergency care and/or referral to mental health specialists as needed. The enrolling site's research team will inform the participant's treating nephrologist and primary care provider about the events that transpired. The administration of the remaining PRO questionnaires will be deferred until a later date after the management of suicidal intent has been completed. The CATI team member will notify the SDRC about the event electronically using the participant's study ID. The research team will document the follow-up procedures.

7. STUDY PROCEDURES AND ASSESSMENTS

A schedule of study visits and procedures is provided in **Appendix 14.1**. To the extent possible, in-person study visits will take place at the dialysis unit during scheduled dialysis sessions.

7.1. Pre-screening and Screening

The pre-screening and screening activities include:

1. Pre-screening
2. Obtaining informed consent for those who meet pre-screening criteria
3. Performing screening activities to determining eligibility

Patients at participating dialysis units will be pre-screened for chronic pain as described in **Section 5.4.** Those who meet the criteria for Screening will be approached in person to determine interest in trial participation. Study personnel will discuss the study goals and procedures with the potential participant and assess understanding of the content in the consent form before obtaining informed consent from the participant. The consenting process will be performed by a qualified investigator or study site designee. Informed consent will be obtained and documented before any Screening procedures are performed.

Eligibility will be determined through interaction with the participant, review of medical records, and interaction with treating clinicians. Review of the inclusion and exclusion criteria will be performed and documented in the data management system for all consented participants, including those who are found to be ineligible.

7.2. Baseline Data Collection and PRO Ascertainment – (within 4 weeks of Screening)

Baseline data includes demographic information, medical history via checklist, dialysis history, substance use history, pain history, prescribed and over-the-counter medications, and use of behavioral or other non-pharmacologic interventions for pain.

PRO questionnaires will be administered by telephone by a centralized CATI team (see **Section 4.2.3**). The order of preference for the day of the week for completing CATI, from optimal to the least preferred, is as follows:

1. The day following the first dialysis treatment of the week: Tuesday for patients on a Monday-Wednesday-Friday schedule, or Wednesday for patients on a Tuesday-Thursday- Saturday schedule, or Monday for patients on a Sunday-Tuesday-Thursday schedule. The first dialysis treatment of the week is defined as the treatment following the 72-hour dialytic interval.
2. The day following the second dialysis treatment of the week: Thursday for patients on a Monday-Wednesday-Friday schedule, or Friday for patients on a Tuesday-Thursday-Saturday schedule, or Wednesday for patients on a Sunday-Tuesday-Thursday schedule.
3. Preferably, within the first hour of the 2nd dialysis session of the week which is Wednesday for patients on a Monday-Wednesday-Friday schedule, or Thursday for patients on a Tuesday-Thursday-Saturday schedule, or Tuesday for patients on a Sunday-Tuesday-Thursday schedule.

To the extent possible, the timing with respect to dialysis will be kept consistent throughout the duration of the trial.

If all attempts to complete a follow up CATI call are unsuccessful, the Clinical Center Principal Investigator may authorize the site study coordinator to complete the PROs using paper forms chairside during dialysis. This approach is expected to be used only under exceptional circumstances.

The baseline PRO questionnaires include*:

- Brief Pain Inventory 7-item Interference (BPI-Interference)
- Brief Pain Inventory 4-item Severity (BPI-Severity)
- Pain Catastrophizing Scale (PCS) SF6
- Opioid Use Timeline Follow-Back (TLFB)
- Pain Mapping Questionnaire
- Single Item Quality of Life Scale [SIS] from the McGill Quality of Life (MQOL) Questionnaire
- Patient Health Questionnaire (PHQ-9)
- Generalized Anxiety Disorder (GAD-7)
- Coping Strategies Questionnaire 24 (CSQ-24)
- Single item targeting self-efficacy for pain
- PROMIS Self-Efficacy for Managing Chronic Conditions – Managing Symptoms – Short Form 8A
- PROMIS Sleep Disturbance 6a + Sleep duration
- PROMIS-Fatigue SF 6a
- PROMIS Physical Functioning SF 6b
- Multidimensional Scale of Perceived Social Support (MSPSS)
- Dialysis Symptom Index
- PROMIS Satisfaction with Social Roles and Activities
- Everyday Discrimination Scale

*Citations for PRO references can be found in **Appendix 14.2.**

7.3. Randomization for Phase 1 (within 1 week of Baseline)

Randomization to the Phase 1 treatment group will take place after determination of eligibility, baseline data collection, and baseline PRO ascertainment. Randomization will be performed via the centralized Data Management System. Recruitment of new participants will conclude once 640 participants have been randomized. At the time of the 640th randomization, participants who are “in the pipeline” (participants who provided consent and have been screened eligible but have not yet been randomized) will also be randomized and continue in the study.

7.4. Phase 1 Intervention Administration, Data Collection, and Research Team Contacts (Weeks 1 - 24)

7.4.1. Intervention Administration (Weeks 1 - 24)

The PCST and Usual Care interventions will be administered during Weeks 1 - 24 as described in **Sections 6.1, 6.2, and 6.4**, respectively.

7.4.2. Contacts with the Research Team (Weeks 4, 8, 12, 16, and 20)

Participants in both randomized groups will be contacted by research coordinators either in person or by telephone every 4 weeks during Phase 1. These contacts will be used for the following:

- Ascertainment of hospitalizations (secondary outcome)
- Ascertainment of falls (secondary outcome)
- Ascertainment of Adverse Events of Interest and SAEs
- Documentation of any change in the participant's opioid prescriber(s)
- Documentation of non-opioid analgesics, antidepressants, anxiolytic and sedatives/hypnotics
- Review of concomitant medications with documentation of initiation of new medications or discontinuation of prior medications
- Documentation of use of non-study behavioral or other non-pharmacologic treatments for pain
- Scheduling of Week 12 or Week 24 CATI calls

7.4.3. Follow-Up PROs (Week 12)

Follow-up PRO questionnaires will be administered by telephone by a centralized CATI team. To the extent possible, the surveys should be performed using the same timing with respect to dialysis as was used for the Baseline PROs. The Week 12 PRO ascertainment will provide the data for the primary outcome.

The follow-up PRO questionnaires performed at Week 12:

- Brief Pain Inventory 7-item Interference (BPI-Interference) – primary outcome
- Brief Pain Inventory 4-item Severity (BPI-Severity)
- Pain Catastrophizing Scale (PCS) SF6
- Opioid Use Timeline Follow-Back (TLFB)
- Single Item Quality of Life Scale [SIS] from the McGill Quality of Life (MQOL) Questionnaire
- Patient Health Questionnaire (PHQ-9)
- Generalized Anxiety Disorder (GAD-7)
- Coping Strategies Questionnaire (CSQ) 1-item
- Single item targeting self-efficacy for pain
- PROMIS Self-Efficacy for Managing Chronic Conditions – Managing Symptoms – Short Form 8A
- PROMIS Sleep Disturbance 6a + Sleep duration
- PROMIS-Fatigue SF 6a
- PROMIS Physical Functioning SF 6b
- Multidimensional Scale of Perceived Social Support (MSPSS)
- Dialysis Symptom Index

- Patient Global Impression of Change (PGIC)
- PROMIS Satisfaction with Social Roles and Activities

7.4.4. End of Phase 1 Intervention, Week 24 PROs, and Determination of Eligibility for Buprenorphine (Week 24)

Follow-up PRO questionnaires will be administered by telephone by a centralized CATI team. To the extent possible, the surveys should be performed using the same timing with respect to dialysis as was used for the Baseline PROs.

The follow-up PRO questionnaires performed at Week 24 include:

- Brief Pain Inventory 7-item Interference (BPI-Interference)
- Brief Pain Inventory 4-item Severity (BPI-Severity)
- Pain Catastrophizing Scale (PCS) SF6
- Opioid Use Timeline Follow-Back (TLFB)
- Single Item Quality of Life Scale [SIS] from the McGill Quality of Life (MQOL) Questionnaire
- Patient Health Questionnaire (PHQ-9)
- Generalized Anxiety Disorder (GAD-7)
- Coping Strategies Questionnaire (CSQ) 1-item
- Single item targeting self-efficacy for pain
- PROMIS Self-Efficacy for Managing Chronic Conditions – Managing Symptoms – Short Form 8A
- PROMIS Sleep Disturbance 6a + Sleep duration
- PROMIS-Fatigue SF 6a
- PROMIS Physical Functioning SF 6b
- Multidimensional Scale of Perceived Social Support (MSPSS)
- Dialysis Symptom Index
- Patient Global Impression of Change (PGIC)
- PROMIS Satisfaction with Social Roles and Activities
- Internalized Stigma in Chronic Pain Scale

After the Week 24 PROs have been ascertained, participants in the 2 randomized groups will proceed to Phase 2. Participants in the PCST group will be told that the Phase 1 intervention has been completed.

All participants in both randomized groups will undergo eligibility determination for the buprenorphine intervention (**see Sections 6.3.2 and 6.3.3** for the Buprenorphine Inclusion and Exclusion Criteria).

7.5. Phase 2 Intervention Administration, Data Collection, and Research Team Contacts (Weeks 24 - 36)

7.5.1. Intervention Administration (Weeks 24 - 36)

Participants who are eligible for buprenorphine will receive recommendations and instructions for switching from their full agonist opioid medication to buprenorphine. Written informed consent will be required before initiating buprenorphine. The buprenorphine will be administered as detailed in **Sections 6.3.4 – 6.3.8**.

7.5.2. Contacts with the Research Team (Weeks 28, 32, and 36)

All participants will be contacted by research coordinators either in person or by telephone every 4 weeks during Phase 2. These contacts will be used for the following:

- Ascertainment of hospitalizations (secondary outcome)
- Ascertainment of falls (secondary outcome)
- Ascertainment of Adverse Events of Interest and SAEs
- Documentation of any change in the participant's opioid prescriber(s)
- Documentation of non-opioid analgesics, antidepressants, anxiolytic and sedatives/hypnotics
- Review of concomitant medications with documentation of initiation of new medications or discontinuation of prior medications. For participants being treated with buprenorphine as a trial intervention, medications will also be reviewed for initiation, discontinuation, or change in dose for medications that increase or decrease Cyp3A4 enzyme activity (see **Section 6.2.10** for additional information about monitoring for changes in medications with effects on Cyp3A4).
- Documentation of use of non-study behavioral or other non-pharmacologic treatments for pain
- Scheduling of Week 36 CATI call

7.5.3. Planning for End-of-Trial (Weeks 32 - 36)

For participants taking buprenorphine, during Weeks 32 - 36, the study buprenorphine prescriber will develop a plan for continued use or discontinuation of buprenorphine with the participant and their usual opioid prescriber after the trial ends (**see Section 6.2.8** for post-trial options).

7.5.4. Follow-Up PROs (Week 36)

Follow-up PRO questionnaires will be administered by telephone by a centralized CATI team at Week 36. To the extent possible, the surveys should be performed using the same timing with respect to dialysis as was used for the Baseline PRO.

The follow-up PRO questionnaires performed at Week 36 include:

- Brief Pain Inventory 7-item Interference (BPI-Interference)
- Brief Pain Inventory 4-item Severity (BPI-Severity)
- Pain Catastrophizing Scale (PCS) SF6
- Opioid Use Timeline Follow-Back (TLFB)

- Single Item Quality of Life Scale [SIS] from the McGill Quality of Life (MQOL) Questionnaire
- Patient Health Questionnaire (PHQ-9)
- Generalized Anxiety Disorder (GAD-7)
- Coping Strategies Questionnaire (CSQ) 1-item
- Single item targeting self-efficacy for pain
- PROMIS Self-Efficacy for Managing Chronic Conditions – Managing Symptoms – Short Form 8A
- PROMIS Sleep Disturbance 6a + Sleep duration
- PROMIS-Fatigue SF 6a
- PROMIS Physical Functioning SF 6b
- Multidimensional Scale of Perceived Social Support (MSPSS)
- Dialysis Symptom Index
- Patient Global Impression of Change (PGIC)
- PROMIS Satisfaction with Social Roles and Activities
- Everyday Discrimination Scale

7.5.5. End of Phase 2 / End of Study (Week 36)

Trial participation will end at Week 36. For participants taking buprenorphine, continuation or transition off buprenorphine will be handled as described in **Sections 6.3.8 and 7.6.3**.

7.5.6. Buprenorphine Qualitative Interviews (After Study Participation has been Completed)

Approximately 25 participants who were offered the Phase 2 buprenorphine intervention will be asked to complete a one-time qualitative interview by telephone. The purpose of the interviews is to understand why participants were willing or were not willing to switch from their full agonist opioids to buprenorphine when it was offered to them as part of the HOPE Trial. The interviews will be approximately 30 minutes in duration and will be conducted by researchers at the University of Pittsburgh. All interviews will be audio-recorded and transcribed for analysis. The audio recordings will be destroyed after all analyses are completed. Informed consent will be obtained prior to performing the interviews using an informed consent form that is specific to the buprenorphine qualitative interviews. The consent will be obtained under a waiver of consent documentation since not all participants will be able to complete the consent process in person. Participants will have concluded all other elements of study participation before being contacted for possible participation in this interview.

8. DISCONTINUATION OF STUDY INTERVENTIONS AND STUDY PARTICIPATION

8.1. Discontinuation of Study Interventions

For all of the trial interventions, participants will be encouraged to continue for the duration indicated by the protocol, but they will have the right to discontinue at any time based on preference. Interruptions in the PCST intervention may be necessary because of hospitalizations or other inter-current events; if so, the intervention will be resumed as soon as it is possible based on the judgement of the research team. Every effort will be made to continue buprenorphine during hospitalizations just as would be done in clinical care. As with any medication, buprenorphine may induce intolerable side effects, or, in rare cases, allergic reactions that will necessitate its discontinuation by the study physician. In these cases, the study physician will use clinical judgment to decide whether the participant should be switched back to full agonist opioids or discontinued from opioids altogether. Participants who switch back to full agonist opioids will remain in the buprenorphine arm. The study buprenorphine prescriber will be responsible for ensuring that the participant has continued access to pain medication either through their clinical opioid prescriber or through the study buprenorphine prescriber.

Participants who are not willing or able to continue trial interventions will be encouraged to remain in the trial and continue data collection, adverse event monitoring, and outcome ascertainment.

8.2. Participant Withdrawal / Discontinuation from the Study

Participants will be free to withdraw from participation in the study at any time. If a participant indicates a desire to withdraw, the research team will discuss the request with the participant and determine whether the participant is willing to allow ongoing data collection for outcome ascertainment or adverse event monitoring. An investigator may discontinue or withdraw a participant from the study if any clinical adverse event, laboratory abnormality, or other medical condition or situation occurs such that continued participation in the study would not be in the best interest of the participant. The reason for participant withdrawal or discontinuation from the study will be recorded on a Withdrawal case report form.

9. ADVERSE EVENT ASCERTAINMENT AND REPORTING

9.1. Definitions

Definitions are per the January 2007 Guidance on Reviewing and Reporting Unanticipated Problems Involving Risks to Participants or Others and Adverse Events, Office on Human Research Protection (OHRP) Guidance. **http://www.hhs.gov/ohrp/policy/advevntguid.html**

9.1.1. Adverse Event

An *adverse event (AE)* is any untoward or unfavorable medical occurrence in a human study participant, including any abnormal sign (for example, abnormal physical examination or laboratory finding), symptom, or disease, temporally associated with the participant's involvement in the research, whether or not considered related to the participant's participation in the research.

9.1.2. Serious Adverse Event

A *serious adverse event* (SAE*)* is any AE that is:

- fatal or results in death
- life-threatening
- requires or prolongs hospital stay
- results in persistent or significant disability or incapacity
- results in congenital anomalies or birth defects
- an important medical event*

*Important medical events are those that may not be immediately life threatening but are clearly of major clinical significance.

9.1.3. Unanticipated Problem Involving Risk to Participants or Others

An Unanticipated Problem is any incident, experience, or outcome that meets all of the following criteria:

- It is unexpected (in terms of nature, severity, or frequency) given the research procedures that are described in the IRB-approved research protocol and informed consent document and the characteristics of the participant population being studied;
- It is related or possibly related to participation in the research; possibly related means that there is a reasonable possibility that the incident, experience or outcome may have been caused by the procedures involved in the research, and
- It suggests that the research places participants or others at a greater risk of harm (including physical, psychological, economic, or social harm) related to the research than was previously known or recognized.

9.1.4. Pre-Existing Condition

A pre-existing condition is one that is present at the start of the study. A pre-existing condition will be recorded as an adverse event if the frequency, intensity, or the character of the condition worsens during the study period.

9.1.5. Adverse Event Reporting Period

The study period during which adverse events must be tracked and reported is defined as the period from the initiation of study procedures to study completion. Participants who undergo screening and are ineligible for the trial will no longer be followed for adverse events. If a participant is rescreened, adverse event monitoring/reporting will resume and continue until the end of trial participation or until he/she is determined to be ineligible for the trial.

9.1.5.1. Post-Study Adverse Events

All unresolved adverse events will be followed by the investigator until the events are resolved, the participant is lost to follow-up, or the adverse event is otherwise explained. At the last scheduled visit, the investigator will instruct each participant to report any subsequent event(s) that the participant, or the participant's personal physician, believes might reasonably be related to participation in this study. The investigator will notify the SDRC of any death or adverse event occurring after a participant has discontinued or terminated study participation that may reasonably be related to the study.

9.2. Adverse Events of Interest

The following adverse events are anticipated in patients with chronic pain, opioid use, or treatment with the trial interventions, and will be considered AEs of Interest.

- Development of suicidality – based on response to question 9 of the PHQ-9
- Development of opioid use disorder or other substance use disorder
- Adverse effect from a medication started during the trial (including buprenorphine) to treat pain, opioid dependence, depression, or anxiety
- Falls
- Withdrawal symptoms
- Dental events in participants who have switched to buprenorphine

AEs of Interest will be ascertained during the contacts between the research coordinator and the participant that occur every 4 weeks through specific questioning and, as appropriate, by review of medical records. Information about the events will be recorded on the Adverse Event of Interest case report form (CRF) and entered into the data management system.

9.3. Anticipated SAEs

The following serious adverse events are anticipated in the hemodialysis population and are not considered Unanticipated Problems. Note that the designation as "Anticipated" does *not* imply that the event is not an SAE but relates to the regulatory definition of Unanticipated Problems as provided in **Section 9.1.3**.

- Death
- Coronary Ischemia including:
  - Unstable angina
  - Acute MI
  - Coronary revascularization
- Heart failure hospitalization or exacerbation
- Cardiac arrest
- Cardiac arrhythmia (ventricular or atrial)
- Peripheral vascular revascularization
- Amputation
- Hypotension
- Hypertension
- Nausea or vomiting
- Vascular Access Events including:
  - Catheter exchange, removal, or thrombolysis
  - Arteriovenous graft or fistula complications
    - Thrombosis
    - Stenosis
    - Revascularization
    - Infection
- Infections including:
  - Pneumonia
  - Bacteremia
  - Hemodialysis vascular access infection

9.4. Reporting of AEs of Interest, SAEs, and Unanticipated Problems

9.4.1. Reporting AEs of Interest

AEs of Interest will be documented on CRFs and entered into the Data Management System as they are ascertained. If the Adverse Event of Interest meets the criteria for an SAE it will be also documented on an SAE form and reported to the SDRC using the same approach as for any other SAE.

9.4.2. Reporting SAEs

SAEs will be reported to the SDRC within 1 business day of first knowledge of the event. An SAE form will be completed by the Clinical Center research team and sent by electronic mail to the SDRC. At the time of the initial report, the following information will be provided:

- Study identifier
- Study Center
- Participant number
- A description of the event
- Date of onset
- Current status
- Whether study treatment was discontinued
- The reason why the event is classified as serious
- Investigator assessment of the association between the event and study participation

Additional information will be provided on a follow-up SAE form and mailed electronically to the SDRC typically within 7 calendar days of first knowledge of the SAE. Significant new information on ongoing serious adverse events will be provided promptly to the SDRC until the event is resolved (i.e., no longer ongoing). SAE forms can be completed by research coordinators but must be signed by a Clinical Center investigator.

If a participant becomes pregnant while receiving buprenorphine, this will be reported as an SAE. Pregnancy outcomes will be collected, including the outcome of the infant and whether the pregnancy was terminated. This information will be submitted to the IRB.

SAEs that are still ongoing at the end of the trial participation will be followed to determine the final outcome.

The SDRC will facilitate the timely medical review and reporting of SAEs, and provide reports to the NIDDK and the Data and Safety Monitoring Board (DSMB) in accordance with DSMB-approved study policies and regulatory requirements (see **Section 11.5.3** for information about the DSMB).

9.4.3. Reporting Unanticipated Problems

Unanticipated problems will be reported by the Clinical Center to the SDRC within one calendar day of first knowledge of the event. Additional information will be provided to the SDRC as it becomes available. The SDRC will inform the NIDDK and the DSMB about Unanticipated Problems and will report the events to the IRB as described in **Section 9.5**.

9.5. Reporting to the IRB

The University of Pennsylvania (UPenn) Institutional Review Board is the Single IRB of Record for the trial for all sites other than the VA sites. The VA Central Institutional Review Board (CIRB) will serve as the IRB of Record for VA sites. The SDRC will report events that occur at either the Clinical Centers or the SDRC to the UPenn IRB in accordance with the reporting requirements of the IRB shown in Table 9.1. The VA sites will be responsible for reporting to the VA CIRB.

Table 9.1. Reporting to the Single IRB of Record

Relatedness	Expectedness	Reportable to IRB?	Timeframe for Reporting
Unrelated or Unlikely related	Expected or Unexpected	No report	N/A
Possibly, Probably, or Definitely related	Expected	No report	N/A
Possibly related	Unexpected	Yes (includes Serious and Non-Serious), *only if* the event suggests that the research placed participants at greater risk than was previously known or recognized (i.e., changes to the study conduct are required to mitigate risk and/or participants' willingness to participate may be adversely impacted)	Expedited reporting within 10 business days and summarized at Continuing Review
Probably or Definitely related; No death	Unexpected	Yes (includes Serious and Non-Serious)	Expedited reporting within 10 business days and summarized at Continuing Review
Probably or Definitely related death	Unexpected	Yes	Expedited reporting within 3 calendar days and summarized at Continuing Review

9.5.1. Reporting Process

Unanticipated problems will be reported using the appropriate IRB-designated form or as a written report of the event (including a description of the event with information regarding its fulfillment of the above criteria, follow-up/resolution and need for revision to consent form and/or other study documentation).

Copies of each report and documentation of IRB notification and receipt will be maintained by the SDRC and in the Clinical Center's study file.

9.5.2. Other Reportable Events

- Any adverse event that would cause the study's Steering Committee to modify the protocol or informed consent form or would prompt other action by the IRB to assure protection of human participants.
- Information that indicates a change to the risks or potential benefits of the research, in terms of severity or frequency.
- Breach of confidentiality
- Change to the protocol made without prior IRB review to eliminate apparent immediate hazard to a research participant.
- Incarceration of a participant when the research was not previously approved under 45 CFR part 46 subpart C and the investigator believes it is in the best interest of the participant to remain in the study.

- Complaint of a participant when the complaint indicates unexpected risks or the complaint cannot be resolved by the research team.
- Protocol violation (meaning an accidental or unintentional deviation from the IRB approved protocol) that in the opinion of the investigator placed one or more participants at increased risk, or affects the rights or welfare of participants.

9.5.3. SDRC Notifications to Participating Investigators

The SDRC will notify the Contact Principal Investigator at each Clinical Center, in a written safety report, of any event from any of the Clinical Centers or SDRC that meets the criteria for reporting to the Single IRB of Record.

10. STATISTICAL CONSIDERATIONS

10.1. Endpoints, Objectives, and Trial Questions

Scientific questions that will be addressed with the trial design, and the relevant comparison groups, outcomes, and analytic approaches are depicted in **Table 10.1**.

- The primary endpoint is based on a continuous outcome, the BPI Interference score. The endpoint for the primary analyses is based on the BPI Interference score at Week 12 (see **Section 4.2.4** for the rationale for the Week 12 time point).
- The primary objective is to determine whether initial component of the PCST intervention, which consists of 12 weekly, live, coach-led sessions, reduces pain interference for hemodialysis-dependent patients with chronic pain. The null hypothesis is that changes in the BPI Interference score from baseline to 12 weeks are the same for the PCST and Usual Care groups. The alternative hypothesis is that changes in the BPI Interference score from baseline to 12 weeks differ between the randomized groups.
- Secondary endpoints include pain severity, pain catastrophizing, opioid use, other PROs that capture conditions associated with chronic pain, and clinical outcomes.
- A secondary objective is to determine whether the full PCST intervention, which consists of weekly, live, coach-led sessions for 12 weeks followed by IVR for 12 weeks, reduces pain interference for hemodialysis-dependent patients with chronic pain. The null hypothesis is that changes in BPI Interference scores between baseline and 24 weeks are the same for the PCST and Usual Care groups. The alternative hypothesis is that changes in the BPI Interference scores between baseline and 24 weeks are different between the randomized groups.
- An exploratory objective is to learn about the acceptability, tolerability, and efficacy of buprenorphine by evaluating the proportion of participants who initiate buprenorphine among those who are offered buprenorphine, the proportion of participants who do not require discontinuation of buprenorphine because of adverse effects among those who initiate buprenorphine, and the change in BPI-Interference from Week 24 to Week 36 among those who initiate buprenorphine. For this exploratory objective, the purpose is to generate preliminary estimates and signals.

Table 10.1. Scientific Questions that can be Addressed with the Trial Design

Scientific Question	Contrast of Interest	Outcome(s)	Analytic Approach
1. Does PCST consisting of weekly, live, coach-led sessions improve outcomes at 12 weeks?<br>This is the primary trial question.	PCST vs Usual Care; baseline to Week 12	• Primary: BPI Interference<br>• Secondary: pain severity, pain catastrophizing, opioid use, other PROs, and clinical outcomes	• Linear mixed effects models for continuous outcomes;<br>• Generalized linear mixed effects models for binary outcomes;<br>• Time to event models accounting for semi-competing risk of death (where relevant)
2. Does PCST consisting of weekly, live, coach-led sessions followed by IVR improve outcomes at 24 weeks?<br>This is a secondary question and findings will be interpreted as such.	PCST vs Usual Care; baseline to Week 24	• Primary: BPI Interference<br>• Secondary: pain severity, pain catastrophizing, opioid use, other PROs, and clinical outcomes	• Linear mixed effects models for continuous outcomes;<br>• Generalized linear mixed effects models for binary outcomes;<br>• Time to event models accounting for semi-competing risk of death (where relevant)
3. Are the effects of PCST sustained?<br>This is a secondary question and findings will be interpreted as such.	PCST vs Usual Care; baseline to Week 36	• Primary: BPI Interference<br>• Secondary: pain severity, pain catastrophizing, opioid use, other PROs, and clinical outcomes	• Linear mixed effects models for continuous outcomes;<br>• Generalized linear mixed effects models for binary outcomes;<br>• Time to event models accounting for semi-competing risk of death (where relevant)
**Exploratory Questions Addressed with the Phase 2 Intervention**			
4. Is buprenorphine acceptable to patients?	Single group	• % Initiating buprenorphine<br>• Reasons for non-initiation of buprenorphine	Summary statistics: frequency, percentage
5. How well tolerated is buprenorphine?	Single group	• % who do not discontinue buprenorphine due to adverse effects<br>• Adverse effects	Summary statistics: frequency and percentage
6. Does buprenorphine improve outcomes for patients using full agonist opioids for pain?	Single group	• BPI Interference<br>• Opioid use, pain severity, pain catastrophizing, other PROs	• Linear mixed effects models for continuous outcomes;<br>• Generalized linear mixed effects models for binary outcomes;

10.2. Statistical Considerations for the Phase 1 Randomization

10.2.1. Sample Size and Power Analysis for the Primary Objective

The primary objective for Phase 1 is to compare changes from baseline to 12 weeks in the BPI Interference scores between the PCST and Usual Care groups. The null hypothesis is that the change in BPI Interference score is the same in the PCST and Usual Care groups.

Table 10.2 depicts the minimum detectable difference between the PCST and Usual Care groups in the change in BPI Interference score, for a power of 90%, a type 1 error of 5%, an attrition rate of 20%, standard deviations of change of 2 and 3, a total number of participants ranging from 480 – 800 (participant numbers per Clinical Center ranging from 60 to 100), intra-class correlation coefficients of 0 and 0.02, and an interim analysis of one look at 50% information time. Details of the interim analysis are described in **Section 10.2.3**. All calculations are based on two-sample t tests. As one example, if the total sample size is 640, the number of participants per Clinical Center is 80, the ICC is 0 (i.e., no clustering effect), and the standard deviation of the change in BPI Interference score is 2, the BPI Interference score for PCST would need to decrease by at least 0.62 points if the change in BPI Interference score for Usual Care were 0 (**Table 10.2**, **Row #2).** As another example, again using the parameters in **Table 10.2, Row #2**, the change in the BPI Interference score would need to decrease by 1.02 in the PCST group if the score decreased by 0.40 in the Usual Care group.

Table 10.2. Minimum Detectable Differences between the PCST and Usual Care Groups in the Change from Baseline to Week 12 in the BPI Interference Score

Row	Total N	Clinical Center n	ICC	Minimum Detectable Between-Group Difference in Change in BPI Interference Score[1]	
				If SD = 2	If SD = 3
1	800	100	0	0.56	0.83
2	640	80	0	0.62	0.93
3	480	60	0	0.72	1.08
4	800	80	0.02	0.73	1.10
5	640	80	0.02	0.78	1.17
6	480	60	0.02	0.86	1.29

[1]Effect sizes determined for a power of 90%, at a type 1 error of 5%, an attrition rate of 20%, standard deviations of change of 2 and 3, and an interim analysis of one look at 50% information time, using two-sample t tests.

Each of eight Clinical Centers aims to recruit "m" participants. The University of Washington Clinical Center consists of three recruitment sites: University of Washington, Rogosin Institute, and University of New Mexico. The University of Washington and Rogosin will each recruit 43.75% of the Clinical Center's target, and the University of New Mexico will recruit 12.5% of the target. The University of Pittsburgh Clinical Center consists of two recruitment sites: University of Pittsburgh and University of Pennsylvania. The University of Pittsburgh site will recruit two thirds of the Clinical Center's target and the University of Pennsylvania site will recruit one third of the target. The Vanderbilt Clinical Center consists of two recruitment sites: Vanderbilt University Medical Center and West Virginia University. Vanderbilt University Medical Center will recruit two thirds of the

Clinical Center's target, and West Virginia University will recruit one third of the target. The Yale University Clinical Center consists of three recruitment sites in Veterans Affairs Healthcare Systems; each site will recruit one third of the target. Therefore, there are 14 recruitment sites. When clustering at the recruitment site level is considered in the power calculation, the average cluster size per recruitment site per arm is $\bar{m} = 0.571 \times \frac{m}{2} = 0.286m$, and the standard deviation of cluster size is $s_m = 0.313 \times \frac{m}{2} = 0.156m$. This will yield the design effect of $1 + \text{ICC} \times \left[\left\{\left(\frac{s_m}{\bar{m}}\right)^2 + 1\right\} \times \bar{m} - 1\right]$. For example, for $m = 80$ and $\text{ICC} = 0.02$, $\bar{m}$ is 22.88, s_m is 12.48, and the design effect is 1.574. Thus, the sample size per arm in the power calculation is around 139 $\left(\frac{80\times8}{2} \times \frac{1}{1.574} \times \{1 - 0.2\} \times \frac{1}{1.173} \cong 139\right)$ after taking into account the design effect (1.574), the attrition rate (0.2) and the inflation factor of the interim analysis (1.173).

10.2.1.1. Rationale for the Selected Sample Size

The trial will aim to enroll 640 participants. Based on the minimum detectable differences in changes of BPI Interference scores under a range of assumptions provided in **Table 10.2**, for the primary objective of comparing PCST with Usual Care in Phase 1, this sample size will allow detection of a difference in the change in BPI Interference score of 0.62 if there is no intra-cluster correlation and a standard deviation of change of 2, or 1.17. under the conservative assumptions of an intra-cluster correlation coefficient (ICC) of 0.02 and a standard deviation of change of 3 (which is larger than the SD of change observed in studies of chronic pain in non-ESRD patient populations[57]). Based on a review of the literature, the Initiative on Methods, Measurement, and Pain Assessment in Clinical Trials identified 1 as the minimal clinically important change *for individuals* on the BPI Interference scale.[57] However, this group also recognized a critical tenet of randomized trials with between group comparisons: "…criteria for clinically important change in individuals cannot be directly applied to the evaluation of clinically important group differences." Determination of the importance of group differences must be established in the broader context of the disease being treated, the currently available treatments, and the overall risk-benefit evaluation of the treatment. Our proposed sample size will allow us to detect a between-group difference *as small as* the ranges listed above. We hypothesize that the actual observed difference will be larger because we are testing an adaptation of an evidence-based intervention that has had moderate effect sizes in other populations and settings.[27-29]

10.2.2. Statistical Analyses

General Approach. The following descriptive statistics will be used: number of observations, mean, standard deviation, median, minimum and maximum for continuous variables; frequencies and proportions for categorical variables; number of events and rates for count variables. Results will be reported in accordance with the extended CONSORT guidelines for pragmatic clinical trials[61] using two-sided statistical tests and confidences intervals. For all analyses, the overall level of significance will be set at 0.05. Methods for multiple comparisons such as Bonferonni, false discovery, or others, will be implemented to maintain the overall 0.05 level of significance. All analyses will be adjusted for site and the presence or absence of opioid use using the criteria provided in **Section 5.3**. All analyses will be performed using SAS v9.4 (SAS Institute, Cary, NC) or the latest version of R (currently 3.6.1; https://cran.r-project.org/).

Baseline Descriptive Statistics. Randomization adequacy will be assessed by comparing the distributions of baseline demographic and clinical characteristics between the PCST and Usual Care groups. Comparability for continuous variables will be examined graphically and by summary statistics: mean, standard deviation, median, minimum, and maximum for continuous variables, and frequency and proportion for and categorical variables. If statistically and/or clinically meaningful differences are found in baseline characteristics (i.e., baseline characteristics are not balanced between the PCST and Usual Care groups), sensitivity analyses will be conducted with covariate adjustment.

10.2.2.1. Analyses of the Primary Endpoint for the Phase 1 Randomization

Question 1 (Table 10.1): Does a PCST intervention consisting of weekly, live, coach-led sessions improve outcomes at 12 weeks?

Question 1 addresses the primary trial hypothesis. The primary efficacy endpoint, the change in BPI Interference between baseline and 12 weeks, and will be analyzed using a linear mixed effects model.[62] The linear mixed effects model will include fixed effects of intervention, time, and interactions between intervention and time, taking into account the repeated measures over time and clustering of recruitment sites via random effects. All repeated measures of the BPI Interference scores at baseline and 12 weeks will be included in the model as the dependent variable. The linear mixed effects model is expressed as

$$y_{ijk} = \beta_0 + \beta_1 X_{ij} + \beta_2 I(T_{ijk} = 12) + \beta_3 X_{ij} I(T_{ijk} = 12) + \boldsymbol{\gamma}^{\boldsymbol{T}} \boldsymbol{W}_{ij} + \theta_i + \alpha_{ij} + \varepsilon_{ijk},$$

where y_{ijk} is the BPI Interference score for participant *ij* (*i* = 1, 2, …, 14; *j* = 1, 2, …, n_i; n_i is number of participants from recruitment site *i*) at time point *k* (*k* = 1, 2), X_{ij} is the binary intervention indicator ($X_{ij} = 1$ if PCST; $X_{ij} = 0$ if Usual Care) for participant *ij*, T_{ijk} is the study weeks for participant *ij* at time point *k* ($T_{ij1} = 0$ and $T_{ij2} = 12$), $I(\cdot)$ is an indicator function returning 1 (0) if the event is true (false), $\boldsymbol{W}_{ij}$ is a vector of baseline measures, including the stratification factors, for participant *ij*, β's and $\boldsymbol{\gamma}$ are regression parameters for the fixed effects, θ_i and α_{ij} are the regression parameters for the random effects for recruitment site *i* and participant *ij*, respectively, and ε_{ijk} is the random error for participant *ij* at time point *k*. Model diagnostics will be conducted to examine the assumption of normality. If there are potential violation of the assumptions, transformations of the data beginning with a log transformation will be explored. The primary efficacy estimand is the contrast of PCST and Usual Care for the change at the 12-week time point; i.e., β_3. Differences in changes, their 95% confidence intervals, and p-values will be presented.

Question 2 (Table 10.1): Does a PCST intervention consisting of weekly, live, coach-led sessions for 12 weeks followed by IVR for 12 weeks improve outcomes at 24 weeks?

To answer this question, the aforementioned linear mixed effects model is revised by adding the BPI Interference score at 24 weeks to the dependent variable. The linear mixed effects model is expressed as

$$y_{ijk} = \beta'_0 + \beta'_1 X_{ij} + \beta'_2 I(T_{ijk} = 12) + \beta'_3 I(T_{ijk} = 24) + \beta'_4 X_{ij} I(T_{ijk} = 12) + \beta'_5 X_{ij} I(T_{ijk} = 24) + \boldsymbol{\gamma}'^{\boldsymbol{T}} \boldsymbol{W}_{ij} + \theta'_i + \alpha'_{ij} + \varepsilon'_{ijk},$$

where y_{ijk} is the BPI Interference score for participant *ij* at time point *k* (*k* = 1, 2, 3) and $T_{ij3} = 24$. The estimand for **Question 2** in **Table 10.1** is the contrast of PCST and Usual Care for the change at the 24-week time point; i.e.,β'_5. Differences in changes, their 95% confidence intervals, and p-values will be presented.

Question 3 (Table 10.1): Are the effects of PCST sustained?

To answer this question, the aforementioned linear mixed effects model is revised by adding the BPI Interference score at 36 weeks to the dependent variable. The linear mixed effects model is expressed as $y_{ijk} = \beta_0'' + \beta_1'' X_{ij} + \beta_2'' I(T_{ijk} = 12) + \beta_3'' I(T_{ijk} = 24) + \beta_4'' I(T_{ijk} = 36) + \beta_5'' X_{ij} I(T_{ijk} = 12) + \beta_6'' X_{ij} I(T_{ijk} = 24) + \beta_7'' X_{ij} I(T_{ijk} = 36) + \boldsymbol{\gamma}''^{\boldsymbol{T}} \boldsymbol{W}_{ij} + \eta'' V_{ij} I(T_{ijk} \geq 24) + \delta'' Z_{ij} I(T_{ijk} \geq 24) + \theta_i'' + \alpha_{ij}'' + \varepsilon_{ijk}''$,
where y_{ijk} is the BPI Interference score for participant *ij* at time point *k* (*k* = 1, 2, 3, 4), $T_{ij4} = 36$, V_{ij} is the binary buprenorphine eligibility indicator ($V_{ij} = 1$ if buprenorphine eligible; $V_{ij} = 0$ if not buprenorphine eligible), Z_{ij} is the binary buprenorphine indicator ($Z_{ij} = 1$ if initiated buprenorphine; $Z_{ij} = 0$ if declined buprenorphine), and η'' and δ'' are regression parameters for the fixed effects. The estimand for **Question 3** in **Table 10.1** is the contrast of PCST and Usual Care for the change at the 36-week time point; i.e.,β_7''. Differences in changes, their 95% confidence intervals, and p-values will be presented.

10.2.3.2 Analyses of the Secondary Endpoints

The secondary endpoints include pain severity, pain catastrophizing, opioid use, other PROs, hospitalizations, falls and mortality.

For continuous endpoints, such as pain severity, pain catastrophizing, and opioid use expressed as MME/day., similar methods as described for the primary outcome will be used to compare differences in changes of the endpoints between PCST and Usual Care at 12 (**Table 10.1; Question 1**), 24 (**Table 10.1; Question 2**), and 36 (**Table 10.1; Question 3**) weeks; i.e., linear mixed effects models with adjustment for baseline measures (and additional adjustment for buprenorphine eligibility and initiation of buprenorphine for **Question 3** in **Table 10.1**), taking into account the repeated measures over time and clustering of recruitment sites via random effects.

For binary endpoints, such as the composite of pain interference and opioid use, generalized linear mixed effects models with a binomial distribution and a log link adjusting for baseline measures (additionally adjusting for buprenorphine eligibility and initiation of buprenorphine for **Question 3** in **Table 10.1**), taking into account the repeated measures over time and clustering of recruitment sites via random effects, will be used to compare relative risks between PCST and Usual Care at 12 (**Table 10.1; Question 1**), 24 (**Table 10.1; Question 2**), and 36 (**Table 10.1; Question 3**) weeks.

For recurrent endpoints (counts) such as number of hospitalizations and falls, participants will be followed from baseline to 36 weeks, loss-to-follow-up, or death, whichever comes first. Generalized linear mixed effects models with a Poisson distribution, an offset of follow-up time and a log link adjusting for baseline measures (additionally adjusting for buprenorphine eligibility and initiation of buprenorphine for **Question 3** in **Table 10.1**) will be used to compare event rates between PCST and Usual Care during the Phase 1 study period (**Table 10.1; Questions 1 – 3**). Repeated measures over time and clustering of recruitment sites will be taken into account via random effects.

For time to event endpoints such as mortality, participants will be followed from baseline to 36 weeks, loss-to-follow-up, or death, whichever comes first. Cox proportional hazards models will be used to compare risks of

all-cause mortality between PCST and Usual Care (**Table 10.1; Questions 1 – 3**), with the end of the 36-week study period and loss-to-follow-up treated as censoring events in the models. For time to event endpoints such as the first hospitalization (or fall), participants will be followed from baseline to 36 weeks, loss-to-follow-up, death or the first admission to hospital (or fall), whichever comes first. Cause-specific hazards models or sub-distribution hazards models will be used to compare risks of the first hospitalization (or fall) between PCST and Usual Care (**Table 10.1; Questions 1 – 3**) where death is a semi-competing event, and the end of the 36-week study period and loss-to-follow-up are censoring events. All proportional hazards models will adjust for baseline measures, time-dependent buprenorphine eligibility, and time-dependent initiation of buprenorphine and take into account clustering of recruitment sites via random effects (i.e., proportional frailty models). The assumption of proportionality for all proportional hazards models will be examined using Schoenfeld residuals plots. If the assumption of proportionality is violated, models that incorporate stratification or time dependent covariate-effects will be considered.

10.2.2.2. Subgroup Analyses

Potential effect modification will be examined by including interaction terms between interventions and baseline factors such as gender, race, age, coping style, depression, and anxiety. For baseline factors with statistically significant interactions, intervention effects will be reported by subgroups.

10.2.2.3. Missing Data

If missing data are unavoidable, they will be addressed by 1) using models that are valid under the missing at random assumption, and 2) performing sensitivity analyses. The generalized linear mixed effects models are valid under the missing at random assumption. The extent and patterns of missing data will be examined, and logistic regression models will be used to identify factors associated with the missingness. Several approaches will be conducted to address the missingness. First, the generalized linear mixed effects models will further adjust for the identified factors associated with the missingness, compensating for potential violation of the missing at random assumption. Second, the propensity of the missingness, calculated from the above logistic regression, can be used to create inverse weighting in the generalized linear mixed effects models. Sensitivity analyses under missing not at random assumptions (e.g. pattern mixture models) will be conducted to examine the robustness of model-based results.[63] For time to event analyses, competing risk models such as cause-specific hazards models or sub-distribution hazards models will be used when there are competing events (e.g., death).

10.2.3. Planned Interim Efficacy and Futility Analyses for the Phase 1 Randomization

Prior to trial completion, only the DSMB and a designated study statistician from the SDRC will have access to data with unblinded randomization assignments. During each interim look, the DSMB could recommend early trial termination due to efficacy or futility of the primary objective or any operational inadequacy as follows.

1. Efficacy: strong evidence is found for the primary hypothesis, indicating that a significant difference in changes in BPI Interference scores between PCST and Usual Care at Week 12 is likely to be concluded, resulting in a decision to stop the trial for superior efficacy.

2. Futility: weak evidence is found for the primary hypothesis, indicating a significant difference in changes in BPI Interference scores between PCST and Usual Care at Week 12 is unlikely to be concluded, resulting in a decision to stop for futility.
3. Operational inadequacy: failure to obtain success in the implementation of the trial such as slow recruitment, poor retention, improper data handling, or inability to implement the protocol.

For Phase 1 of the trial, one interim analysis is planned at 50% information time. However, the DSMB may request additional interim analyses and therefor a plan for an interim analysis with two looks at 50% and 75% information time is also provided. The 50% and 75% information times are defined as the times at which the randomized participants #320 and #480, respectively, have completed the Week 12 visit.

Interim Analysis with One Look at 50% Information Time. To accommodate the additional interim look, a spending-function approach[64] will be used to determine stopping bounds for efficacy and futility. For efficacy, an alpha-spending function is chosen to resemble the O'Brien-Fleming bounds.[65] For futility, a beta-spending function is chosen to select a bound that approximates a conditional power of 20% or below. Stopping boundaries are depicted in Table 10.3 for a power of 90% and a two-sided type 1 error of 5% for an interim analysis with one look at 50% information time. At the 50% information time, if the test statistic for the primary objective, i.e., regression coefficient β_4 divided by its standard error, is less than -2.89 (greater than -0.97), the trial will stop for efficacy (futility).

Table 10.3. Stopping Boundaries of Efficacy and Futility for a Power of 90% and at a Two-Sided Type 1 Error of 5% for an Interim Analysis with One Look at 50% Information Time

	Interim Analysis Look 1	Final Analysis
Information Time	50%	100%
Efficacy Boundary		
Test Statistic Critical Value	-2.89	-1.97
Conditional Power at the Boundary	99.9%	
Futility Boundary		
Test Statistic Critical Value	-0.97	-1.97
Conditional Power at the Boundary	20%	

gsDesign R package[66] used to calculate stopping boundary and conditional power

Interim Analysis with Two Looks at 50% and 75% Information Time. To accommodate a second interim look at 75% information time, stopping boundaries are depicted in Table 10.4 for a power of 90% and a two-sided type 1 error of 5% for an interim analysis with two looks at 50% and 75% information time. At the 50% information time, if the test statistic for the primary objective, i.e., regression coefficient β_4 divided by its standard error, is less than -2.89 (greater than -1.09), the trial will stop for efficacy (futility). At the 75% information time, if the test statistic is less than -2.50 (greater than -1.53), the trial will stop for efficacy (futility).

Table 10.4. Stopping Boundaries of Efficacy and Futility for a Power of 90% and at a Two-Sided Type 1 Error of 5% for an Interim Analysis with Two Looks at 50% and 75% Information Time

	Interim Analysis Look 1	Interim Analysis Look 2	Final Analysis
Information Time	50%	75%	100%
Efficacy Boundary			
Test Statistic Critical Value	-2.89	-2.50	-1.99
Conditional Power at the Boundary	99.9%	96.3%	
Futility Boundary			
Test Statistic Critical Value	-1.09	-1.53	-1.99
Conditional Power at the Boundary	20%	32%	

If the interim analysis with two looks at 50% and 75% information time is conducted, the minimum detectable differences depicted in Table 10.2 will be updated by replacing the inflation factor of 1.173 for the interim analysis with one look at 50% information time with the inflation factor of 1.277 for the interim analysis with two looks at 50% and 75% information time. Table 10.5 depicts the minimum detectable difference between the PCST and Usual Care groups in change in BPI Interference from baseline to 12 weeks, for a power of 90%, at a type 1 error of 5%, an attrition rate of 20%, standard deviations of change of 2 and 3, a total number of participants ranging from 480 – 800 (participant numbers per Clinical Center ranging from 60 to 100), intra-class correlation coefficients of 0 and 0.02, and an interim analysis of two looks at 50% and 75% information time.

Table 10.5. Minimum Detectable Differences between the PCST and Usual Care Groups in the Change from Baseline to Week 12 in the BPI Interference

Row	Total N	Clinical Center n	ICC	Minimum Detectable Between-Group Difference in Change in BPI Score[1]	
				If SD = 2	If SD = 3
1	800	100	0	0.58	0.87
2	640	80	0	0.65	0.97
3	480	60	0	0.75	1.13
4	800	100	0.02	0.76	1.14
5	640	80	0.02	0.82	1.22
6	480	60	0.02	0.90	1.35

[1]Effect sizes determined for power of 90%, type 1 error of 5%, attrition rate of 20%, standard deviations of change of 2 and 3, and interim analysis of two looks at 50% and 75% information time, using two-sample t tests.

10.3. Statistical Considerations for the Phase 2 Intervention

This section describes statistical considerations for the exploratory objective of evaluating acceptability, tolerability, and efficacy of buprenorphine in Phase 2 of the trial. Participants who, at Week 24, meet the buprenorphine eligibility criteria are included in the following exploratory analyses.

Due to the exploratory nature of Phase 2, potential numbers of participants initiating buprenorphine and declining buprenorphine are illustrated in **Table 10.6 – Table 10.8** ranging from 20 per group to 80 per group.

Although we cannot predict how many participants among the 300 patients using opioids at trial enrollment will be eligible for the Phase 2 intervention at 24 weeks, under plausible assumptions we estimate that there will be at least 40 who are on sufficiently high doses of opioids at 24 weeks to meet the eligibility criteria for the buprenorphine intervention.

10.3.1. Acceptability

Participants who meet buprenorphine eligibility criteria at 24 weeks will be encouraged to switch from their full agonist opioid to buprenorphine. The acceptability objective explores the percentage of participants who initiate buprenorphine (acceptability), and also examines reasons for non-willingness to initiate buprenorphine (non-acceptability) (**Table 10.1; Question 4**). Table 10.6 depicts 95% confidence intervals for percentages of participants who initiate a switch from a full agonist opioid to buprenorphine for a range of numbers of participants eligible for and offered buprenorphine, and a range of percentages of participants who initiate a switch to buprenorphine.

Table 10.6. 95% Confidence Intervals for % of Participants Willing to Switch from a Full Agonist Opioid to Buprenorphine

Offered Buprenorphine, N	Initiated Buprenorphine, N	Initiated Buprenorphine, %	95% Confidence Interval for Initiated Buprenorphine, %
20	5	25	(8.7, 49.1)
40	10	25	(12.7, 41.2)
60	15	25	(14.7, 37.9)
80	20	25	(16, 35.9)
20	10	50	(27.2, 72.8)
40	20	50	(33.8, 66.2)
60	30	50	(36.8, 63.2)
80	40	50	(38.6, 61.4)
20	15	75	(50.9, 91.3)
40	30	75	(58.8, 87.3)
60	45	75	(62.1, 85.3)
80	60	75	(64.1, 84)

10.3.2. Tolerability

Among the participants who are offered and agree to switch to buprenorphine, the tolerability outcome provides the number (percentage) of participants who do not discontinue buprenorphine due to adverse effects (**Table 10.1; Question 5**). For those who discontinue buprenorphine due to adverse effects, reasons for non-tolerability will be examined. **Table 10.7** depicts 95% confidence intervals for percentages of participants who continue buprenorphine until the end of the study for a range of numbers of participants willing to switch from a full agonist opioid to buprenorphine and a percentage of participants who continue of 60%.

Table 10.7. 95% Confidence Intervals for % of Participants who Continue Buprenorphine Until the End of the Study (Week 36)

Offered Buprenorphine, N	Initiated Buprenorphine, N	Continued Buprenorphine until Week 36, N	Continued Buprenorphine until Week 36, %	95% Confidence Interval for Continued Buprenorphine until Week 36, %
20	5	3	60	(14.7, 94.7)
40	10	6	60	(26.2, 87.8)
60	15	9	60	(32.3, 83.7)
80	20	12	60	(36.1, 80.9)
20	10	6	60	(26.2, 87.8)
40	20	12	60	(36.1, 80.9)
60	30	18	60	(40.6, 77.3)
80	40	24	60	(43.3, 75.1)
20	15	9	60	(32.3, 83.7)
40	30	18	60	(40.6, 77.3)
60	45	27	60	(44.3, 74.3)
80	60	36	60	(46.5, 72.4)

10.3.3. Efficacy

The exploratory efficacy endpoint is the primary efficacy endpoint used in Phase 1, the BPI Interference score, measured at 24 weeks and 36 weeks, where the baseline for Phase 2 is Week 24. The exploratory efficacy outcome (**Table 10.1; Question 7**) is the change in BPI Interference score between Week 24 and Week 36, and will be analyzed using a linear mixed effects model. The linear mixed effects model includes a fixed effect of time, taking into account the repeated measures over time via random effects. All repeated measures of the BPI Interference scores at Week 24 and Week 36 will be included in the model as the dependent variable. The linear mixed effects model is expressed as $y_{ij} = \beta_0 + \beta_1 I(T_{ij} = 36) + \boldsymbol{\gamma}^{\boldsymbol{T}} \boldsymbol{W}_{ij} + \alpha_i + \varepsilon_{ij}$, where y_{ij} is the BPI Interference score for participant *i* (*i* = 1, 2, …, *I; I* depends on the number of participants appropriate for buprenorphine and initiating buprenorphine at Week 24) at time point *j* (*j* = 1, 2), T_{ij} is the study weeks for participant *i* at time point *j* ($T_{i1} = 24$ and $T_{i2} = 36$), $I(\cdot)$ is an indicator function returning 1 (0) if the event is true (false), $\boldsymbol{W}_{ij}$ is a vector of measures for participant *i* at time point *j*, β_1 and $\boldsymbol{\gamma}$ are regression parameters for the fixed effects, α_i is the regression parameter for the random effect for participant *i*, and ε_{ij} is the random error for participant *i* at time point *j*. Model diagnostics will be conducted to examine the assumption of normality. If there are potential violation of the assumptions, transformations of the data beginning with a log transformation will be explored. The exploratory efficacy estimand is the contrast between Week 24 and Week 36 for the BPI Interference score; i.e., β_1. The change in BPI Interference score and its 95% confidence interval will be presented. Signals of efficacy will be estimated from the participants who are eligible for and switch to buprenorphine. Table 10.8 depicts 95% confidence intervals for the change in BPI Interference scores for a range of numbers of participants switching to buprenorphine, an attrition rate of 20%, and a standard deviation of change of 2.

Table 10.8. 95% Confidence Intervals for the Change in BPI Interference Score between Weeks 24 and 36

Offered Buprenorphine, N	Initiated Buprenorphine, N	95% Confidence Interval for Change in BPI Interference Score[1]
20	5	(-2.18, 4.18)
40	10	(-0.67, 2.67)
60	15	(-0.27, 2.27)
80	20	(-0.07, 2.07)

[1]Assumes a standard deviation of change of 2 and an attrition rate of 20%.

10.4. Safety Analyses

Proportions of participants with at least one adverse event and rates of adverse events will be determined. Differences between the PCST and Usual Care groups in the proportions and the rates will be compared using logistic regression models and Poisson regression models, respectively, adjusting for baseline measures. For participants who switch to buprenorphine, adverse events occurring during the buprenorphine phase (Weeks 24-36) will also be summarized.

10.5. Tabulation of Individual Participant Data

Data will be tabulated in a de-identified fashion, without center or patient identifiers.

10.6. Populations for Analyses

For questions related to the Phase 1 intervention , analyses will use an intent-to-treat (ITT) approach, in which all available data on all randomized participants are included. Participants who discontinue an assigned intervention will be encouraged to remain in the trial to provide complete follow-up data and will be included in the ITT analysis. For participants with missing outcome ascertainment due to withdrawal or other reasons, data from earlier assessments will be used. For example, for the primary objective for Phase 1, if Week 12 outcome data are not available, baseline data will be used.

The characteristics at the time of randomization for those participants without complete follow-up will be examined; however, there will be limited statistical power to detect differences between participants with and without complete follow-up. To assess potential biases introduced by differential withdrawal among the intervention groups, comparisons of withdrawal rates and/or time to withdrawal will be performed as an ancillary analysis to the primary efficacy analysis.

Other populations that may be used in secondary analyses are defined as follows:

- As-treated population: Any participant randomized into the study regardless of whether they received study intervention. As-treated participants will be analyzed according to the study intervention actually received.

- All-treated population: Any participant randomized into the study that received at least some of the study intervention.
- Per-protocol population: Any participant who was appropriately randomized and was exposed to the protocol-dictated study intervention for ≥ 75% of the planned duration and had the outcome assessments through Week 24.

11. REGULATORY, ETHICAL, AND OVERSIGHT CONSIDERATIONS

11.1. Informed Consent Process and Participant Compensation

Consent forms available in English and Spanish describing in detail the study interventions, study procedures, and risks will be provided to potential participants. Documentation of informed consent is required before any study procedures are conducted.

Informed consent is a process that is initiated before an individual agrees to participate in the study and continues throughout the individual’s study participation. Consent forms will be Institutional Review Board (IRB)-approved and the potential participant will be asked to read and review the document. The investigator or a member of the research team designated by the investigator will explain the research study to the potential participant and answer any questions that may arise. A verbal explanation will be provided in terms suited to the individual’s comprehension of the purposes, procedures, and potential risks of the study and of their rights as research participants. Potential participants will have the opportunity to carefully review the consent form and ask questions prior to signing. The teach-back method will be used to assess the potential participant’s understanding of the research study. Potential participants will have the opportunity to discuss the study with family members or surrogates, and to fully consider the decision to participate or not. Potential participants will be informed that participation is voluntary and that they may withdraw from the study at any time, without prejudice. A copy of the signed informed consent document will be given to participants for their future reference. The rights and welfare of the participants will be protected by emphasizing to them that the quality of their medical care will not be adversely affected if they decline to participate in this study.

Participants will be compensated for the time and effort required to complete study activities. Compensation will be linked to the successful completion of Research Team Contacts and Follow-Up PRO questionnaires administered by CATI teams. Compensation amount will be standardized across Clinical Centers; however, compensation delivery methods will be determined by each Clinical Center individually.

11.2. Study Discontinuation and Closure

This study may be temporarily suspended or prematurely terminated by the Sponsor if there is sufficient reasonable cause. Written notification, documenting the reason for study suspension or termination, will be provided by the suspending or terminating party to the Clinical Centers, SDRC, IRB, and the DSMB. If the study is prematurely terminated or suspended, the Clinical Center Principal Investigators will promptly inform study participants of the study status and of changes to their study visit schedule or procedures.

Circumstances that may warrant termination or suspension include, but are not limited to:

- Determination of unexpected, significant, or unacceptable risk to participants
- Insufficient recruitment that is not able to be improved adequately
- Insufficient adherence to protocol requirements
- Data that are not sufficiently complete and/or evaluable

The study may resume once concerns about safety, protocol compliance, and data quality are addressed to the satisfaction of the sponsor, DSMB, IRB, and investigators are satisfied.

In terminating the study, the Sponsor and the Principal Investigators will assure that adequate consideration is given to the protection of the interests of participants.

11.3. Confidentiality and Privacy

Information about study participants will be kept confidential and managed according to the requirements of the Health Insurance Portability and Accountability Act of 1996 (HIPAA). Those regulations require a signed participant authorization informing the participant of the following:

- What protected health information (PHI) will be collected from participants in this study
- Who will have access to that information and why
- Who will use or disclose that information
- The rights of a research participant to revoke their authorization for use of their PHI

If a participant revokes authorization to collect or use PHI, the investigator, by regulation, retains the ability to use all information collected prior to the revocation of participant authorization. For participants who have revoked authorization to collect or use PHI, attempts should be made to obtain permission to collect at least vital status (i.e. that the participant is alive) at the end of their scheduled study period.

Participants will be assigned a unique identifier at the time of enrollment that will be used for all data entered into the data management system. Clinical site personnel will de-identify all medical records before sending them to the SDRC by obliterating any Protected Health Information (PHI). Upon receipt, SDRC personnel will review the records to ensure that no PHI is visible.

11.3.1. Certificate of Confidentiality

To further protect the privacy of study participants, a Certificate of Confidentiality will be issued by the National Institutes of Health (NIH). This certificate protects identifiable research information from forced disclosure. It allows the investigator and others who have access to research records to refuse to disclose identifying information on research participation in any civil, criminal, administrative, legislative, or other proceeding, whether at the federal, state, or local level. By protecting researchers and institutions from being compelled to disclose information that would identify research participants, Certificates of Confidentiality help achieve the research objectives and promote participation in studies by helping assure confidentiality and privacy to participants.

11.4. Future Use of Data

Data collected for this study will be stored and analyzed at the University of Pennsylvania Scientific and Data Research Center. After the study is completed, de-identified, archived data will be transmitted to and stored at the NIH HEAL Data Repository and/or the NIDDK Central Repository for use by other researchers including those outside of the study. Information about sending data to these repositories and future use will be included in the informed consent.

11.5. Safety Oversight

11.5.1. Clinical Center Medical Monitoring

Each Clinical Center Principal Investigator will be responsible for overseeing the safety of the study at his/her site. This safety monitoring will include careful assessment and appropriate reporting of adverse events as described in **Section 9**, as well as the construction and implementation of a site data and safety-monitoring plan. Medical monitoring will include a regular assessment of the number and type of serious adverse events.

11.5.2. Internal Safety Committee

A Safety Committee comprised of investigators from the Clinical Centers and the SDRC, research coordinators from the Clinical Centers, and Project Managers from the SDRC will review reports generated each month by the SDRC that include SAEs and AEs of Interest. Deaths will be reported by the SDRC to the Safety Committee when the SDRC becomes aware of their occurrence. The Safety Committee Chair will decide whether an ad hoc meeting of the Safety Committee is needed to review a death prior to the regularly scheduled monthly meeting.

11.5.3. Independent Data and Safety Monitoring Board (DSMB)

The information provided in this section of the protocol is a general description of the DSMB responsibilities and processes. A DSMB charter for the HOPE Consortium includes additional detail. The NIDDK DSMB charter is provided in **Section 14**.

A DSMB has been established by the NIDDK to advise the Institute. The DSMB is comprised of individuals with expertise in clinical trials design and methodology, biostatistics, clinical nephrology, pain, opioids, behavioral interventions, and other relevant medical specialties. The DSMB members are appointed by the NIDDK and are not otherwise affiliated with the study. DSMB members will be free of conflicts of interest that could be affected by the outcomes of the study. During the study, DSMB members who develop real or perceived conflicts of interest that impact objectivity will disclose them to NIDDK project officers, who will arrange for replacement of the member, if indicated.

The DSMB will review the protocol before initiation of the study. After initial approval, the primary responsibilities of the DSMB during the course of the study will be to:

- Review safety data and provide input to protect the safety of the study participants;
- Provide input on major changes to the research protocol and plans for data and safety monitoring;
- Provide input on the progress of the study, including periodic assessments of data quality and timeliness, participant recruitment, accrual and retention, participant risk versus benefit, performance of the study sites, and other factors that may affect study outcomes;
- Consider factors external to the study when relevant information becomes available, such as scientific or therapeutic developments that may have an impact on the need for continuation of the study, safety of the participants or the ethics of the study;

- Provide input on modification of the study protocol or possible early termination of the study because of attainment of study objectives, safety concerns, or inadequate performance (such as enrollment and retention problems).

11.6. Quality Assurance and Quality Control

The following processes or products will be used to maximize data quality, trial fidelity, and standardization across study sites.

Manual of Procedures (MOP) – The MOP will provide detailed instructions for implementing the trial including, but not limited to, the performance of screening, baseline, enrollment, treatment allocation, intervention administration, data collection, and follow-up procedures. The MOP will provide instructions for case report form completion, use of the electronic data management system, and ordering buprenorphine from the central investigational pharmacy. The MOP will be available electronically to study personnel and will be updated online as necessary throughout the trial.

Training and certification procedures – The SDRC will conduct required training sessions for investigators and research personnel before the study starts to train and certify research team members in the performance of study procedures. Separate training sessions led by consortium investigators with expertise in PCST and buprenorphine will be held for PCST coaches and buprenorphine prescribers, respectively.

Quality Control (QC) Committee – The QC Committee will include investigators, research coordinators, and SDRC personnel. The Committee will meet regularly throughout the duration of the trial to review study-wide and site-specific reports generated by the SDRC using data from the data management system. The reports will include information about visit completion, study procedure completion, data completeness, timeliness of data entry, query/discrepancy resolution, PCST and buprenorphine intervention fidelity metrics (see Section 6.4), and completeness of the centralized PRO ascertainment. The QC Committee will report to the Steering Committee and will work with research teams to improve processes as needed.

Recruitment Committee – The Recruitment Committee will include investigators, research coordinators, SDRC personnel, and patient advisors. The committee will produce recruitment materials and dialysis facility informational materials that will be used by the Clinical Centers. The committee will monitor participant enrollment and retention throughout the duration of the trial using reports generated by the SDRC, and will work with research teams to identify approaches to improve performance as needed. Recruitment reports will be distributed study wide and reviewed on Steering Committee teleconferences. Clinical Centers will develop approaches to expanding to new enrollment sites if targets are not being met. The Steering Committee can consider modifications to the protocol to increase the duration of the enrollment period or facilitate enrollment in other ways if necessary. Such changes will need to be approved by the NIDDK and the DSMB, as well as the IRB, before being adopted.

Standardized Instructions – Standardized, written instructions for use of the telehealth and IVR platforms will be provided to participants for the PCST intervention. Standardized safety information and instructions will be provided to participants receiving the buprenorphine intervention.

Site visits – Site visits with individual Clinical Center teams will be conducted either virtually or in-person while the trial is underway.

Internal data quality control procedures – A data validation plan, rule set specifications, and programming logic to implement data validation rules will be implemented in the data management system.

Reports – Reports created regularly by the SDRC will include 1) Recruitment, Retention, and Follow-Up, 2) Safety, and 3) Quality Control. These reports will be used by the relevant internal committees, as well as by the DSMB.

11.7. Data Handling and Record Keeping

The SDRC at the University of Pennsylvania is responsible for the overall management and monitoring of the trial data. Data collected at the Clinical Centers will be entered via a web-based Oracle relational data management system using electronic case report forms that incorporate range and logical edit checks, both within and cross forms. Data entry will be followed daily with manual and programmed checks and edits for errors and omissions.

Access to the data management system will require individual user accounts with role-appropriate privileges. All data will be maintained in a secure electronic database located in the SDRC's professionally managed data center employing FISMA compliant controls. The data management system will import data from electronic sources such as device outputs or files containing analysis results using processes that ensure secure transmission.

The data management system is designed to prevent unauthorized access to trial data and to prevent data loss due to equipment failure or catastrophic events. The procedures to do so encompass user account management, user privilege assignment, data loss prevention (database backup), and data management system change management. User access will be controlled by assignment of confidential usernames and passwords.

11.7.1. Study Records Retention

Clinical Center investigators will retain study documents, including participant files and Regulatory Binders, for at least 5 years after the close of the study, or longer depending on site institutional requirements. The specific processes for destroying records will be in accordance with local institutional requirements.

11.8. Protocol Deviations

A protocol deviation is any change or alteration to the IRB-approved protocol without prospective IRB approval. A protocol exception is a one-time, intentional change or alteration to the IRB-approved protocol that is approved by the IRB prior to implementation.

Major Deviation: Any change/alteration that has or has the potential to: 1) adversely affect the rights, welfare or safety of the subjects, 2) adversely affect the integrity of the research data, or 3) affect the subject's willingness to participate.

Examples of Major Deviations

- Failure to obtain informed consent (i.e., no documentation of informed consent or informed consent was obtained after initiation of study procedures)
- Informed consent obtained by someone not approved to obtain consent for the protocol
- Use of invalid consent form (i.e., consent form without IRB approval stamp, or outdated/expired consent form)
- Enrollment of a participant who is ineligible for the study
- Performing a research procedure not in the approved protocol
- Failure to report serious adverse event to sponsor/SDRC
- Study medication dispensing or dosing error
- Failure to follow the approved study protocol that affects participant safety or data integrity (e.g., failure to properly schedule study visits or failure to perform laboratory tests)
- Continuing research activities after IRB approval has expired
- Use of recruitment procedures that have not been approved by the IRB
- Participant giving study medication to a third-party
- Enrolling significantly more participants than approved in the IRB protocol

Minor Deviation: Any change/alteration that has not or does not have the potential to:
1) adversely affect the rights, welfare or safety of the subjects, 2) adversely affect the integrity of the research data, or 3) affect the subject's willingness to participate.

Examples of Minor Deviations

- Delay in a study visit beyond the protocol-defined window
- Failure to obtain a blood sample for laboratory measurements that are not related to safety monitoring

Site investigators may not implement any major deviation from the protocol without prior review and agreement by the SDRC, and in accordance with IRB and local regulations, except when necessary to eliminate an immediate hazard to study subjects. When a deviation from the protocol is deemed necessary for an individual participant, the investigator must review the issue with the SDRC leadership. Such contact must be made as soon as possible to permit a review by the SDRC, and, if needed, the NIDDK, to determine the impact of the deviation on the participant and/or the study. Any significant protocol deviations affecting participant eligibility and/or safety must be reviewed and/or approved by the IRB and regulatory authorities, as applicable, prior to implementation. Minor protocol deviations (e.g., participant visit outside the study window) that do not impact participant eligibility or safety will be catalogued by each site and reported to the IRB at each continuing review.

11.9. Publications and Data Sharing

Research results will be made available to the scientific community and public in a timely manner. The primary method by which data will be shared with the scientific community will be through peer-reviewed publications and presentations at scientific and professional society meetings. In addition, data and results will be shared with the DSMB and NIDDK in accordance with scheduled or ad hoc meetings. The study will be registered with clinicaltrials.gov prior to initiation, any changes to the protocol will be submitted to clinicaltrials.gov after they have been approved by the IRB, and trial results will be entered into clinictrials.gov in accordance with the clinicaltrials.gov reporting requirements.

Results of the study carried out under this protocol, or any of the information provided by the HOPE Consortium for the purposes of performing the study, will not be published or passed on to any third party without the consent of the Steering Committee. Any investigator involved with this study is obligated to provide the SDRC with results of all study-related testing and all data derived from the study. Publications arising from the trial or other work of the HOPE Consortium will be subject to approval by the Publications Committee and Steering Committee as detailed in the HOPE Publication Policy. Trial data will be maintained by the SDRC, and data analyses for presentations and publications will be performed by the SDRC.

Data from the study will be submitted to the NIH HEAL Data Repository and/or the NIDDK Central Repository in accordance with the NIH HEAL and the NIDDK data sharing policies, respectively.

11.10. Conflict of Interest Policy

All investigators are required to report all financial conflicts of interest on an annual basis. All conflicts of interest will be reported in manuscripts. If conflicts of interest arise during the conduct of the study, the Steering Committee will review and make determinations regarding any necessary management.

12. PROTOCOL AMENDMENT HISTORY

Version	Date	Description of Change	Brief Rationale
V1.1	12.2.2020	Added language to Section 6.2.6 to clarify that VA recruitment sites will be using VA pharmacies to dispense buprenorphine	VA CIRB required this language be added to the protocol
V1.2	5.7.2021	The language used to discuss pregnancy related issues throughout the protocol has been updated to remove gender-specific terminology.	The new language does not exclude participants who are able to become pregnant but do not identify as women.
		The amount of time that the PRO ascertainment calls are expected to last has been updated to 45 minutes.	This protocol initially included both shorter 25-minute PRO ascertainment calls as well as 45-minute calls. The shorter calls have been removed from the protocol.
		Language in section 5.4 Participant Recruitment and Eligibility Screening has been updated to include the following sentence: “Completion of the pre-screening survey is not required for study participation. Patients may be approached for consent and subsequent screening activities without having completed the pre-screening survey.”	This sentence has been added to make clear that the pre-screening is not a requirement for study participation. Participants can be consented without having completed the pre-screening survey.
		The following sentence has been added to Section 6.1.5.1: “After listening to their weekly coach feedback, participants may leave a message that their coach will access during regular business hours.”	This sentence clarifies how IVR will facilitate communication between PCST coaches and participants after the first 12 weeks of PCST.
		Throughout the protocol the word “interventionist” has been replaced with “coach”.	This is the terminology we use to describe the personnel that deliver the PCST intervention.
		In several sections throughout the protocol, the words “Clinical Center” have been replaced with “enrolling site”.	In the sections where these changes have been made, the term “enrolling site” is a more accurate term since Clinical Centers can include multiple enrolling sites.

Version	Date	Description of Change	Brief Rationale
		In Section 6.2.7.4 Initial Visit with Buprenorphine Prescriber the language has been updated to say:<br>"Following buprenorphine initiation, structured follow-up will occur at multiple time points within the first 3 weeks after buprenorphine initiation. These follow-up visits will occur at 2-3 days, 1 week, 2 weeks, and 3 weeks after initiation, but the timing can be modified based on the judgement of the buprenorphine prescriber."	The language in this section was internally inconsistent.
		Table 6.2 Buprenorphine Dosing has been updated in the following ways:<br>- Column 4 now reads "Typical Dosing Regimens" instead of "Dosing Regimens"<br>- In the "Typical Dosing Regimens" column for both the buccal and sublingual formulations, the option for once daily dosing has been removed<br>Section 6.2.9 Buprenorphine Maintenance During and After the Trial has been modified to remove once daily dosing as a dosing frequency option	Once daily dosing is not a commonly used dosing frequency for either of these formulations.
		Throughout the protocol the Pain Mapping Questionnaire has been added to the list of baseline PRO questionnaires.	The pain mapping questionnaire was unintentionally excluded from the list of baseline PROs.
		Falls have been added to the list of Adverse Events of Interest in Section 9.2 Adverse Events of Interest.	Falls are one of the secondary outcomes as well as an Adverse Event of Interest. Falls were inadvertently left off the list of Adverse Events of Interest in section 9.2.
V1.3	7.16.2021	Language in Section 6.2.8 was revised to clarify the procedures for switching a participant to buprenorphine.	The new language provides additional guidance regarding the appropriate buprenorphine formulation (Belbuca or sublingual buprenorphine/naloxone) to use when switching to buprenorphine from a full agonist opioid.

Version	Date	Description of Change	Brief Rationale
		"Dosages and dosing regimens for Belbuca and sublingual buprenorphine/naloxone are shown in Table 6.2. For participants using the crossover titration approach to transitioning to buprenorphine, Belbuca will be used to initiate the crossover titration. After the transition, Belbuca will be used for all participants who had been using a full agonist opioid at a dose of <30 MME/day. Belbuca will also be used for participants who had been using a full agonist opioid at a dose of 30-35 MME/day, unless the maximal dose of Belbuca (450 mcg twice daily) is insufficient to manage their symptoms. As detailed in the Buprenorphine Manual of Procedures, the dose of buprenorphine will be adjusted (increased or decreased) based on adequacy of pain control, withdrawal symptoms, and tolerability.<br><br>For participants using the traditional approach to transitioning to buprenorphine, either Belbuca or buprenorphine/naloxone can be used as the initial buprenorphine formulation depending on the full agonist opioid MME/day, in accordance with the guidelines in the Buprenorphine Manual of Procedures."	
		Table 6.2, Formulation-Specific Exclusions (buprenorphine/naloxone), has been updated in the following way:<br>Old language:<br>"1. Use of full agonist opioid at dose <60 MME/day"<br><br>New Language:<br>"1. Use of full agonist opioid at dose <30 MME/day"<br><br>The following language has also been added to the footnote:<br>"Note that these exclusions apply to the anticipated buprenorphine formulation that will be used immediately following	This change made to correct an error in the prior wording.

Version	Date	Description of Change	Brief Rationale
		the transition from the full agonist opioid. The exclusion does not apply to dose modifications required after a participant has been transitioned to buprenorphine.”	
		The questionnaire “Internalized Stigma in Chronic Pain Scale” has been added to the list of Week 24 PROs.	This questionnaire is part of an ancillary study.
V1.4	12.2.2021	Removal of randomization for the assignment to the Phase 2 buprenorphine intervention.	The number of participants to initiate buprenorphine is significantly fewer than anticipated. By removing the requirement for randomization to buprenorphine vs no buprenorphine, and instead offering buprenorphine to all participants who meet the buprenorphine eligibility criteria, we will increase the sample and be better able to ascertain the exploratory outcomes of tolerability, acceptability, and efficacy of buprenorphine.
V1.5	3.8.2022	Added language to clarify dental risks and to mitigate those risks.<br>Added language to clarify responsibility for prescribing pain medication if buprenorphine is discontinued.<br>List of adverse events of interest was updated.	The FDA recently issued a report indicating that dental problems are a newly recognized adverse effect of buprenorphine delivered via buccal or sublingual administration. Additional detail about risks and ways to minimize those risks has been provided at the request of the IRB.<br>This language has been changed to allow maximum flexibility when ensuring that a participant has continuity in pain management care, and will help to ensure that participants do not experience a gap in pain medication if they need to switch from buprenorphine back to their full agonist opioid medication.<br>This language has been changed to reflect the events being monitored, and to include dental events in light of the FDA’s notice.
V1.6	2.20.2023	Added language to Section 7.3:	This change allowed recruitment sites to randomize participants beyond our target of 640 if an individual had already consented to participate prior to the 640th randomization.

Version	Date	Description of Change	Brief Rationale
		"Recruitment of new participants will conclude once 640 participants have been randomized. At the time of the 640th randomization, participants who are "in the pipeline" (participants who provided consent and have been screened eligible but have not yet been randomized) will also be randomized and continue in the study."	
V1.7	7.7.2023	Added one-time qualitative interviews for approximately 25 participants who were offered the Phase 2 buprenorphine intervention.	The HOPE Trial DSMB requested this addition to the protocol. The purpose of the interviews is to understand why participants were willing or were not willing to switch from their full agonist opioids to buprenorphine when it was offered to them as part of the HOPE Trial.

13. REFERENCES

1. Kimmel PL, Cohen SD, Weisbord SD. Quality of life in patients with end-stage renal disease treated with hemodialysis: survival is not enough! J Nephrol 2008;21 Suppl 13:S54-8.
2. Davison SN. Pain in hemodialysis patients: prevalence, cause, severity, and management. Am J Kidney Dis 2003;42:1239-47.
3. Kimmel PL, Fwu C-W, Abbott KC, Eggers AW, Kline PP, Eggers PW. Opioid prescription, morbidity, and mortality in United States dialysis patients. Journal of the American Society of Nephrology 2017;28:3658-70.
4. Krebs EE, Gravely A, Nugent S, et al. Effect of Opioid vs Nonopioid Medications on Pain-Related Function in Patients With Chronic Back Pain or Hip or Knee Osteoarthritis Pain: The SPACE Randomized Clinical Trial. JAMA 2018;319:872-82.
5. Busse JW, Wang L, Kamaleldin M, et al. Opioids for Chronic Noncancer Pain: A Systematic Review and Meta-analysis. JAMA 2018;320:2448-60.
6. Edelman EJ, Gordon KS, Crothers K, et al. Association of Prescribed Opioids With Increased Risk of Community-Acquired Pneumonia Among Patients With and Without HIV. JAMA internal medicine 2019.
7. Chou R, Turner JA, Devine EB, et al. The effectiveness and risks of long-term opioid therapy for chronic pain: a systematic review for a National Institutes of Health Pathways to Prevention Workshop. Annals of internal medicine 2015;162:276-86.
8. Vangala C, Niu J, Montez-Rath ME, et al. Hip Fracture Risk among Hemodialysis-Dependent Patients Prescribed Opioids and Gabapentinoids. J Am Soc Nephrol 2020.
9. Li Y-N, Shapiro B, Kim JC, et al. Association between quality of life and anxiety, depression, physical activity and physical performance in maintenance hemodialysis patients. Chronic diseases and translational medicine 2016;2:110-9.
10. Salas J, Scherrer JF, Schneider FD, et al. New-onset depression following stable, slow, and rapid rate of prescription opioid dose escalation. Pain 2017;158:306-12.
11. Pitcher MH, Von Korff M, Bushnell MC, Porter L. Prevalence and profile of high-impact chronic pain in the United States. The Journal of Pain 2019;20:146-60.
12. Murtagh FE, Addington-Hall J, Higginson IJ. The prevalence of symptoms in end-stage renal disease: a systematic review. Advances in chronic kidney disease 2007;14:82-99.
13. Weisbord SD, Fried LF, Arnold RM, et al. Prevalence, severity, and importance of physical and emotional symptoms in chronic hemodialysis patients. Journal of the American Society of Nephrology 2005;16:2487-94.
14. Kronenberg F, Lingenhel A, Neyer U, et al. Prevalence of dyslipidemic risk factors in hemodialysis and CAPD patients. Kidney international 2003;63:S113-S6.
15. Levy AR, Xing S, Brunelli SM, et al. Symptoms of Secondary Hyperparathyroidism in Patients Receiving Maintenance Hemodialysis: A Prospective Cohort Study. American Journal of Kidney Diseases 2019.
16. Naylor KL, McArthur E, Leslie WD, et al. The three-year incidence of fracture in chronic kidney disease. Kidney international 2014;86:810-8.
17. Kutner NG, Zhang R, Huang Y, Wasse H. Falls among hemodialysis patients: potential opportunities for prevention? Clin Kidney J 2014;7:257-63.
18. Brkovic T, Burilovic E, Puljak L. Prevalence and severity of pain in adult end-stage renal disease patients on chronic intermittent hemodialysis: a systematic review. Patient preference and adherence 2016;10:1131.
19. Martell BA, o'Connor PG, Kerns RD, et al. Systematic review: opioid treatment for chronic back pain: prevalence, efficacy, and association with addiction. Annals of internal medicine 2007;146:116-27.
20. Pham PC, Khaing K, Sievers TM, et al. 2017 update on pain management in patients with chronic kidney disease. Clin Kidney J 2017;10:688-97.

21. Atkinson TJ. Dialysis, opioids, and pain management: Where's the evidence? Letters To the Editor 2014.
22. Bohnert AS, Valenstein M, Bair MJ, et al. Association between opioid prescribing patterns and opioid overdose-related deaths. Jama 2011;305:1315-21.
23. Dowell D, Haegerich TM, Chou R. CDC guideline for prescribing opioids for chronic pain—United States, 2016. Jama 2016;315:1624-45.
24. Murtagh FE, Chai M-O, Donohoe P, Edmonds PM, Higginson IJ. The use of opioid analgesia in end-stage renal disease patients managed without dialysis: recommendations for practice. Journal of pain & palliative care pharmacotherapy 2007;21:5-16.
25. Naylor KL, Kim SJ, McArthur E, Garg AX, McCallum MK, Knoll GA. Mortality in Incident Maintenance Dialysis Patients Versus Incident Solid Organ Cancer Patients: A Population-Based Cohort. American Journal of Kidney Diseases 2019;73:765-76.
26. Ishida JH, McCulloch CE, Steinman MA, Grimes BA, Johansen KL. Gabapentin and pregabalin use and association with adverse outcomes among hemodialysis patients. Journal of the American Society of Nephrology 2018;29:1970-8.
27. Morley S, Williams A, Hussain S. Estimating the clinical effectiveness of cognitive behavioural therapy in the clinic: evaluation of a CBT informed pain management programme. Pain 2008;137:670-80.
28. Keefe FJ. Cognitive Behavioral Therapy for Managing Pain. The Clincial Psychologist 1996;49:4-5.
29. Qaseem A, Wilt TJ, McLean RM, Forciea MA, Clinical Guidelines Committee of the American College of P. Noninvasive Treatments for Acute, Subacute, and Chronic Low Back Pain: A Clinical Practice Guideline From the American College of Physicians. Ann Intern Med 2017;166:514-30.
30. Heapy AA, Higgins DM, Goulet JL, et al. Interactive Voice Response-Based Self-management for Chronic Back Pain: The COPES Noninferiority Randomized Trial. JAMA Intern Med 2017;177:765-73.
31. Naylor MR, Naud S, Keefe FJ, Helzer JE. Therapeutic Interactive Voice Response (TIVR) to reduce analgesic medication use for chronic pain management. J Pain 2010;11:1410-9.
32. Lacson E, Jr., Li NC, Guerra-Dean S, Lazarus M, Hakim R, Finkelstein FO. Depressive symptoms associate with high mortality risk and dialysis withdrawal in incident hemodialysis patients. Nephrol Dial Transplant 2012;27:2921-8.
33. Schouten RW, Harmse VJ, Dekker FW, van Ballegooijen W, Siegert CEH, Honig A. Dimensions of Depressive Symptoms and Their Association With Mortality, Hospitalization, and Quality of Life in Dialysis Patients: A Cohort Study. Psychosom Med 2019;81:649-58.
34. Al Salmi I, Larkina M, Wang M, et al. Missed Hemodialysis Treatments: International Variation, Predictors, and Outcomes in the Dialysis Outcomes and Practice Patterns Study (DOPPS). Am J Kidney Dis 2018;72:634-43.
35. Johansen KL, Chertow GM, Ng AV, et al. Physical activity levels in patients on hemodialysis and healthy sedentary controls. Kidney Int 2000;57:2564-70.
36. Unruh ML, Buysse DJ, Dew MA, et al. Sleep quality and its correlates in the first year of dialysis. Clin J Am Soc Nephrol 2006;1:802-10.
37. Abdel-Kader K, Unruh ML, Weisbord SD. Symptom burden, depression, and quality of life in chronic and end-stage kidney disease. Clin J Am Soc Nephrol 2009;4:1057-64.
38. Cukor D, Ver Halen N, Asher DR, et al. Psychosocial intervention improves depression, quality of life, and fluid adherence in hemodialysis. J Am Soc Nephrol 2014;25:196-206.
39. Malinoff HL, Barkin RL, Wilson G. Sublingual buprenorphine is effective in the treatment of chronic pain syndrome. Am J Ther 2005;12:379-84.
40. Sorge J, Sittl R. Transdermal buprenorphine in the treatment of chronic pain: results of a phase III, multicenter, randomized, double-blind, placebo-controlled study. Clin Ther 2004;26:1808-20.

41. Daitch D, Daitch J, Novinson D, Frey M, Mitnick C, Pergolizzi J, Jr. Conversion from high-dose full-opioid agonists to sublingual buprenorphine reduces pain scores and improves quality of life for chronic pain patients. Pain Med 2014;15:2087-94.
42. Daitch J, Frey ME, Silver D, Mitnick C, Daitch D, Pergolizzi J, Jr. Conversion of chronic pain patients from full-opioid agonists to sublingual buprenorphine. Pain Physician 2012;15:ES59-66.
43. Oldfield BJ, Edens EL, Agnoli A, et al. Multimodal treatment options, including rotating to buprenorphine, within a multidisciplinary pain clinic for patients on risky opioid regimens: A quality improvement study. Pain Medicine 2018;19:S38-S45.
44. Elkader A, Sproule B. Buprenorphine. Clinical pharmacokinetics 2005;44:661-80.
45. Filitz J, Griessinger N, Sittl R, Likar R, Schuttler J, Koppert W. Effects of intermittent hemodialysis on buprenorphine and norbuprenorphine plasma concentrations in chronic pain patients treated with transdermal buprenorphine. Eur J Pain 2006;10:743-8.
46. Mehrotra R, Cukor D, Unruh M, et al. Comparative Efficacy of Therapies for Treatment of Depression for Patients Undergoing Maintenance Hemodialysis: A Randomized Clinical Trial. Ann Intern Med 2019;170:369-79.
47. Cleeland CS, Ryan KM. Pain assessment: global use of the Brief Pain Inventory. Ann Acad Med Singapore 1994;23:129-38.
48. Keller S, Bann CM, Dodd SL, Schein J, Mendoza TR, Cleeland CS. Validity of the brief pain inventory for use in documenting the outcomes of patients with noncancer pain. Clin J Pain 2004;20:309-18.
49. Dworkin RH, Turk DC, Farrar JT, et al. Core outcome measures for chronic pain clinical trials: IMMPACT recommendations. Pain 2005;113:9-19.
50. Cleeland CS, Nakamura Y, Mendoza TR, Edwards KR, Douglas J, Serlin RC. Dimensions of the impact of cancer pain in a four country sample: new information from multidimensional scaling. Pain 1996;67:267-73.
51. Mendoza T, Mayne T, Rublee D, Cleeland C. Reliability and validity of a modified Brief Pain Inventory short form in patients with osteoarthritis. Eur J Pain 2006;10:353-61.
52. Osborne TL, Raichle KA, Jensen MP, Ehde DM, Kraft G. The reliability and validity of pain interference measures in persons with multiple sclerosis. J Pain Symptom Manage 2006;32:217-29.
53. Raichle KA, Osborne TL, Jensen MP, Cardenas D. The reliability and validity of pain interference measures in persons with spinal cord injury. J Pain 2006;7:179-86.
54. Chen CX, Kroenke K, Stump T, et al. Comparative Responsiveness of the PROMIS Pain Interference Short Forms With Legacy Pain Measures: Results From Three Randomized Clinical Trials. J Pain 2019;20:664-75.
55. Kean J, Monahan PO, Kroenke K, et al. Comparative Responsiveness of the PROMIS Pain Interference Short Forms, Brief Pain Inventory, PEG, and SF-36 Bodily Pain Subscale. Med Care 2016;54:414-21.
56. Kroenke K, Theobald D, Wu J, Tu W, Krebs EE. Comparative responsiveness of pain measures in cancer patients. J Pain 2012;13:764-72.
57. Dworkin RH, Turk DC, Wyrwich KW, et al. Interpreting the clinical importance of treatment outcomes in chronic pain clinical trials: IMMPACT recommendations. J Pain 2008;9:105-21.
58. Krebs EE, Lorenz KA, Bair MJ, et al. Development and initial validation of the PEG, a three-item scale assessing pain intensity and interference. J Gen Intern Med 2009;24:733-8.
59. McNeely J, Wu LT, Subramaniam G, et al. Performance of the Tobacco, Alcohol, Prescription Medication, and Other Substance Use (TAPS) Tool for Substance Use Screening in Primary Care Patients. Ann Intern Med 2016;165:690-9.
60. Becker WC, Frank JW, Edens EL. Switching From High-Dose, Long-Term Opioids to Buprenorphine: A Case Series. Ann Intern Med 2020.
61. Zwarenstein M, Treweek S, Gagnier JJ, et al. Improving the reporting of pragmatic trials: an extension of the CONSORT statement. BMJ 2008;337:a2390.

62. Laird NM, Ware JH. Random-effects models for longitudinal data. Biometrics 1982;38:963-74.
63. Carpenter JR, Roger JH, Kenward MG. Analysis of longitudinal trials with protocol deviation: a framework for relevant, accessible assumptions, and inference via multiple imputation. J Biopharm Stat 2013;23:1352-71.
64. Hwang IK, Shih WJ, De Cani JS. Group sequential designs using a family of type I error probability spending functions. Stat Med 1990;9:1439-45.
65. O'Brien PC, Fleming TR. A multiple testing procedure for clinical trials. Biometrics 1979;35:549-56.
66. gsDesign: Group Sequential Design. R package version 3.0-1. 2016. at https://CRAN.R-project.org/package=gsDesign.**)**
67. Sullivan MJL BS, Pivik J. The Pain Catastrophizing Scale: Development and validation. Psychological Assessment 1995;7:524-32.
68. Sobell LC, Brown J, Leo GI, Sobell MB. The reliability of the Alcohol Timeline Followback when administered by telephone and by computer. Drug Alcohol Depend 1996;42:49-54.
69. Cleeland C. The Brief Pain Inventory: User Guide. Houston, TX: MD Anderson Cancer Center; 2009. 46. .
70. MacFarlane GJ, Croft PR, Schollum J, Silman AJ. Widespread pain: is an improved classification possible? J Rheumatol 1996;23:1628-32.
71. Cohen SR, Sawatzky R, Russell LB, Shahidi J, Heyland DK, Gadermann AM. Measuring the quality of life of people at the end of life: The McGill Quality of Life Questionnaire-Revised. Palliat Med 2017;31:120-9.
72. Spitzer RL, Kroenke K, Williams JB. Validation and utility of a self-report version of PRIME-MD: the PHQ primary care study. Primary Care Evaluation of Mental Disorders. Patient Health Questionnaire. JAMA 1999;282:1737-44.
73. Spitzer RL, Kroenke K, Williams JB, Lowe B. A brief measure for assessing generalized anxiety disorder: the GAD-7. Arch Intern Med 2006;166:1092-7.
74. Harland N, Georgieff, K,. Development of the Coping Strategies Questionnaire 24, a Clinically Utilitarian Version of the Coping Strategies Questionnaire. Rehabilitation Psychology 2003;48:4.
75. Gruber-Baldini AL, Velozo C, Romero S, Shulman LM. Validation of the PROMIS((R)) measures of self-efficacy for managing chronic conditions. Qual Life Res 2017;26:1915-24.
76. Cella D, Riley W, Stone A, et al. The Patient-Reported Outcomes Measurement Information System (PROMIS) developed and tested its first wave of adult self-reported health outcome item banks: 2005-2008. J Clin Epidemiol 2010;63:1179-94.
77. Buysse DJ, Yu L, Moul DE, et al. Development and validation of patient-reported outcome measures for sleep disturbance and sleep-related impairments. Sleep 2010;33:781-92.
78. Schneider S, Choi SW, Junghaenel DU, Schwartz JE, Stone AA. Psychometric characteristics of daily diaries for the Patient-Reported Outcomes Measurement Information System (PROMIS(R)): a preliminary investigation. Qual Life Res 2013;22:1859-69.
79. Rose M, Bjorner JB, Gandek B, Bruce B, Fries JF, Ware JE, Jr. The PROMIS Physical Function item bank was calibrated to a standardized metric and shown to improve measurement efficiency. J Clin Epidemiol 2014;67:516-26.
80. Zimet GD DN, Zimet SG, Farley GK. The Multidimensional Scale of Perceived Social Support. Journal of Personality Assessment 1988;52:30-41.
81. Weisbord SD, Fried LF, Arnold RM, et al. Development of a symptom assessment instrument for chronic hemodialysis patients: the Dialysis Symptom Index. J Pain Symptom Manage 2004;27:226-40.
82. Hahn EA, Devellis RF, Bode RK, et al. Measuring social health in the patient-reported outcomes measurement information system (PROMIS): item bank development and testing. Qual Life Res 2010;19:1035-44.
83. Williams DR, Yan Y, Jackson JS, Anderson NB. Racial Differences in Physical and Mental Health: Socio-economic Status, Stress and Discrimination. J Health Psychol 1997;2:335-51.

84. (ed) GW. ECDEU Assessment Manual for Psychopharmacology. In: US Department of Health E, and Welfare Public Health Service Alcohol, Drug Abuse, and Mental Health Administration, ed. Rockville, MD1976.
85. Scott W, Yu L, Patel S, McCracken LM. Measuring Stigma in Chronic Pain: Preliminary Investigation of Instrument Psychometrics, Correlates, and Magnitude of Change in a Prospective Cohort Attending Interdisciplinary Treatment. J Pain 2019;20:1164-75.

14. APPENDIX

14.1. Schedule of Procedures

		Pre-Screening[1]	Screening Visit[1]	Phase 1 (PCST or Usual Care)							Phase 2 Buprenorphine		
**Procedure**	**Point of Contact or Collection Method**			**BSL[1] WK 0**	**Wk 4**	**Wk 8**	**Wk 12**	**Wk 16**	**Wk 20**	**Wk 24**	**Wk 28**	**Wk 32**	**Wk 36**
**SCREENING**													
Preliminary eligibility assessment	Pre-Screening Survey	X											
Pain screening tool	RC	X											
Pain chronicity	RC	X											
Informed consent	RC		X							X[2]			
Demographics	RC		X										
Medical history	RC		X										
Dialysis history	RC		X										
Opioid history	RC		X										
Cognitive impairment	RC		X										
Review eligibility	RC		X	X									
Randomization (Phase 1)	RC			X									
Contact Opioid Prescriber	RC			X									
Pregnancy Screen	RC/dialysis unit staff									X[3]			
**PRO ASCERTAINMENT**													
PROs	CATI			X			X[4]			X[4]			X
**PAIN COPING SKILLS (PCST)**													
Telehealth Skill Sessions	PCST Coach			Weekly									
IVR Booster Sessions	Pre-recorded telephone sessions							Daily					
**BUPRENORPHINE**													
Appropriateness/Eligibility for Buprenorphine	Study investigator /designee									X			
Buprenorphine consent/ administration/monitoring	Study investigator /designee									X			
**RESEARCH TEAM CONTACTS**													
Clinical outcome ascertainment	RC			X	X	X	X	X	X	X	X	X	X
Adverse event ascertainment	RC			X	X	X	X	X	X	X	X	X	X
Medication review	RC			X	X	X	X	X	X	X	X	X	X
CATI call scheduling	RC			X	X	X	X	X	X	X	X	X	X
**BIOLOGICAL SAMPLES**													
Blood collection	RC/dialysis unit staff									X[3]			
Saliva collection	RC									X[5]			

[1] Pre-Screening, Screening and Baseline activities may be conducted over the course of several days or may be performed on a single day.
[2] Participants eligible for the buprenorphine intervention will provide written consent before initiating treatment with buprenorphine.
[3] Blood will be collected from participants of childbearing potential who meet the full agonist opioid dose criterion for buprenorphine eligibility criteria before being offered buprenorphine.
[4] At Weeks 12 and 24 the full set of PROs except for the Everyday Discrimination Scale will be ascertained.
[5] Saliva collection for substance use screening will be performed for participants before the initial visit with the buprenorphine prescriber. Additional substance use testing during Phase 2 for those who switch to buprenorphine can be performed at the discretion of the buprenorphine prescriber.
Abbreviations: RC, research coordinator; BSL, baseline; CATI, computer-assisted telephone interviewing; PCST, pain coping skills training; BUP, buprenorphine

14.2. PRO Questionnaire Schedule and Activities During Research Team Contacts

Baseline PRO Questionnaires (Week 0)

- Pain interference – Brief Pain Index (BPI) Interference[47]
- Pain intensity – Brief Pain Index (BPI) Severity[47]
- Pain catastrophizing – Pain Catastrophizing Scale (PCS) SF 6[67]
- Opioid use – Timeline Followback[68]
- Pain Mapping Questionnaire[69, 70]
- Quality of life –Single-Item QOL Scale [SIS] from McGill Quality of Life (MQOL) questionnaire[71]
- Depression – Patient Health Questionnaire (PHQ-9)[72]
- Anxiety – Generalized Anxiety Disorder (GAD-7)[73]
- Coping – Coping Strategies Questionnaire 24 (CSQ-24)[74]
- Self-efficacy - PROMIS Self-Efficacy for Managing Chronic Conditions – Managing Symptoms – Short Form 8A[75] + Single item targeting self-efficacy for pain
- Sleep quality – PROMIS Sleep Disturbance SF 6a + Sleep Duration Question[76, 77]
- Fatigue – PROMIS Fatigue SF 6a[76, 78]
- Physical functioning – PROMIS Physical Functioning SF 6b[76, 79]
- Social support – Multidimensional Scale of Perceived Social Support (MSPSS)[80]
- Dialysis symptom index[81]
- Family Intrusion – PROMIS Satisfaction with Social Roles and Activities[76, 82]
- Discrimination – Everyday Discrimination Scale[83]

Follow-up PRO Questionnaires Weeks 12 & 24

- Pain interference – Brief Pain Index (BPI) Interference[47]
- Pain intensity – Brief Pain Index (BPI) Severity[47]
- Pain catastrophizing – Pain Catastrophizing Scale (PCS) SF 6[67]
- Opioid use – Timeline Followback[68]
- Quality of life – Single-Item QOL Scale [SIS] from McGill Quality of Life (MQOL) questionnaire[71]
- Depression – Patient Health Questionnaire (PHQ-9)[72]
- Anxiety – Generalized Anxiety Disorder (GAD-7)[73]
- Coping – Coping Strategies Questionnaire: 1-item
- Self-efficacy - PROMIS Self-Efficacy for Managing Chronic Conditions – Managing Symptoms – Short Form 8A[75] + Single item targeting self-efficacy for pain
- Sleep quality – PROMIS Sleep Disturbance SF 6a + Sleep Duration Question[76, 77]
- Fatigue – PROMIS Fatigue SF 6a[76, 78]
- Physical functioning – PROMIS Physical Functioning SF 6b[76, 79]
- Social support – Multidimensional Scale of Perceived Social Support (MSPSS)[80]
- Dialysis symptom index[81]
- Satisfaction with treatment - Patient Global Impression of Change (PGIC)[84]
- Family Intrusion – PROMIS Satisfaction with Social Roles and Activities[76, 82]
- Internalized Stigma in Chronic Pain Scale (week 24 only)[85]

Follow-up PRO Questionnaires Week 36

- Pain interference – Brief Pain Index (BPI) Interference[47]
- Pain intensity – Brief Pain Index (BPI) Severity[47]
- Pain catastrophizing – Pain Catastrophizing Scale (PCS) SF 6[67]
- Opioid use – Timeline Followback[68]
- Quality of life – Single-Item QOL Scale [SIS] from McGill Quality of Life (MQOL) questionnaire[71]

- Depression – Patient Health Questionnaire (PHQ-9)[72]
- Anxiety – Generalized Anxiety Disorder (GAD-7)[73]
- Coping – Coping Strategies Questionnaire: 1-item
- Self-efficacy - PROMIS Self-Efficacy for Managing Chronic Conditions – Managing Symptoms – Short Form 8A[75] + Single item targeting self-efficacy for pain
- Sleep quality – PROMIS Sleep Disturbance SF 6a + Sleep Duration Question[76, 77]
- Fatigue – PROMIS Fatigue SF 6a[76, 78]
- Physical functioning – PROMIS Physical Functioning SF 6b[76, 79]
- Social support – Multidimensional Scale of Perceived Social Support (MSPSS)[80]
- Dialysis symptom index[81]
- Satisfaction with treatment - Patient Global Impression of Change (PGIC)[84]
- Family Intrusion – PROMIS Satisfaction with Social Roles and Activities[76, 82]
- Discrimination – Everyday Discrimination Scale[83]

Research Team Contacts (Weeks 4, 8, 12, 16, 20, 24, 28, 32, and 36)

- Ascertainment of hospitalizations
- Ascertainment of falls
- Ascertainment of adverse events (SAEs and Adverse Events of Interest)
- Documentation of changes to opioid prescriber(s)
- Documentation of non-opioid analgesics, antidepressants, anxiolytic and sedatives/hypnotics
- Documentation of non-study behavioral or other non-pharmacologic treatments for pain
- Scheduling of Week 12, Week 24.and Week 36 CATI calls (as applicable)

14.3. Data Safety and Monitoring Board (DSMB) Charter

This charter defines the roles and responsibilities of the Data and Safety Monitoring Board (DSMB) for *The Hemodialysis Pain Reduction Effort (HOPE)* study which is funded by the National Institute of Diabetes and Digestive and Kidney Diseases (NIDDK).

The DSMB will serve in a consultative capacity to the NIDDK in accordance with the guidelines set forth in this charter. Typically, DSMB members review and agree to the charter at the initial meeting. If changes to the charter are necessary, the DSMB reviews and affirms their agreement with the changes. Their concurrence will be noted in the DSMB meeting summary.

DSMB Responsibilities
Generally, the first responsibility of the DSMB will be to approve the final protocol of the clinical study named above, or the study/studies being undertaken by the research network named above so that the study/studies can begin enrolling patients. After initial approval, and at periodic intervals during the course of the study, the DSMB responsibilities are to:

- Provide input to assist NIDDK in protecting the safety of the study participants;
- Provide input to the NIDDK on major changes to the research protocol, informed consent documents and plans for data and safety monitoring;
- Provide input to the NIDDK on the progress of the study, including periodic assessments of data quality and timeliness, participant recruitment, accrual and retention, participant risk versus benefit, performance of the study sites, and other factors that may affect study outcomes;
- Review areas of concern regarding the performance of individual sites and provide comment to the NIDDK on actions to be considered regarding sites that perform unsatisfactorily;
- Consider factors external to the study when relevant information becomes available, such as scientific or therapeutic developments that may have an impact on the safety of the participants or the ethics of the study;
- Provide input to the NIDDK on modification of the study protocol or possible early termination of the study because of attainment of study objectives, safety concerns, low likelihood of showing a benefit of the intervention, or inadequate performance (such as enrollment and retention problems);
- If appropriate, review the interim analysis of efficacy in accordance with stopping rules which are clearly defined in the protocol and have the approval of the NIDDK with concurrence of the DSMB;
- Provide input to the NIDDK on the desirability of proceeding to the full-scale study at the completion of a feasibility phase, if appropriate;
- Provide input to the NIDDK on the potential impact of ancillary studies on the integrity of the parent study; and
- Monitor clinical ancillary studies unless an independent DSMB is established or NIDDK determines that a DSMB is not required.

Membership

The members have been appointed by the NIDDK in consultation with the study investigators. Members of the DSMB shall have no financial, scientific, or other conflict of interest with the study. Collaborators or associates of the investigators in this study are not eligible to serve on the DSMB. Written documentation attesting to absence of conflict of interest is required at least annually, and each time there is a change in site investigators and/or institutions involved in the study.

The NIDDK will select a member of the DSMB to serve as the DSMB chairperson. S/He is responsible for overseeing the meetings and developing the agenda in consultation with the NIDDK. The NIDDK will provide the DSMB Executive Secretary. As appropriate, NIDDK personnel may serve as ex-officio (non-voting) members of the DSMB.

DSMB Meetings

The DSMB will typically meet twice a year, or as deemed necessary by the NIDDK Program Official with input from the DSMB. An NIDDK representative will be present at every meeting of the DSMB. A quorum of more than half of the DSMB members is required in order to convene a meeting of the DSMB.

Meetings shall be closed to the public because discussions may address confidential patient data. Meetings are attended, when appropriate, by the principal investigator and members of his/her staff as well as representatives of the Data Coordinating Center, including the study statistician. Meetings may be convened as conference calls or webinars, as well as in person. In special circumstances, the meetings may also be conducted by email. An emergency meeting of the DSMB may be called at any time by the DSMB chairperson or by the NIDDK Program Official should questions of patient safety arise. The DSMB chairperson must contact the NIDDK Program Official prior to convening any meeting. Meetings may not be held without an NIDDK representative present.

Meeting Format

An appropriate format for DSMB meetings consists of an open, closed (if the DSMB is monitoring a study in which the investigators are masked in any way), and executive session. This format may be modified as needed.

<u>Open Session:</u> Members of the DSMB, the NIDDK, the principal investigator and members of the steering committee, including the study biostatistician may attend the open session. Issues discussed will include the conduct and progress of the study, including patient recruitment, data quality, general adherence and toxicity issues, compliance with protocol, and any other logistical matters that may affect either the conduct or outcome of the study. Proposed protocol amendments will also be presented in this session. Patient-specific data and treatment group data may not be presented in the open session.

<u>Closed Session:</u> The closed session will be attended only by DSMB members, unmasked members of the NIDDK, and the unmasked study biostatistician. The discussion at the closed session is completely confidential. All materials from the closed session will be destroyed at the end of the meeting.

Baseline characteristics, adherence and dropouts, and adverse event data are reviewed by masked intervention groups, and potentially in specified subgroups as appropriate to the trial. Primary and secondary outcomes may also be reviewed at DSMB request, on particular occasions, if information on these outcomes is deemed relevant to fulfilling the DSMB's stated responsibilities. The DSMB may request unmasking of the data for either safety or efficacy concerns. Procedures to accomplish unmasking of either individual or treatment group data are to be specified in the Data and Safety Monitoring Plan.1

Executive Session: The executive session will be attended by DSMB members and the NIDDK Program Official and the DSMB Executive Secretary. During the executive session, the DSMB will discuss the information presented during the closed and open sessions and provide input on the continuation or termination of the study, protocol modification or other changes to the conduct of the study. The DSMB can be unmasked at any time if trends develop either for benefit or harm to the participants.
The DSMB will make a recommendation for either continuation or termination of the study. Termination may be suggested by the DSMB at any time. Reasons for early termination include:

- Serious adverse effects in entire intervention group or in a dominating subgroup;
- Greater than expected beneficial effects;
- A statistically significant difference by the end of the study is improbable;
- Logistical or data quality problems so severe that correction is not feasible.

Sound rationale for either decision (continuation or termination of the study) should be presented to the NIDDK; this information will not be shared with the investigators until/unless the study is terminated. The NIDDK will make the final decision regarding termination and is responsible for notifying the PI of their (NIDDK's) decision to terminate the study.

Reports to the DSMB
Reports will be prepared by the Data Coordinating Center on a quarterly or semi-annual basis as decided by the NIDDK Program Official and the DSMB. The reports will be distributed to the DSMB, the NIDDK Program Official and the DSMB Executive Secretary at least 10 days prior to a scheduled meeting. These reports shall be provided in sealed envelopes within an express mailing package, by secure email, or by access to a secure website, as the DSMB prefers. The contents of the report are determined by the NIDDK with recommendations from the DSMB. Over time, additions and other modifications to these reports may be directed by the NIDDK and the DSMB on a one-time or continuing basis.

Data reports for randomized clinical studies or any study in which the investigators are masked in generally consist of two parts: an Open Report and a Closed Report.

Open Session Report: This portion of the report provides information on study aspects such as accrual, baseline characteristics, and other general information on study status. This report is generally shared with all investigators involved with the clinical study. The reports contained in this section generally include:

- Comparison of Target Enrollment to Actual Enrollment by Month;
- Comparison of Target Enrollment to Actual Enrollment by Site;
- Overall Subject Status by Site, including: Subjects Screened, Enrolled, Active, Completed and Terminated;
- Demographic and Key Baseline Characteristics by Group;
- Treatment Duration for Subjects who Discontinue Therapy;
- Adverse Events/Serious Adverse Events by Site and Subject.

Closed Session Report: This report may contain data on study outcomes, including safety data. Data will be presented by masked treatment groups; however, the DSMB may request that the treatment groups be unmasked to ensure that there are no untoward treatment effects. The Closed Session Report is considered confidential and should be destroyed at the conclusion of the meeting. Data files to be used for interim analyses should have undergone established editing procedures to the extent possible. This report should not be viewed by any members of the clinical study except the designated unmasked study statistician.

Documentation of DSMB Meetings

Meeting Summary: A formal summary containing the DSMB's input on the conduct of the study and their recommendation regarding continuation of the study will be prepared by the DSMB Executive Secretary. Each DSMB summary will include the DSMB's recommendation regarding continuation or termination of the study. The DSMB meeting summary will not include unmasked data, discussion of the unmasked data, or any other confidential data. Once completed, the summary is sent to the DSMB members for their review and concurrence. When the summary is satisfactory to the DSMB members and concurrence with the summary is received, the summary will be sent to the PI. It is the responsibility of the PI to distribute the summary to all co-investigators.

Substantiation of the DSMB Recommendation Regarding Study Continuation: When requested by the NIDDK, the DSMB will prepare a statement explaining the rationale for their recommendation to continue or terminate the study. This statement will be provided directly to the NIDDK Program Official and will not be shared with the investigators or masked NIDDK personnel.

Letter from the NIDDK Program Official to the Investigators: A letter to the investigators from the NIDDK Program Official will accompany the DSMB summary following each DSMB meeting. This letter will contain any guidance from the NIDDK Program Official in reference to the DSMB summary.

It is the responsibility of the study investigators to assure that the letter from the Program Official is submitted to all the Institutional Review Boards (IRBs) associated with the study. If the meeting summary is to be submitted to the IRBs in addition to the Program Official's letter, the letter will so state.

Confidentiality and Objectivity

All materials, discussions, and proceedings of the DSMB are completely confidential. Members and other participants in DSMB meetings are expected to maintain confidentiality. Closed session meeting materials should be destroyed in a secure manner (shredding) following each meeting.

In order to maintain their objectivity, DSMB members are expected not to discuss the study/studies with the investigators except during DSMB meetings. Questions or concerns that arise between DSMB meetings that might lead to discussion between DSMB members and the investigators should be brought to the attention of the NIDDK Program Official.
