# Supplementary material for: Pain Coping Skills Training for Patients Receiving Hemodialysis: The HOPE Consortium Randomized Clinical Trial
Source: JAMA Intern Med. 2024 Dec 30;185(2):197–207. doi: 10.1001/jamainternmed.2024.7140 (PMC11791705; doi:10.1001/jamainternmed.2024.7140)
Supplement: Supplement 2. — Statistical Analysis Plan [file jamainternmed-e247140-s002.pdf]

Statistical Analysis Plan for the HOPE Trial Primary Results Manuscript

Date Approved by Publications Committee: December 18, 2023

This document provides the statistical analysis approach for the report of the primary results of the trial. The full statistical analysis plan for the HOPE Trial is provided in the Protocol V1.7, Section 10; pages 54-66.

General Approach
The following descriptive statistics will be used: number of observations, mean, standard deviation, median, minimum and maximum for continuous variables; frequencies and proportions for categorical variables; number of events and rates for count variables. Results will be reported in accordance with the extended CONSORT guidelines for pragmatic clinical trials using two-sided statistical tests and confidences intervals. The overall level of significance will be set at 0.046 for the primary outcome to account for the pre-specified interim analysis performed when approximately 50% of participants had reached Week 12. For other analyses, the level of significance will be 0.05. Regression analyses will be adjusted for site and the presence or absence of opioid use at baseline. Analyses will be performed using the latest version of R.

Baseline Descriptive Statistics
Randomization adequacy will be assessed by comparing the distributions of baseline demographic and clinical characteristics between the PCST and Usual Care groups. Comparability for continuous variables will be examined graphically and by summary statistics: mean, standard deviation, median, minimum, and maximum for continuous variables, and frequency and proportion for and categorical variables. If statistically and/or clinically meaningful differences are found in baseline characteristics (i.e., baseline characteristics are not balanced between the PCST and Usual Care groups), sensitivity analyses will be conducted with covariate adjustment.

Analyses of the Primary Outcome: BPI Interference
The primary analysis will compare the change from baseline to week 12 in the BPI Interference scores between the PCST and Usual Care groups using a linear mixed effects model and an intent to treat approach in which all available data on all randomized participants are included. The model will include fixed effects of intervention, time, and interactions between intervention and time, accounting for repeated measures and the 16 enrolling sites via random effects. The model will include all available data points from baseline, week 12, week 24, and week 36. The contrast for the change from baseline to week 12 in the BPI Interference scores between the PCST and Usual Care groups will be represented by the coefficient of the interaction between intervention and week 12. Similar contrasts will be reported from the same model: 1) to determine the effect of the full PCST intervention (coach-led component plus IVR component), the contrast for the change from baseline to week 24 in the BPI Interference scores between the PCST and Usual Care groups will be used, and 2) to determine the durability of the effects of PCST, the contrast for the change from baseline to week 36 in the BPI Interference scores between the PCST and Usual Care groups will be used. A responder analysis will be performed in which the proportion of participants with a reduction from baseline in the BPI Interference score that is greater than 1 point (minimal clinically important difference) will be compared between the PCST and usual care groups using a general estimating equation (GEE) model with a binomial distribution and logit link. The model will include the intervention, time, interaction between intervention and time, and opioid use at baseline. Participants will be treated as clusters with an exchangeable correlation structure. The responder analysis will be performed for each of the three time points (week 12, week 24, and week 36).

Analyses of Secondary Outcomes
For continuous secondary outcomes (BPI Severity, Pain Catastrophizing, CSQ-24, Single item QOL, PHQ-9, and GAD-7), the approach described for the BPI Interference will be used. For binary secondary outcomes, such as the composite of

pain interference and opioid use, generalized estimating equations with a binomial distribution and a log link adjusting for baseline measures will be used. For recurrent endpoints such as hospitalizations or falls, generalized estimating equations with a Poisson distribution, an offset of follow-up time, and a log link adjusting for baseline measures will be used to compare event rates. In all aforementioned generalized estimating equations, participants will be treated as clusters with an exchangeable correlation structure. For comparisons between the PCST and Usual Care groups that only use outcomes at week 36, additional adjustment for buprenorphine eligibility and for buprenorphine initiation will be incorporated.

Missing Outcome Data

The multivariate imputation by chained equations method will be used to handle missing data in both outcomes and covariates. A fixed number of imputed datasets (e.g., 100) will be included. Results from regression models in each imputed dataset are combined using Rubin's Rules. All analyses will be adjusted for the randomization stratification factors.

Subgroup Analyses

Potential effect modification will be examined by including interaction terms between interventions and the following baseline characteristics: gender, race, age, and opioid use. For baseline factors with statistically significant interactions, intervention effects will be reported by subgroups.

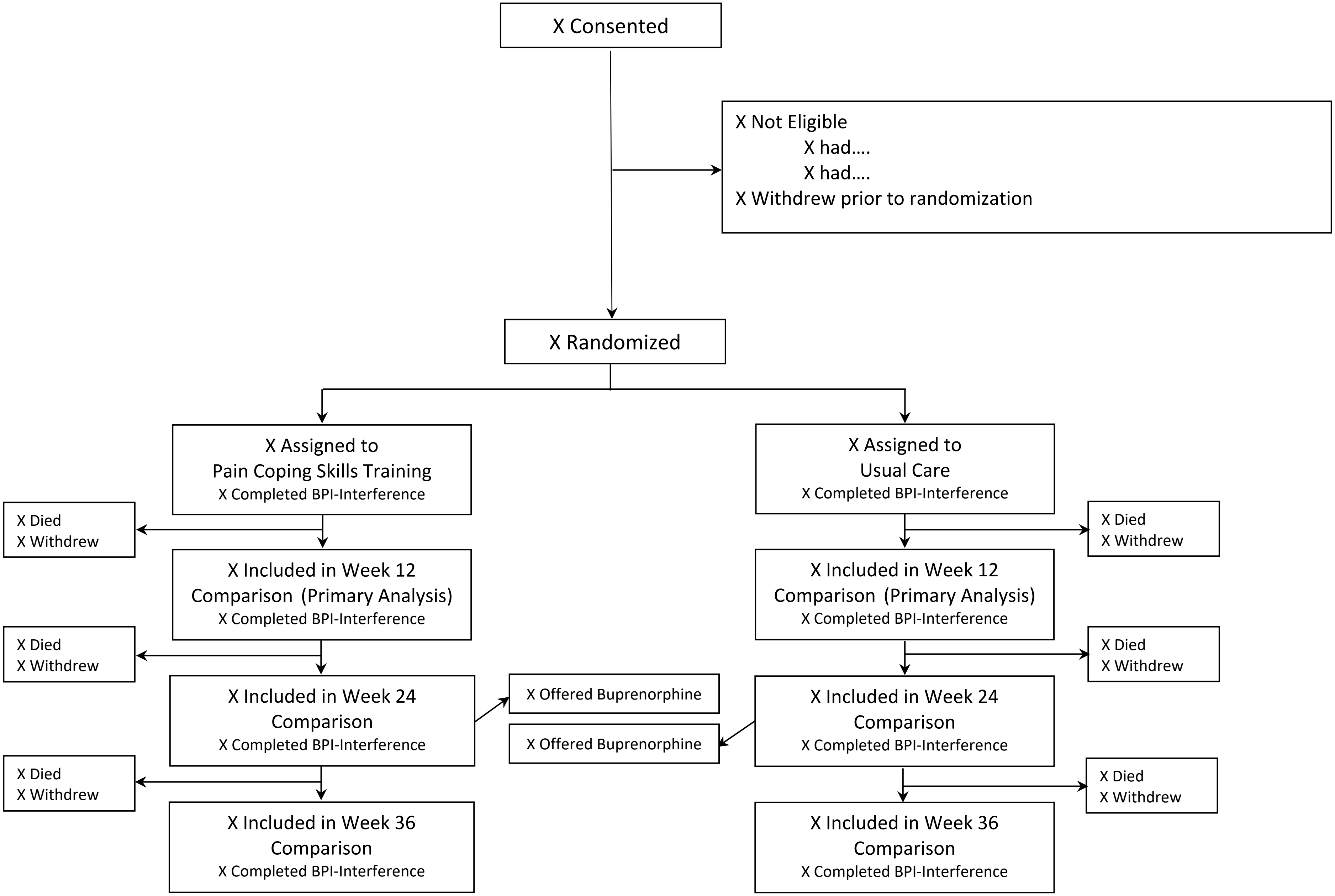

Figure 1. Participant Enrollment and Follow-Up

Table 1. Baseline Characteristics

	Overall	PCST Group	Usual Care Group
**Demographic Characteristics**			
Age, years			
Female			
American Indian or Alaskan Native			
Asian			
Black			
Native Hawaiian or Other Pacific Islander			
White			
Multiple Races			
Race not reported			
Hispanic or Latino			
Ethnicity not reported			
**Clinical Characteristics**			
Post-dialysis BMI, kg/m2			
Pre-dialysis systolic BP, mm Hg			
Pre-dialysis diastolic BP, mm Hg			
Duration of dialysis treatment, years			
**Comorbidities**			
Diabetes mellitus			
Coronary artery disease			
Heart failure			
Atrial fibrillation or flutter			
Ventricular arrhythmia			
Heart valve replacement or repair			
Stroke or transient ischemic attack			
Peripheral vascular disease			
Cancer			
**Substance Use**			
Current tobacco use			
Current alcohol use			
Current cannabis use			
**Medications**			
Opioid use during 3 of the last 6 months			
Opioid use during the last 14 days			
Average MME/day for those with opioid use during the last 14 days			
**Laboratory Values**			
Blood urea nitrogen, mg/dL			
Creatinine, mg/dL			
Albumin, g/dL			
Hemoglobin, g/dL			
Bicarbonate, mEq/L			
Kt/V			

Values are means (SD), n (%), or median (25th- 75th percentile)
Abbreviations: BMI, body mass index; BP, blood pressure; MME, morphine milligram equivalents

Table 2. Change in Brief Pain Inventory - Interference Scale (BPI - Interference)

	Pain Coping Skills Training			Usual Care			Between-Group Difference[1] Change n; Mean (SD)
	Baseline n; Mean (SD)	Follow-Up n; Mean (SD)	Change n; Mean (SD)	Baseline n Mean (SD)	Baseline n; Mean (SD)	Follow-Up n; Mean (SD)	
**Week 12**	n; Mean (SD)	n; Mean (SD)	n; Mean (SD)	**Week 12**	n; Mean (SD)	n; Mean (SD)	n; Mean (SD)
**Week 24**	n; Mean (SD)	n; Mean (SD)	n; Mean (SD)	**Week 24**	n; Mean (SD)	n; Mean (SD)	n; Mean (SD)
**Week 36**	n; Mean (SD)	n; Mean (SD)	n; Mean (SD)	**Week 36**	n; Mean (SD)	n; Mean (SD)	n; Mean (SD)

Score Range for BPI – Interference: 0-10, higher score indicates more pain interference
[1]based on linear mixed effects models

Table 3. Change in Secondary Patient Reported Outcomes

	Pain Coping Skills Training			Usual Care			Between-Group Difference
	**Baseline**<br>n;<br>Mean (SD)	**Follow-Up**<br>n;<br>Mean (SD)	**Change**<br>n;<br>Mean (SD)	**Baseline**<br>n<br>Mean (SD)	**Baseline**<br>n;<br>Mean (SD)	**Follow-Up**<br>n;<br>Mean (SD)	Mean (95% CI)<br>P-Value
**Week 12**							
**BPI Severity**	n;<br>Mean (SD)	n;<br>Mean (SD)	n;<br>Mean (SD)	n;<br>Mean (SD)	n;<br>Mean (SD)	n;<br>Mean (SD)	Mean (95% CI)<br>P-Value
**Pain Catastrophizing**	n;<br>Mean (SD)	n;<br>Mean (SD)	n;<br>Mean (SD)	n;<br>Mean (SD)	n;<br>Mean (SD)	n;<br>Mean (SD)	Mean (95% CI)<br>P-Value
**Single Item QoL**	n;<br>Mean (SD)	n;<br>Mean (SD)	n;<br>Mean (SD)	n;<br>Mean (SD)	n;<br>Mean (SD)	n;<br>Mean (SD)	Mean (95% CI)<br>P-Value
**CSQ-24**	n;<br>Mean (SD)	n;<br>Mean (SD)	n;<br>Mean (SD)	n;<br>Mean (SD)	n;<br>Mean (SD)	n;<br>Mean (SD)	Mean (95% CI)<br>P-Value
**PHQ-9**	n;<br>Mean (SD)	n;<br>Mean (SD)	n;<br>Mean (SD)	n;<br>Mean (SD)	n;<br>Mean (SD)	n;<br>Mean (SD)	Mean (95% CI)<br>P-Value
**GAD-7**	n;<br>Mean (SD)	n;<br>Mean (SD)	n;<br>Mean (SD)	n;<br>Mean (SD)	n;<br>Mean (SD)	n;<br>Mean (SD)	Mean (95% CI)<br>P-Value
**Week 24**							
**BPI - Severity**	n;<br>Mean (SD)	n;<br>Mean (SD)	n;<br>Mean (SD)	n;<br>Mean (SD)	n;<br>Mean (SD)	n;<br>Mean (SD)	Mean (95% CI)<br>P-Value
**Pain Catastrophizing**	n;<br>Mean (SD)	n;<br>Mean (SD)	n;<br>Mean (SD)	n;<br>Mean (SD)	n;<br>Mean (SD)	n;<br>Mean (SD)	Mean (95% CI)<br>P-Value
**CSQ-24**	n;<br>Mean (SD)	n;<br>Mean (SD)	n;<br>Mean (SD)	n;<br>Mean (SD)	n;<br>Mean (SD)	n;<br>Mean (SD)	Mean (95% CI)<br>P-Value
**Single Item QoL**	n;<br>Mean (SD)	n;<br>Mean (SD)	n;<br>Mean (SD)	n;<br>Mean (SD)	n;<br>Mean (SD)	n;<br>Mean (SD)	Mean (95% CI)<br>P-Value
**PHQ-9**	n;<br>Mean (SD)	n;<br>Mean (SD)	n;<br>Mean (SD)	n;<br>Mean (SD)	n;<br>Mean (SD)	n;<br>Mean (SD)	Mean (95% CI)<br>P-Value
**GAD-7**	n;<br>Mean (SD)	n;<br>Mean (SD)	n;<br>Mean (SD)	n;<br>Mean (SD)	n;<br>Mean (SD)	n;<br>Mean (SD)	Mean (95% CI)<br>P-Value

	Pain Coping Skills Training			Usual Care			Between-Group Difference
	**Baseline** n; Mean (SD)	**Follow-Up** n; Mean (SD)	**Change** n; Mean (SD)	**Baseline** n Mean (SD)	**Baseline** n; Mean (SD)	**Follow-Up** n; Mean (SD)	Mean (95% CI) P-Value
**Week 36**							
**BPI- Severity**	n; Mean (SD)	n; Mean (SD)	n; Mean (SD)	n; Mean (SD)	n; Mean (SD)	n; Mean (SD)	Mean (95% CI) P-Value
**Pain Catastrophizing**	n; Mean (SD)	n; Mean (SD)	n; Mean (SD)	n; Mean (SD)	n; Mean (SD)	n; Mean (SD)	Mean (95% CI) P-Value
**CSQ-24**	n; Mean (SD)	n; Mean (SD)	n; Mean (SD)	n; Mean (SD)	n; Mean (SD)	n; Mean (SD)	Mean (95% CI) P-Value
**Single Item QoL**	n; Mean (SD)	n; Mean (SD)	n; Mean (SD)	n; Mean (SD)	n; Mean (SD)	n; Mean (SD)	Mean (95% CI) P-Value
**PHQ-9**	n; Mean (SD)	n; Mean (SD)	n; Mean (SD)	n; Mean (SD)	n; Mean (SD)	n; Mean (SD)	Mean (95% CI) P-Value
**GAD-7**	n; Mean (SD)	n; Mean (SD)	n; Mean (SD)	n; Mean (SD)	n; Mean (SD)	n; Mean (SD)	Mean (95% CI) P-Value

Abbreviations: BPI – Severity, Brief Pain Interference – Severity; CSQ-24, Coping Strategies Questionnaire – 24-item; MME, morphine milligram equivalents; QoL, quality of life; PHQ-9, Patient Health Questionnaire – 9-item; GAD-7, Generalized Anxiety Disorder, 7-item

Score Ranges and Meaning of Higher Score: BPI-Severity: 0-10, higher severity; Pain Catastrophizing: 0-24, more catastrophizing; CSQ-24, 0-42 per component, more coping; Single item QoL, 0-10, better quality of life; PHQ-9, 0-27, more depression; GAD-7, 0-21, more anxiety

Table 4. Opioid Use Outcomes

MME/Day							
	Pain Coping Skills Training			Usual Care			Between-Group Difference
	Baseline n; Mean (SD)	Follow-Up n; Mean (SD)	Change n; Mean (SD)	Baseline n; Mean (SD)	Follow-Up n; Mean (SD)	Change n; Mean (SD)	Mean (95% CI) P-Value
**Week 12**	n; Mean (SD)	n; Mean (SD)	n; Mean (SD)	n; Mean (SD)	n; Mean (SD)	n; Mean (SD)	Mean (95% CI) P-Value
**Week 24**	n; Mean (SD)	n; Mean (SD)	n; Mean (SD)	n; Mean (SD)	n; Mean (SD)	n; Mean (SD)	Mean (95% CI) P-Value
**Week 36**	n; Mean (SD)	n; Mean (SD)	n; Mean (SD)	n; Mean (SD)	n; Mean (SD)	n; Mean (SD)	Mean (95% CI) P-Value

Composite Outcome of Pain Interference and Opioid Use			
	**Pain Coping Skills Training % Success[1]**	**Usual Care % Success[1]**	**OR (95% CI)**
**Week 12**	%	%	
**Week 24**	%	%	
**Week 36**	%	%	

[1]Success is defined as Stable or Improved Pain Interference and Stable or Improved Opioid Use

Table 5. Clinical Outcomes

	PCST		Usual Care		Rate Ratio (95% CI)<br>P-Value
	**Pts w/event Number (%)**	**Number of Events per Pt-Year**	**Pts w/event Number (%)**	**Number of Events per Pt-Year**	
**Death**	Number (%)	Number/Pt-Year	Number (%)	Number/Pt-Year	RR (X – Y)<br>P-Value
**Hospitalization**	Number (%)	Number/Pt-Year	Number (%)	Number/Pt-Year	RR (X – Y)<br>P-Value
**Fall**	Number (%)	Number/Pt-Year	Number (%)	Number/Pt-Year	RR (X – Y)<br>P-Value

Table 6. Adverse Events

<table>
<tr><th colspan="2" rowspan="2"></th><th colspan="2">Pain Coping Skills Training</th><th colspan="2">Usual Care</th><th rowspan="2">P-value for # pts w/event</th><th rowspan="2">P-value for event rate</th></tr>
<tr><th>Pts w/event No. (%)</th><th>Events per pt-year</th><th>Pts w/event No. (%)</th><th>Events per pt-year</th></tr>
<tr><td colspan="2">Any Serious Adverse Event</td><td></td><td></td><td></td><td></td><td></td><td></td></tr>
<tr><td rowspan="5">AEs of Interest</td><td>Suicidality Alert[1]</td><td>Number (%)</td><td>Number/Pt-Year</td><td>Number (%)</td><td>Number/Pt-Year</td><td></td><td></td></tr>
<tr><td>Opioid Withdrawal Symptoms[2]</td><td>Number (%)</td><td>Number/Pt-Year</td><td>Number (%)</td><td>Number/Pt-Year</td><td></td><td></td></tr>
<tr><td>Medication-Related Event[3]</td><td>Number (%)</td><td>Number/Pt-Year</td><td>Number (%)</td><td>Number/Pt-Year</td><td></td><td></td></tr>
<tr><td>Development of Opioid Use Disorder[4]</td><td>Number (%)</td><td>Number/Pt-Year</td><td>Number (%)</td><td>Number/Pt-Year</td><td></td><td></td></tr>
<tr><td>Development of Non-Opioid Substance Use Disorder[4]</td><td>Number (%)</td><td>Number/Pt-Year</td><td>Number (%)</td><td>Number/Pt-Year</td><td></td><td></td></tr>
</table>

[1]Based on response to Question 9 of PHQ-9 questionnaire
[2]Ascertained if reduction in opioid medication dose
[3]Adverse effect from a medication started during the trial to treat pain, depression or anxiety
[4]Based on responses to the TAPS Questionnaire

Changes to the Primary Results Paper Analysis Plan After Initial Approval by the Publications Committee

1. The Coping Strategies 24 Item Questionnaire (CSQ 24) was removed as a secondary outcome because it was completed only at baseline and not at 12, 24, and 36 weeks. The reason for including this questionnaire at baseline was to characterize the extent to which participants had coping strategies at the time of enrollment for use as an exposure variable in secondary analyses exploring factors associated with pain-associated outcomes and response to the intervention. The CSQ-24 Single Item Questionnaire was completed at baseline and Weeks 12, 24, and 36; however, the single item questionnaire measures catastrophizing rather than coping. Because catastrophizing is captured more fully with the Pain Catastrophizing Scale SF-6, the decision was made to not include the CSQ-24 Single Item measure.

2. The table below showing the proportion of participants in each randomized group with a decrease in the BPI Interference Score of >1 point (the minimal clinical important difference) was inadvertently omitted from the original Statistical Analysis Plan for the Primary Results Manuscript. The decision to include this responder analysis was made at a Steering Committee Meeting on March 31, 2023 (approximately 9 months before the end of participant follow-up).

**Proportion with Decrease in BPI Interference Score of >1 Point**			
	**Decrease in BPI Interference Score >1 Point**		**OR (95% CI)**
	**PCST No. (%)**	**Usual Care No. (%)**	
**Week 12**	Number (%)	Number (%)	OR (95% CI)
**Week 24**	Number (%)	Number (%)	OR (95% CI)
**Week 36**	Number (%)	Number (%)	OR (95% CI)

3. P-values for all analyses except the primary endpoint (change in BPI Interference Score at Week 12) were removed from the tables. This change was made since adjustment for multiple comparisons was not performed.

4. The MME/day outcome was included in the table with the other patient-reported secondary outcomes rather than in a separate table of opioid use outcomes.
