# Supplementary material for: Pain Coping Skills Training for Patients Receiving Hemodialysis: The HOPE Consortium Randomized Clinical Trial
Source: JAMA Intern Med. 2024 Dec 30;185(2):197–207. doi: 10.1001/jamainternmed.2024.7140 (PMC11791705; doi:10.1001/jamainternmed.2024.7140)
Supplement: Supplement 3. — eAppendix. HOPE Consortium, Cores, and Data and Safety Monitoring Board Members eTable 1. Eligibility Criteria eTable 2. Training, Certification, Supervision, and Fidelity Monitoring of PCST Coaches eTable 3. Complete List of Outcome Measures eTable 4. Adherence to Coach-Led PCST Sessions Among Participants Randomized to PCST Group eTable 5. Delivery Method and Location for Coach-Led PCST Sessions eTable 6. Sensitivity Analysis: Effect of Pain Coping Skills Training on Patient-Reported Outcomes Using Multiple Imputation to Address Missing Outcomes eTable 7. Sensitivity Analysis: Comparison of Models with Enrolling Site versus Dialysis Facility as Random Effect (enrolling site was used in primary analysis) eTable 8. Sensitivity Analysis: Decrease of >30% in BPI Interference Score eTable 9. Composite Outcome of Pain Interference and Opioid Use eTable 10. Clinical Event Outcomes eTable 11. Adverse Events eFigure. Subgroup Analysis of the Primary Endpoint: Change in BPI Interference between Baseline and Week 12 [file jamainternmed-e247140-s003.pdf]

Supplemental Online Content

Dember LM, Hsu JY, Mehrotra R, et al. Pain coping skills training for patients receiving hemodialysis: the HOPE consortium randomized clinical trial. *JAMA Intern Med*. Published online December 30, 2024. doi:10.1001/jamainternmed.2024.7140

eAppendix. HOPE Consortium, Cores, and Data and Safety Monitoring Board Members

eTable 1. Eligibility Criteria

eTable 2. Training, Certification, Supervision, and Fidelity Monitoring of PCST Coaches

eTable 3. Complete List of Outcome Measures

eTable 4. Adherence to Coach-Led PCST Sessions Among Participants Randomized to PCST Group

eTable 5. Delivery Method and Location for Coach-Led PCST Sessions

eTable 6. Sensitivity Analysis: Effect of Pain Coping Skills Training on Patient-Reported Outcomes Using Multiple Imputation to Address Missing Outcomes

eTable 7. Sensitivity Analysis: Comparison of Models with Enrolling Site versus Dialysis Facility as Random Effect (enrolling site was used in primary analysis)

eTable 8. Sensitivity Analysis: Decrease of >30% in BPI Interference Score

eTable 9. Composite Outcome of Pain Interference and Opioid Use

eTable 10. Clinical Event Outcomes

eTable 11. Adverse Events

eFigure. Subgroup Analysis of the Primary Endpoint: Change in BPI Interference between Baseline and Week 12

This supplemental material has been provided by the authors to give readers additional information about their work.

© 2024 Dember LM et al. *JAMA Intern Med.*

HOPE Consortium, Cores, and Data and Safety Monitoring Board Members

Hennepin Healthcare Clinical Center and Enrolling Site: Kirsten L. Johansen (PI), Gavin Bart, Erin E. Krebs, James B. Wetmore, Maria Pacheco-Hernandez, Ursula Munet, Rudy Qamhiyeh, Mike Wambua; **Massachusetts General Hospital Clinical Center and Enrolling Site**: Sahir Kalim (PI), Sagar U. Nigwekar, Daniel E. Weiner, Kome Ekor, Beza Mengesha, Shananssa Percy; **New York University Langone Health Clinical Center and Enrolling Site**: David M. Charytan (PI), Keith S. Goldfeld, Joshua D. Lee, Nobuyuki Miyawaki, Jennifer S. Scherer, Amanda J. Shallcross, Miri Cazes, Sobaata Chaudry, Paula Dutka, Yasmine Flores, Daniela Fraticelli Ortiz, Candace Grant, Colin Keane, Pragna Krishnamurthy, Ashley Macina, Angela McCarthy, Kathleen Rice, Grace Robinson, Dalila Varela, Javaughn Ways; **University of Illinois Chicago Clinical Center and Enrolling Site**: Michael J. Fischer (PI), Ardith Z. Doorenbos, Christopher Holden, James P. Lash, Mark B. Lockwood, Alana D. Steffen, Cheryl Gilmartin, Amanda Goldstein, Monya Meinel, Kimberly Silva, Guillermo Zamora; **University of Pittsburgh Clinical Center and Enrolling Site**: Manisha Jhamb (PI), Hailey W. Bulls, Megan E. Hamm, Sanjana Kamat, Jane M. Liebschutz, Jennifer L. Steel, Jonathan G. Yabes, Precious Lacey, Donna Olejniczak, Mary Schopp, Melissa Weimer, Vincent Wood; **University of Pennsylvania Enrolling Site**: Nwamaka D. Eneanya (PI), Sarah J. Schrauben (PI), Stephany Almonte-Then, Chigozie Amonu, Nicholas Bishop, Diane Park, Taylor Stallings; **University of Washington Clinical Center and Enrolling Site**: Rajnish Mehrotra R (PI), Nisha Bansal, Elenore P. Bhatraju, Steven D. Weisbord, Lisa Anderson, Sydney Johnson, Kaeleb Laszlo, Lori Linke; **University of New Mexico Enrolling Site**: Mark L. Unruh (PI), Christos P. Argyropoulos, Shane Pankratz, Davin Quinn, George Garcia, Monica Bajana Meza, Monica Cardona, Tammy Seaman Weidner, Greg Trejo; **Rogosin Institute Enrolling Site**: Daniel Cukor (PI), Nathaniel Berman, Nelson Chen, Ines Chicos, Stephanie Donahue, Anna Gong; **Vanderbilt University Medical Center C Clinical Center and Enrolling Site**: Kerri L. Cavanaugh (PI), Carrie E. Brintz, David A. Edwards, T. Alp Ikizler, Puneet Mishra, Thomas Stewart, DeVitra Berry, Don Merrimon, Samuel Opeke, Sarah Pleasant, Hadassah Pegues, Christopher Roach, Sonya Williams; **West Virginia University Enrolling Site**: Bethany Pellegrino (PI), Daniel W. McNeil, Alvin H. Moss, Rebecca J. Schmidt, Cheryl Dalton, Kristy

© 2024 Dember LM et al. *JAMA Intern Med.*

O'Connell, Maryanne Wilkinson; **Yale School of Medicine/VA Connecticut Healthcare System Clinical Center and Enrolling Site**: William C. Becker (PI), Justin M. Belcher, Susan T. Crowley, Denise Esserman, Caroline G. Falker, Alicia A. Heapy, Svetlana Vassilieva, Samara Zuniga; **Durham VA Health Care System Enrolling Site**: Patrick H. Pun (PI), Wissam M. Kourany, James Lefler, Teresa Purdy, Jenika Hammond, Khristian Harris, Sara Hoffman, Jeanette Rutledge; **VA Portland Health Care System**: Benjamin J. Morasco (PI), Christopher K. Blazes, Christopher S. Stauffer, Melissa Adams, Deza'Rae Collins, Richard Torres; **Dallas VA Medical Center Enrolling Site**: Jeffrey Penfield (PI), Monica Barbosa, Levi Beeks, Erik Guajardo, Sindi Sanchez; **VA New York Harbor Health Care Enrolling Site**: Mansi Mehta (PI), Adrian Cosmin, David S. Goldfarb, Sabrina Felson, Brian Sands, Frank Modersitzki; **Scientific Data and Research Center**: **University of Pennsylvania**: Laura M. Dember (PI), Martin D. Cheatle, John T. Farrar, Jesse Y. Hsu, Steve Joffe, Kyle M. Kampman, Francis J. Keefe, J Richard Landis, Ted Barrell, Leah Bernardo, Natalie Kuzla, Jonah Joffe, Joanna Walsh; **Patient Advisors**: Dawn P. Edwards, Robert E. Grindstaff*, Andre Hoover, Stephen Lerner, Roger Mims, Nina Quintana, Steven Sousa, Darlene Villareal, David M. White, Caroline Wilkie, Joel Williams; **Pain Coping Skills Training Coaches**: Veronica Dyer, New York University Langone Health; Eshika Kalam, Rogosin Institute; Blanca Contreras, University of Illinois Chicago; Carlyn Clark, University of Washington; Heather Howell and Kevin Payne, West Haven VA Medical Center; **Pain Coping Skills Training Fidelity Monitors**: Andrew M. Busch and Sarah Cameron, Hennepin Healthcare; **Computer-Assisted Telephone Interviewing (CATI) Centers: University of New Mexico** - Adamaris Arteaga Leon, Elena Ashley, Gabriela Chacon Palma, Katy Chalamidas, Oluoma Edeh, Lindsay Gear, Cameron Guy, Grace Kimura, Alexander Leon Cupe, Henry Luo, Katherine McDaniels, Sofia McLaren, Valeria Mejia, Claire Mullins, Uchechukwu Okereke, Gabriel Rudow, Giselle Rodriguez Sosa, Lily Sullivan, John Torres, Hugo Vilchis; **University of Pittsburgh -** University of Pittsburgh: Scott Beach, Amber Barton, Victoria Casilli, Lynda Connelly, Jane Dirks, Luke Farkas, Ana Geibel, Olivia Kirsch, Paula Kubrick, Patricia Lietz, Brandon Self, Susan Stringfellow, Olivia Wilson; **National Institute of Diabetes and Digestive and Kidney Diseases**: Kevin C. Abbott, Paul L. Kimmel, Jenna M. Norton, Tracy L. Rankin; **Data and Safety Monitoring Board**: Bruce Barton (Chair), Jane Atkinson,

© 2024 Dember LM et al. *JAMA Intern Med.*

Melissa Bensouda, Roger B. Fillingim, Michael Freeman, Andrew Garland, Jennifer J. Gassman, Allen R. Nissenson, Maile Robb*, Friedhelm Sandbrink, Brigitte Schiller, Eric Storch, David Thomas, Roger Weiss.

*Deceased

© 2024 Dember LM et al. *JAMA Intern Med.*

eTable 1. Eligibility Criteria

<table>
<tr><th colspan="2">Trial Eligibility Criteria</th></tr>
<tr><th>Inclusion Criteria</th><th>Exclusion Criteria</th></tr>
<tr><td>Age ≥18 years</td><td>Current opioid use disorder or other non-tobacco substance use disorder</td></tr>
<tr><td>Undergoing in-center maintenance hemodialysis for ≥90 days</td><td>Current use of heroin</td></tr>
<tr><td>English- or Spanish-speaking</td><td>Current use of methadone, buprenorphine, or naltrexone for opioid use disorder</td></tr>
<tr><td>Chronic pain[a]</td><td>Current receipt of hospice care</td></tr>
<tr><td>PEG score ≥4 (range 0-10)</td><td>Cognitive impairment precludes trial participation</td></tr>
<tr><td>Willing to provide informed consent</td><td>Active suicidal intent[b]</td></tr>
<tr><td>Willing to allow research team to obtain opioid pharmacy refill data</td><td>Unstable bipolar disorder, schizophrenia, post-traumatic stress disorder, or other psychotic disorder</td></tr>
<tr><td rowspan="4">Willing to allow research team to contact and work with their opioid prescriber if prescribed opioids</td><td>Life expectancy <6 months</td></tr>
<tr><td>Anticipated kidney transplantation, transfer to another dialysis facility, or transition to home dialysis within 6 months</td></tr>
<tr><td>Current incarceration</td></tr>
<tr><td>Any other condition viewed by the investigator as precluding participation</td></tr>
<tr><th colspan="2">Buprenorphine Eligibility Criteria</th></tr>
<tr><th>Inclusion Criteria</th><th>Exclusion Criteria</th></tr>
<tr><td>Current use of prescribed opioids at an average dosage of ≥20 MME/day</td><td>Current active addiction to substances other than tobacco, defined as probable moderate-severe substance use disorder for which evidence-based addiction treatment is indicated</td></tr>
<tr><td rowspan="4">For participants of childbearing potential, a negative serum pregnancy test and willingness to use an effective form of contraception or remain abstinent from sexual intercourse with partners of the opposite sex during the buprenorphine administration period</td><td>ALT, AST, or total bilirubin >2X the upper limit of normal</td></tr>
<tr><td>History of long QT syndrome, family history of long QT syndrome, or use of Class IA or Class III anti-arrhythmic medications[c]</td></tr>
<tr><td>Known allergy or prior intolerance to buprenorphine</td></tr>
<tr><td>Current use of buprenorphine</td></tr>
</table>

[a]Chronic pain is defined as a response of “most days” or “every day” to the following question: “In the past 3 months, how often have you had pain?” Answer options: never, some days, most days, every day

[b]Based on an initial screening with the PHQ-9 question #9 followed by positive findings on further evaluation

[c]Class 1A anti-arrhythmic medications include quinidine, procainamide, and disopyramide. Class III anti-arrhythmic medications include amiodarone, sotalol, dofetilide, and dronedarone

Abbreviations: PEG, Pain, Activity and Enjoyment of Life Scale; MME, morphine milligram equivalents; ALT, alanine transaminase; AST, aspartate aminotransferase.

© 2024 Dember LM et al. *JAMA Intern Med.*

eTable 2. Training, Certification, Supervision, and Fidelity Monitoring of PCST Coaches

Initial Training Activities

- Coaches attended two days of intensive training
- Coaches participated in 12 weeks of role playing
- Coaches attended overview of Interactive Voice Response intervention

Certification Prior to Trial Activities

- Coaches audio recorded role playing for each of the 12 coach-led sessions
- PCST leadership team rated the audio recorded role playing sessions on content mastery and treatment integrity

Supervision During Trial

- Coaches met with PCST leadership for weekly case discussions throughout the duration of the coach-led portion of the intervention

Fidelity Assessment

- Independent team of cognitive behavioral therapy experts monitored recorded sessions
- Fidelity to both session content and skilled delivery were reviewed
- Feedback was provided to PCST leadership team at regular intervals
- Ten percent of all sessions were reviewed
- No substantive issues requiring remediation were identified

Abbreviation: PCST, pain coping skills training;

© 2024 Dember LM et al. *JAMA Intern Med.*

eTable 3. Complete List of Outcome Measures

Domain	Instrument or Data Source	Look-Back Period	Score Range	Interpretation of Higher Score	Ascertainment Weeks
**Primary Outcome**					
Pain interference[a]	Brief Pain Inventory (BPI) Interference	1 week	0-10	More interference	0, 12, 24, 36
**Secondary Outcomes – Pain and Opioid Use**					
Pain intensity[a]	Brief Pain Inventory (BPI) Severity	1 week	0-10	More severe	0, 12, 24, 36
Pain catastrophizing[a]	Pain Catastrophizing Scale – SF 6	None	0-24	More catastrophizing	0, 12, 24, 36
Opioid use[a]	Timeline Followback	2 weeks	N/A	--	0, 12, 24, 36
Composite of pain and opioid use[a,b]	BPI Interference / Timeline Followback	1 week / 2 weeks	N/A	--	0, 12, 24, 36
**Secondary Outcomes – Conditions or Symptoms Associated with Pain or Opioid Use**					
Quality of life[a]	Single-Item QOL Scale – McGill Quality of Life	2 days	0-10	Better quality of life	0, 12, 24, 36
Depression[a]	Patient Health Questionnaire (PHQ)-9	2 weeks	0-27	More depression	0, 12, 24, 36
Anxiety[a]	Generalized Anxiety Disorder (GAD)-7	2 weeks	0-21	More anxiety	0, 12, 24, 36
Coping Strategies	Coping Strategies Questionnaire 24 - Single item[c]	None	0-6	More catastrophizing	0, 12, 24, 36
Self-efficacy Pain	PROMIS Self-Efficacy for Managing Chronic Conditions – Managing Symptoms:<br>• Short Form 8A<br>• Single item	None	 8-40<br>1-10	More self-efficacy	0, 12, 24, 36
Sleep quality	PROMIS Sleep Disturbance 6a + Sleep Duration Question	7 days	6-30	Worse sleep	0, 12, 24, 36
Fatigue	PROMIS Fatigue SF 6a	7 days	6-30	Worse fatigue	0, 12, 24, 36
Physical functioning	PROMIS Physical Functioning SF 6b	7 days	6-30	Better physical function	0, 12, 24, 36
Social support	Multidimensional Scale of Perceived Social Support	None	1-7	More support	0, 12, 24, 36
Dialysis-associated symptoms	Dialysis Symptom Index<br>• Number<br>• Severity	1 week	 0-30<br>0-120	More symptoms<br>More severe symptoms	0, 12, 24, 36
Satisfaction with treatment	Patient Global Impression of Change	Since Baseline	0-5	Less improvement	0, 12, 24, 36
Family intrusion	PROMIS Satisfaction with Social Roles and Activities	7 days	8-40	More satisfaction	0, 12, 24, 36
Discrimination	Everyday Discrimination Scale	None	0-25	More discrimination	0, 36
**Secondary Outcomes – Clinical Events**					
Falls[a]	Self-report, medical records	N/A	N/A	--	0 through 36
Hospitalizations[a]	Self-report, medical records	N/A	N/A	--	0 through 36
Death[a]	Dialysis facility personnel, family, medical records	N/A	N/A	--	0 through 36
**Exploratory Outcomes**					
Acceptability of buprenorphine	Proportion of eligible participants who agree to switch to buprenorphine	N/A	N/A	--	24
Tolerability of buprenorphine	Proportion of participants who remain on buprenorphine for the full treatment period	N/A	N/A	--	36
Efficacy of buprenorphine	Brief Pain Inventory (BPI) Interference	1 week	0-10	More interference	24, 36

© 2024 Dember LM et al. *JAMA Intern Med.*

[a]Pre-specified for inclusion in primary results report.
[b]For the composite outcome of pain and opioid use, success is defined as either: 1) less pain with stable or less opioid use, or 2) less opioid use with stable or less pain. Pain was categorized as less if there was a decrease of >1 point on the Brief Pain Inventory (BPI) Interference subscale score, stable if there was a change in BPI Interference of 0-1 point in either direction, and more if there was an increase in BPI Interference of >1 point. Opioid use was categorized as less if there was ≥ 25% reduction in average MME/day, stable if there was <25% reduction and <10% increase in average MME/day, and more if there was ≥10% increase in average MME/day
[c]At Baseline, the full Coping Strategies Questionnaire (CSQ) 24-Item was administered but at all other time points only the single item version of the CSQ-24 was administered. The CSQ 24-Item has 4 subscales, each with a range of 6-42. For the Diversion, Reinterpretation, and Cognitive Coping subscales, a higher score indicates more coping, and for the Catastrophizing subscale, a higher score indicates more catastrophizing. The single-item version of the CSQ-24, has a range of 0-6 with a higher score indicating more catastrophizing.

© 2024 Dember LM et al. *JAMA Intern Med.*

eTable 4. Adherence to Coach-Led PCST Sessions Among Participants Randomized to PCST Group

Completed Sessions per Participant		Completed Sessions: No (%) of Participants				
**Mean (SD)**	**Median (IQR)**	**<6 Sessions**	**≥6 Sessions**	**≥8 Sessions**	**≥10 Sessions**	**12 Sessions**
10.4 (2.8)	12 (10-12)	28 (8.8%)	291 (91.2%)	277 (86.8%)	248 (77.7%)	202 (63.3%)

# Completed PCST Sessions	# Participants
0	3
1	1
2	3
3	10
4	7
5	4
6	6
7	8
8	11
9	18
10	24
11	22
12	202

© 2024 Dember LM et al. *JAMA Intern Med.*

eTable 5. Delivery Method and Location for Coach-Led PCST Sessions

Session Delivery Method	Session Location	# Sessions
Video	Dialysis Facility<br>Home<br>Other<br>Missing	1,771<br>272<br>12<br>8
Telephone	Dialysis Facility<br>Home<br>Other<br>Missing	334<br>868<br>57<br>3
Missing	Dialysis Unit<br>Missing	1<br>7

© 2024 Dember LM et al. *JAMA Intern Med.*

eTable 6. Sensitivity Analysis: Effect of Pain Coping Skills Training on Patient-Reported Outcomes Using Multiple Imputation to Address Missing Outcomes

Outcome	Between-Group Difference[a] Mean (95% CI)
**Week 12**	
Pain Interference: BPI Interference (Primary)<br>Range 0-10<br>Higher=More interference	-0.47 (-0.84, -0.11)
Pain Intensity: BPI Severity<br>Range 0-10<br>Higher=More severe pain	-0.26 (-0.59, 0.07)
Catastrophizing: Pain Catastrophizing Scale SF-6<br>Range 0-24<br>Higher=More catastrophizing	-0.91 (-1.85, 0.02)
Quality of Life: Single-Item QoL<br>Range 0-10<br>Higher=Better quality of life	0.52 (0.05, 1.00)
Depression: PHQ-9<br>Range 0-27<br>Higher=More depression	-0.79 (-1.63, 0.05)
Anxiety: GAD-7<br>Range 0-21<br>Higher=More anxiety	-0.80 (-1.62, 0.02)
Average MME/day[b]<br>Higher=More opioid use	-4.9 (-15.8, 6.0)
**Week 24**	
Pain Interference: BPI Interference (Primary)<br>Range 0-10<br>Higher=More interference	-0.49 (-0.86, -0.13)
Pain Intensity: BPI Severity<br>Range 0-10<br>Higher=More severe pain	-0.51 (-0.85, -0.17)
Catastrophizing: Pain Catastrophizing Scale SF-6<br>Range 0-24<br>Higher=More catastrophizing	-1.50 (-2.47, -0.53)
Quality of Life: Single-Item QoL<br>Range 0-10<br>Higher=Better quality of life	0.39 (-0.10, 0.88)
Depression: PHQ-9<br>Range 0-27<br>Higher=More depression	-0.82 (-1.71, 0.07)
Anxiety: GAD-7<br>Range 0-21<br>Higher=More anxiety	-0.97 (-1.82, -0.13)
Average MME/day[b]<br>Higher=More opioid use	-1.3 (-13.3, 10.7)

© 2024 Dember LM et al. *JAMA Intern Med.*

Outcome	Between-Group Difference[a] Mean (95% CI)
**Week 36**	
Pain Interference: BPI Interference (Primary)<br>Range 0-10<br>Higher=More interference	-0.37 (-0.75, 0.02)
Pain Intensity: BPI Severity<br>Range 0-10<br>Higher=More severe pain	-0.31 (-0.66, 0.04)
Catastrophizing: Pain Catastrophizing Scale SF-6<br>Range 0-24<br>Higher=More catastrophizing	-1.20 (-2.16, -0.24)
Quality of Life: Single-Item QoL<br>Range 0-10<br>Higher=Better quality of life	-0.12 (-0.62, 0.38)
Depression: PHQ-9<br>Range 0-27<br>Higher=More depression	0.03 (-0.86, 0.91)
Anxiety: GAD-7<br>Range 0-21<br>Higher=More anxiety	-0.45 (-1.29, 0.39)
Average MME/day[b]<br>Higher=More opioid use	-2.5 (-13.9, 8.8)

[a]The differences in changes and 95% confidence intervals are calculated using linear mixed effects models with random effects of the stratification factor of enrolling site and participants within each enrolling site. The linear mixed effects models included fixed effects of intervention, time, interactions between intervention and time, and the stratification factor of baseline opioid use.

[b]Among those with opioid use in the last 14 days

Abbreviations: PCST, Pain Coping Skills Training; BPI Interference, Brief Pain Inventory Interference Subscale; BPI Severity, Brief Pain Inventory Severity Subscale; PCS SF-6, Pain Catastrophizing Scale Short Form-6; QoL, Quality of Life; PHQ-9, Patient Health Questionnaire-9; GAD-7, Generalized Anxiety Disorder-7; MME/day, morphine mg equivalent per day

© 2024 Dember LM et al. *JAMA Intern Med.*

eTable 7. Sensitivity Analysis: Comparison of Models with Enrolling Site versus Dialysis Facility as Random Effect (enrolling site was used in primary analysis)

Visit	Outcome	Between-Group Difference in Change from Baseline[a]	
		Enrolling Site[c]	Dialysis Facility[d]
12	BPI Interference	-0.49 (-0.85, -0.12)	-0.48 (-0.85, -0.12)
	BPI Severity	-0.27 (-0.61, 0.06)	-0.27 (-0.60, 0.06)
	PCS SF-6 (Pain Catastrophizing)	-0.93 (-1.86, 0.01)	-0.92 (-1.86, 0.02)
	MME per day[b]	-4 (-18, 10)	-4 (-18, 10)
	Single Item QoL	0.52 (0.04, 1.00)	0.52 (0.04, 0.99)
	PHQ-9 (Depression)	-0.86 (-1.72, -0.01)	-0.87 (-1.72, -0.01)
	GAD-7 (Anxiety) Score	-0.88 (-1.70, -0.05)	-0.87 (-1.69, -0.04)
24	BPI Interference	-0.48 (-0.86, -0.11)	-0.48 (-0.85, -0.10)
	BPI Severity	-0.50 (-0.85, -0.16)	-0.50 (-0.84, -0.16)
	PCS SF-6 (Pain Catastrophizing)	-1.49 (-2.46, -0.52)	-1.49 (-2.45, -0.52)
	MME per day[b]	2 (-13, 17)	1 (-14, 16)
	Single Item QoL	0.40 (-0.09, 0.89)	0.40 (-0.09, 0.89)
	PHQ-9 (Depression)	-0.94 (-1.82, -0.06)	-0.94 (-1.82, -0.06)
	GAD-7 (Anxiety) Score	-0.98 (-1.84, -0.13)	-0.97 (-1.83, -0.11)
36	BPI Interference	-0.34 (-0.72, 0.04)	-0.34 (-0.72, 0.04)
	BPI Severity	-0.28 (-0.63, 0.06)	-0.28 (-0.63, 0.07)
	PCS SF-6 (Pain Catastrophizing)	-1.12 (-2.10, -0.14)	-1.12 (-2.10, -0.14)
	MME per day[b]	-2 (-18, 13)	-2 (-18, 13)
	Single Item QoL	-0.10 (-0.60, 0.40)	-0.11 (-0.60, 0.39)
	PHQ-9 (Depression)	-0.01 (-0.90, 0.88)	-0.01 (-0.90, 0.88)
	GAD-7 (Anxiety) Score	-0.55 (-1.42, 0.31)	-0.54 (-1.41, 0.33)

[a]The differences in change and 95% confidence intervals are calculated using linear mixed effects models with random effects of the stratification factor of enrolling site and participants within each enrolling site. The linear mixed effects models included fixed effects of intervention, time, interactions between intervention and time, and the stratification factor of baseline opioid use.

[b]Among those with opioid use in the last 14 days

[c]Random intercepts include enrolling site and participants within each enrolling site.

[d]Random intercepts include dialysis facility and participants within each dialysis unit.

© 2024 Dember LM et al. *JAMA Intern Med.*

eTable 8. Sensitivity Analysis: Decrease of >30% in BPI Interference Score[a]

	>30% Decrease from Baseline		Odds Ratio (95% CI)[c]
	Pain Coping Skills Training No. (%)	Usual Care No. (%)	
**Week 12**	94 (33.5%)	72 (24.5%)	1.53 (1.06, 2.20)
**Week 24**	97 (37.6%)	75 (28.5%)	1.50 (1.04, 2.17)
**Week 36**	93 (37.3%)	68 (26.7%)	1.60 (1.10, 2.32)

[a]Range 0 – 10; higher score indicates more pain interference
[b]Usual care is the reference group
Abbreviation: PCST, Pain Coping Skills Training, BPI Interference, Brief Pain Inventory Interference Subscale

© 2024 Dember LM et al. *JAMA Intern Med.*

eTable 9. Composite Outcome of Pain Interference and Opioid Use

	Success Based on the Composite Outcome of Pain Interference and Opioid Use[a]		Odds Ratio (95% CI)[b]
	Pain Coping Skills Training # (%)	Usual Care # (%)	
**Week 12**	127 (50.4)	112 (39.6)	1.50 (1.07, 2.11)
**Week 24**	127 (53.1)	117 (46.6)	1.31 (0.92, 1.85)
**Week 36**	117 (49.2)	101 (43.2)	1.28 (0.89, 1.82)

[a]Success is defined as either: 1) less pain with stable or less opioid use, or 2) less opioid use with stable or less pain. Pain was categorized as less if there was a decrease of >1 point on the Brief Pain Inventory (BPI) Interference subscale score, stable if there was a change in BPI Interference of 0-1 point in either direction, and more if there was an increase in BPI Interference of >1 point. Opioid use was categorized as less if there was ≥ 25% reduction in average MME/day, stable if there was <25% reduction and <10% increase in average MME/day, and more if there was ≥10% increase in average MME/day. Opioid use was assessed using timeline followback with a 14-day look-back period.

[b]Usual care is the reference group

Abbreviations: PCST, Pain Coping Skills Training

© 2024 Dember LM et al. *JAMA Intern Med.*

eTable 10. Clinical Event Outcomes

	Pain Coping Skills Training			Usual Care			Rate Ratio (95% CI)[a]
	Participants with Event No. (%)	Total Events No.	Event Rate No. per Pt-Year	Participants with Event No. (%)	Total Events No.	Event Rate No. per Pt-Year	
**Death**	35 (11.0%)	35	0.17	34 (10.5%)	34	0.15	1.08 (0.68, 1.72)
**Hospitalization**	157 (49.2%)	349	1.67	158 (48.8%)	313	1.42	1.17 (1.00, 1.38)
**Fall**	84 (26.3%)	138	0.66	94 (29.0%)	155	0.70	0.94 (0.73, 1.21)

[a]Usual Care is the reference group; Rate ratios were generated using generalized estimating equation (GEE) models with a Poisson distribution as described in the Methods section.

Abbreviations: Pt, participant

eTable 10.a Sensitivity Analysis: Negative Binomial GEE Models to Assess the Impact of Overdispersion

	Rate Ratio (95% CI)[a]
**Death**	1.13 (0.95, 1.35)
**Hospitalization**	1.08 (0.68, 1.72)
**Fall**	0.90 (0.69, 1.18)

[a]Usual Care is the reference group; Rate ratios were generated using generalized estimating equation (GEE) models with a negative binomial distribution, an offset of follow-up time, and a log link. The negative binomial GEE models included intervention and baseline opioid use and treated enrolling sites as clusters with an independent correlation structure.

© 2024 Dember LM et al. *JAMA Intern Med.*

eTable 11. Adverse Events

	Pain Coping Skills Training		Usual Care	
	**Pts with Event No. (%)**	**Events per Pt-Yr**	**Pts with Event No. (%)**	**Events per Pt-Yr**
Serious Adverse Event or Any Adverse Event of Interest	200 (62.7%)	2.57	214 (66.0%)	2.36
Serious Adverse Event	169 (53.0%)	1.83	167 (51.5%)	1.53
Any Adverse Event of Interest	102 (32.0%)	0.88	115 (35.5%)	0.97
Suicidality Alert	11 (3.4%)	0.09	19 (5.9%)	0.10
Opioid Withdrawal Symptoms	1 (0.3%)	0.01	5 (1.5%)	0.05
Event Attributed to New Medication for Pain, Depression, or Anxiety	11 (3.4%)	0.08	9 (2.8%)	0.09
Development of Opioid Use Disorder	0 (0.0%)	0.00	0 (0.0%)	0.00
Development of Non-Opioid Substance Use Disorder	0 (0.0%)	0.00	0 (0.0%)	0.00

Abbreviations: Pts, participants; Pt-Yr, participant-year

© 2024 Dember LM et al. *JAMA Intern Med.*

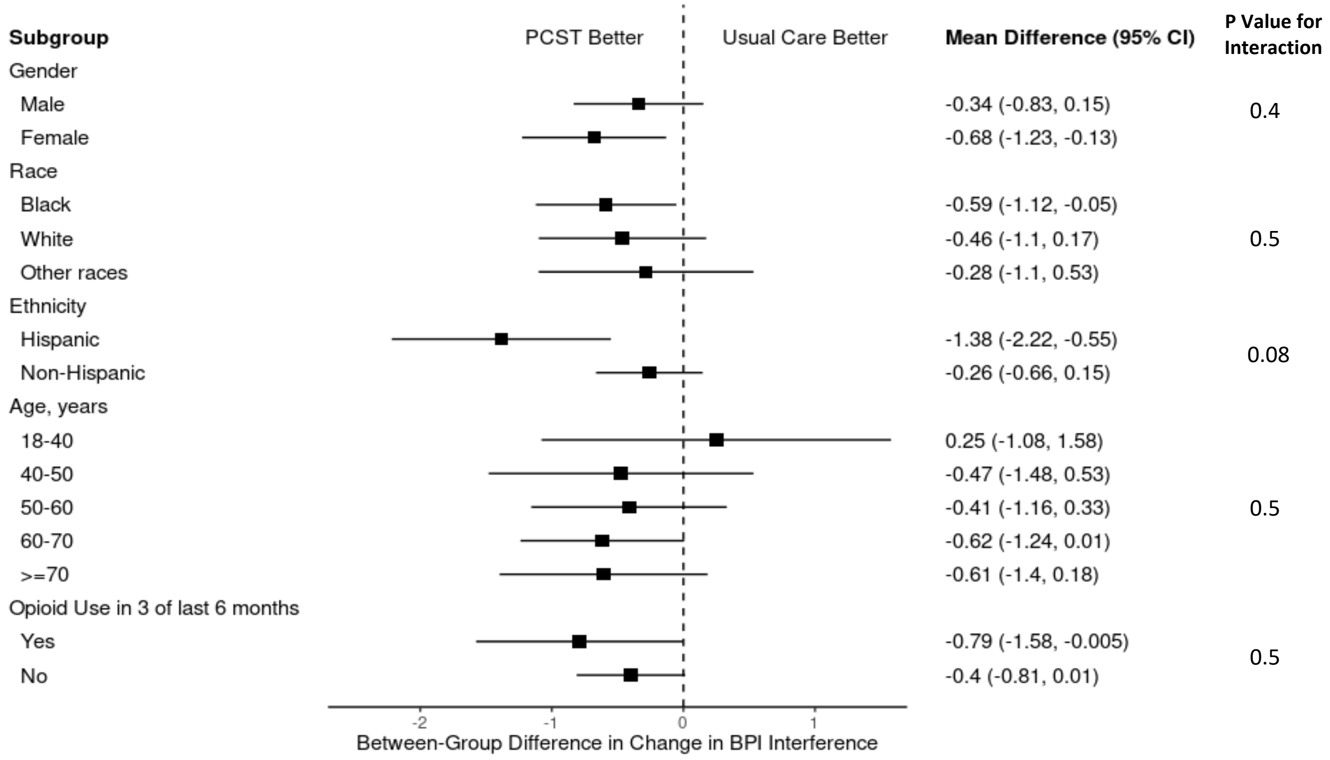

eFigure. Subgroup Analysis of the Primary Endpoint: Change in BPI Interference between Baseline and Week 12

Gender, race, ethnicity, and opioid use were based on participant self-report. Opioid use was based on the response to the quesiton asked at baseline: “Did you use opioid pain medication during at least 3 of the last 6 months?”.

© 2024 Dember LM et al. *JAMA Intern Med.*
