# Supplementary material for: Pain Coping Skills Training for Patients Receiving Hemodialysis: The HOPE Consortium Randomized Clinical Trial
Source: JAMA Intern Med. 2024 Dec 30;185(2):197–207. doi: 10.1001/jamainternmed.2024.7140 (PMC11791705; doi:10.1001/jamainternmed.2024.7140)
Supplement: Supplement 4. — Nonauthor Collaborators. Members of the HOPE Consortium [file jamainternmed-e247140-s004.pdf]

Supplemental Online Content: Nonauthor Collaborators
*First name, last name, and suffix (if applicable) are required and will appear in PubMed.

*Group Name(s): HOPE Consortium

*First Name and Middle Initial(s)	*Last Name	*Suffix (eg, Jr, III)	Academic Degrees	Institution	Location (city, state/province, country)	Role or Contribution, eg, chair, principal investigator	Group (if more than 1 Group listed in the byline) and/or Subgroup (eg, Steering Committee)
Melissa	Adams			VA Portland Healthcare System	Portland, OR		
Stephany	Almonte-Then			University of Pennsylvania	Philadelphia, PA		
Chigozie	Amonu			University of Pennsylvania	Philadelphia, PA		
Lisa	Anderson			University of Washington	Seattle, WA		
Adamaris	Argega Leon			University of New Mexico	Albuquerque, NM		
Christos P	Argyropoulos			University of New Mexico	Albuquerque, NM		
Elena	Ashley			University of New Mexico	Albuquerque, NM		
Monica	Bajana Meza			University of New Mexico	Albuquerque, NM		
Nisha	Bansal			University of Washington	Seattle, WA		
Monica	Barbosa			VA North Texas Healthcare System	Dallas, TX		
Ted	Barrell			University of Pennsylvania	Philadelphia, PA		
Gavin B	Bart			Hennepin Healthcare	Minneapolis, MN		
Amber	Barton			University of Pittsburgh	Pittsburgh, PA		
Scott	Beach			University of Pittsburgh	Pittsburgh, PA		
Levi	Beeks			VA North Texas Healthcare System	Dallas, TX		
Justin M	Belcher			Yale University and VA Connecticut	New Haven, CT/West		
Nathaniel	Berman			Rogosin Institute	New York, NY		
DeVitra	Berry			Vanderbilt University Medical Center	Nashville, TN		
Elenore P	Bhatraju			University of Washington	Seattle, WA		
Nicholas	Bishop			University of Pennsylvania	Philadelphia, PA		
Christopher	Blazes			VA Portland Healthcare System	Portland, OR		
Andrew M	Busch			Hennepin Healthcare	Minneapolis, MN		
Sarah	Cameron			Hennepin Healthcare	Minneapolis, MN		
Monica	Cardona			University of New Mexico	Albuquerque, NM		
Victoria	Casilli			University of Pittsburgh	Pittsburgh, PA		
Miri	Cazes			New York University	New York, NY		
Gabriela	Chacon Palma			University of New Mexico	Albuquerque, NM		
Katy	Chalamidas			University of New Mexico	Albuquerque, NM		

*First name, last name, and suffix (if applicable) are required and will appear in PubMed.

***First Name and Middle Initial(s)**	***Last Name**	***Suffix (eg, Jr, III)**	Academic Degrees	Institution	Location (city, state/province, country)	Role or Contribution, eg, chair, principal investigator	Group (if more than 1 Group listed in the byline) and/or Subgroup (eg, Steering Committee)
Sobaata	Chaudry			New York University	New York, NY		
Nelson	Chen			Rogosin Institute	New York, NY		
Ines	Chicos			Rogosin Institute	New York, NY		
Carlyn	Clark			University of Washington	Seattle, WA		
Deza'Rae	Collins			VA Portland Healthcare System	Portland, OR		
Lynda	Connelly			University of Pittsburgh	Pittsburgh, PA		
Blanca	Contreras			University of Illinois Chicago	Chicago, IL		
Adrian	Cosmin			VA New York Harbor Healthcare System	New York, NY		
Susan T	Crowley			Yale University and VA Connecticut Healthcare System	New Haven, CT/West Haven, CT		
Cheryl	Dalton			West Virginia University	Morgantown, WV		
Jane	Dirks			University of Pittsburgh	Pittsburgh, PA		
Stephanie	Donahue			Rogosin Institute	New York, NY		
Paula	Dutka			New York University	New York, NY		
Veronica	Dyer			New York University	New York, NY		
Oluoma	Edeh			University of New Mexico	Albuquerque, NM		
David A	Edwards			Vanderbilt University Medical	Nashville, TN		
Dawn P	Edwards			Rogosin Institute	New York, NY		
Kome	Ekor			Massachussetts General Hospital	Boston, MA		
Denise	Esserman			Yale University	New Haven, CT		
Caroline G	Falker			Yale University and VA Connecticut Healthcare System	New Haven, CT/West Haven, CT		
Luke	Farkas			University of Pittsburgh	Pittsburgh, PA		
John T	Farrar			University of Pennsylvania	Philadelphia, PA		
Sabrina	Felson			VA New York Harbor Healthcare System	New York, NY		
Yasmine	Flores			New York University	New York, NY		
Daniela	Fraticelli Ortiz			New York University	New York, NY		
George	Garcia			University of New Mexico	Albuquerque, NM		
Lindsay	Gear			University of New Mexico	Albuquerque, NM		
Ana	Geibel			University of Pittsburgh	Pittsburgh, PA		

Supplemental Online Content: Nonauthor Collaborators
*First name, last name, and suffix (if applicable) are required and will appear in PubMed.

***First Name and Middle Initial(s)**	***Last Name**	***Suffix (eg, Jr, III)**	Academic Degrees	Institution	Location (city, state/province, country)	Role or Contribution, eg, chair, principal investigator	Group (if more than 1 Group listed in the byline) and/or Subgroup (eg, Steering Committee)
Cheryl	Gilmartin			University of Illinois Chicago	Chicago, IL		
David S	Goldfarb			VA New York Harbor Healthcare System	New York, NY		
Keith S	Goldfeld			New York University	New York, NY		
Amanda J	Goldstein			University of Illinois Chicago	Chicago, IL		
Anna	Gong			Rogosin Institute	New York, NY		
Candace	Grant			New York University	New York, NY		
Robert E	Grindstaff			Vanderbilt University Medical Center	Nashville, TN		
Erik	Guajardo			VA North Texas Healthcare System	Dallas, TX		
Cameron	Guy			University of New Mexico	Albuquerque, NM		
Megan	Hamm			University of Pittsburgh	Pittsburgh, PA		
Jenika	Hammond			Durham VA Healthcare System	Durham, NC		
Khritian	Harris			Durham VA Healthcare System	Durham, NC		
Sara	Hoffman			Durham VA Healthcare System	Durham, NC		
Christopher	Holden			University of Illinois Chicago	Chicago, IL		
Heather	Howell			VA Connecticut Healthcare System	West Haven, CT		
T. Alp	Ikizler			Vanderbilt University Medical Center	Nashville, TN		
Steven	Joffe			University of Pennsylvania	Philadelphia, PA		
Jonah	Joffe			University of Pennsylvania	Philadelphia, PA		
Sydney	Johnson			University of Washington	Seattle, WA		
Eshika	Kalam			Rogosin Institute	New York, NY		
Kyle M	Kampman			University of Pennsylvania	Philadelphia, PA		
Colin	Keane			New York University	New York, NY		
Grace	Kimura			University of New Mexico	Albuquerque, NM		
Olivia	Kirsch			University of Pittsburgh	Pittsburgh, PA		
Wissam M	Kourany			Durham VA Healthcare System	Durham, NC		
Pragna	Krishnamurthy			New York University	New York, NY		
Paula	Kubrick			University of Pittsburgh	Pittsburgh, PA		
Prescious	Lacey			University of Pittsburgh	Pittsburgh, PA		

Supplemental Online Content: Nonauthor Collaborators
*First name, last name, and suffix (if applicable) are required and will appear in PubMed.

*First Name and Middle Initial(s)	*Last Name	*Suffix (eg, Jr, III)	Academic Degrees	Institution	Location (city, state/province, country)	Role or Contribution, eg, chair, principal investigator	Group (if more than 1 Group listed in the byline) and/or Subgroup (eg, Steering Committee)
J Richard	Landis			University of Pennsylvania	Philadelphia, PA		
James P	Lash			University of Illinois Chicago	Chicago, IL		
Kaeleb	Lazlo			University of Washington	Seattle, WA		
Joshua D	Lee			New York University	New York, NY		
James P	Lefler			Durham VA Healthcare System	Durham, NC		
Alexander	Leon Cupe			University of New Mexico	Albuquerque, NM		
Stephen	Lerner			New York University	New York, NY		
Jane	Liebschutz			University of Pittsburgh	Pittsburgh, PA		
Patricia	Lietz			University of Pittsburgh	Pittsburgh, PA		
Lori	Linke			University of Washington	Seattle, WA		
Mark B	Lockwood		PhD	University of Illinois Chicago	Chicago, IL		
Henry	Luo			University of New Mexico	Albuquerque, NM		
Ashley	Macina			New York University	New York, NY		
Angela	McCarthy			New York University	New York, NY		
Katherine	McDaniels			University of New Mexico	Albuquerque, NM		
Sophia	McLaren			University of New Mexico	Albuquerque, NM		
Daniel W	McNeil			West Virginia University	Morgantown, WV		
Monya	Meinel			University of Illinois Chicago	Chicago, IL		
Valeria	Mejia			University of New Mexico	Albuquerque, NM		
Beza	Mengesha			Massachussetts General Hospital	Boston, MA		
Don	Merriman			Vanderbilt University Medical Center	Nashville, TN		
Wambua	Michael			Hennepin Healthcare	Minneapolis, MN		
Roger	Mims			West Virginia University	Morgantown, WV		
Puneet	Mishra			Vanderbilt University Medical Center	Nashville, TN		
Frank	Modersitzki			VA New York Harbor Healthcare System	New York, NY		
Alvin H	Moss			West Virginia University	Morgantown, WV		
Claire	Mullins			University of New Mexico	Albuquerque, NM		
Ursula	Munet			Hennepin Healthcare	Minneapolis, MN		
Kristy	O'Connell			West Virginia University	Morgantown, WV		

Supplemental Online Content: Nonauthor Collaborators
*First name, last name, and suffix (if applicable) are required and will appear in PubMed.

***First Name and Middle Initial(s)**	***Last Name**	***Suffix (eg, Jr, III)**	Academic Degrees	Institution	Location (city, state/province, country)	Role or Contribution, eg, chair, principal investigator	Group (if more than 1 Group listed in the byline) and/or Subgroup (eg, Steering Committee)
Uchechukwu	Okereke			University of New Mexico	Albuquerque, NM		
Donna	Olejniczak			University of Pittsburgh	Pittsburgh, PA		
Samuel	Opeke			Vanderbilt University Medical Center	Nashville, TN		
Maria	Pacheco-Hernandez			Hennepin Healthcare	Minneapolis, MN		
Shane	Pankratz			University of New Mexico	Albuquerque, NM		
Diane	Park			University of Pennsylvania	Philadelphia, PA		
Kevin	Payne			VA Connecticut Healthcare System	West Haven, CT		
Hadassah	Pegues			Vanderbilt University Medical Center	Nashville, TN		
Shanannssa	Percy			Massachussetts General Hospital	Boston, MA		
Sarah	Pleasant			Vanderbilt University Medical Center	Nashville, TN		
Teresa	Purdy			Durham VA Healthcare System	Durham, NC		
Davin	Quinn			University of New Mexico	Albuquerque, NM		
Nina	Quintana			University of Illinois Chicago	Chicago, IL		
Kathleen	Rice			New York University	New York, NY		
Grace	Robinson			New York University	New York, NY		
Christopher	Roche			Vanderbilt University Medical Center	Nashville, TN		
Giselle	Rodriguez Sosa			University of New Mexico	Albuquerque, NM		
Gabriel	Rudow			University of New Mexico	Albuquerque, NM		
Qamhiyeh	Rudy			Hennepin Healthcare	Minneapolis, MN		
Jeanette	Rutledge			Durham VA Healthcare System	Durham, NC		
Sindi	Sanchez			VA North Texas Healthcare System	Dallas, TX		
Brian	Sands			VA New York Harbor Healthcare	New York, NY		
Jennifer S	Scherer			New York University	New York, NY		
Mary	Schopp			University of Pittsburgh	Pittsburgh, PA		
Brandon	Self			University of Pittsburgh	Pittsburgh, PA		
Amanda J	Shallcross			New York University	New York, NY		

Supplemental Online Content: Nonauthor Collaborators
*First name, last name, and suffix (if applicable) are required and will appear in PubMed.

*First Name and Middle Initial(s)	*Last Name	*Suffix (eg, Jr, III)	Academic Degrees	Institution	Location (city, state/province, country)	Role or Contribution, eg, chair, principal investigator	Group (if more than 1 Group listed in the byline) and/or Subgroup (eg, Steering Committee)
Kimberly	Silva			University of Illinois Chicago	Chicago, IL		
Taylor	Stallings			University of Pennsylvania	Philadelphia, PA		
Christopher S	Stauffer			VA Portland Healthcare System	Portland, OR		
Alana D	Steffen			University of Illinois Chicago	Chicago, IL		
Thomas	Stewart			Vanderbilt University Medical Center	Nashville, TN		
Susan	Stringfellow			University of Pittsburgh	Pittsburgh, PA		
Lily	Sullivan			University of New Mexico	Albuquerque, NM		
Richard	Torres			VA Portland Healthcare System	Portland, OR		
John	Torres			University of New Mexico	Albuquerque, NM		
Greg	Trejo			University of New Mexico	Albuquerque, NM		
Dalila	Varela			New York University	New York, NY		
Svetlana	Vassilieva			Yale University and VA Connecticut Healthcare System	New Haven, CT/West Haven, CT		
Hugo	Vilchis			University of New Mexico	Albuquerque, NM		
Darlene	Villareal			University of Illinois Chicago	Chicago, IL		
Joanna	Walsh			University of Pennsylvania	Philadelphia, PA		
Javaughn	Ways			New York University	New York, NY		
Tammy	Weidner			University of New Mexico	Albuquerque, NM		
Melissa	Weimer			University of Pittsburgh	Pittsburgh, PA		
Daniel E	Weiner			Tufts Medical Center	Boston, MA		
Steven D	Weisbord			University of Pittsburgh	Pittsburgh, PA		
Caroline	Wilkie			University of Pennsylvania	Philadelphia, PA		
Maryanne	Wilkinson			West Virginia University	Morgantown, WV		
Sonya	Williams			Vanderbilt University Medical Center	Nashville, TN		
Joel	Williams			Hennepin Healthcare	Minneapolis, MN		
Olivia	Wilson			University of Pittsburgh	Pittsburgh, PA		
Vincent	Wood			University of Pittsburgh	Pittsburgh, PA		
Jonathan G	Yabes			University of Pittsburgh	Pittsburgh, PA		
Guillermo	Zamora			University of Illinois Chicago	Chicago, IL		
Samara	Zaniga			VA Connecticut Healthcare System	West Haven, CT		
