# Supplementary material for: Pain Coping Skills Training for Patients Receiving Hemodialysis: The HOPE Consortium Randomized Clinical Trial
Source: JAMA Intern Med. 2024 Dec 30;185(2):197–207. doi: 10.1001/jamainternmed.2024.7140 (PMC11791705; doi:10.1001/jamainternmed.2024.7140)
Supplement: Supplement 5. — Data Sharing Statement [file jamainternmed-e247140-s005.pdf]

Data Sharing Statement

Dember. Pain Coping Skills Training for Patients Receiving Hemodialysis. *JAMA Intern Med*. Published December 30, 2024. doi:10.1001/jamainternmed.2024.7140

Data
Additional Information: Clinicaltrials.gov NCT04571619
Data available: Yes
Data types: Deidentified participant data, Data dictionary
How to access data: The NIDDK Central Repository https://repository.niddk.nih.gov/home/
When available: beginning date: 07-01-2025

Supporting Documents
Document types: Informed consent form
How to access documents: 1. The NIDDK Central Repository https://repository.niddk.nih.gov/home/ 2. Clinicaltrials.gov https://clinicaltrials.gov/
When available: beginning date: 12-21-2024

Additional Information
Who can access the data: Researchers whose proposed use of the data has been approved by the NIDDK Central Repository
Types of analyses: Purposes approved by the NIDDK Central Repository
Mechanisms of data availability: After approval by the NIDDK Central Repository
